# Translating natural genetic variation to gene expression in a computational model of the *Drosophila* gap gene regulatory network

**DOI:** 10.1371/journal.pone.0184657

**Published:** 2017-09-12

**Authors:** Vitaly V. Gursky, Konstantin N. Kozlov, Ivan V. Kulakovskiy, Asif Zubair, Paul Marjoram, David S. Lawrie, Sergey V. Nuzhdin, Maria G. Samsonova

**Affiliations:** 1 Theoretical Department, Ioffe Institute, Saint Petersburg, Russia; 2 Systems Biology and Bioinformatics Laboratory, Peter the Great Saint Petersburg Polytechnic University, Saint Petersburg, Russia; 3 Engelhardt Institute of Molecular Biology, Moscow, Russia; 4 Vavilov Institute of General Genetics, Moscow, Russia; 5 Center for Data-Intensive Biomedicine and Biotechnology, Skolkovo Institute of Science and Technology, Moscow, Russia; 6 Molecular and Computational Biology, University of Southern California, Los Angeles, California, United States of America; University of Iceland, ICELAND

## Abstract

Annotating the genotype-phenotype relationship, and developing a proper quantitative description of the relationship, requires understanding the impact of natural genomic variation on gene expression. We apply a sequence-level model of gap gene expression in the early development of *Drosophila* to analyze single nucleotide polymorphisms (SNPs) in a panel of natural sequenced *D. melanogaster* lines. Using a thermodynamic modeling framework, we provide both analytical and computational descriptions of how single-nucleotide variants affect gene expression. The analysis reveals that the sequence variants increase (decrease) gene expression if located within binding sites of repressors (activators). We show that the sign of SNP influence (activation or repression) may change in time and space and elucidate the origin of this change in specific examples. The thermodynamic modeling approach predicts non-local and non-linear effects arising from SNPs, and combinations of SNPs, in individual fly genotypes. Simulation of individual fly genotypes using our model reveals that this non-linearity reduces to almost additive inputs from multiple SNPs. Further, we see signatures of the action of purifying selection in the gap gene regulatory regions. To infer the specific targets of purifying selection, we analyze the patterns of polymorphism in the data at two phenotypic levels: the strengths of binding and expression. We find that combinations of SNPs show evidence of being under selective pressure, while individual SNPs do not. The model predicts that SNPs appear to accumulate in the genotypes of the natural population in a way biased towards small increases in activating action on the expression pattern. Taken together, these results provide a systems-level view of how genetic variation translates to the level of gene regulatory networks via combinatorial SNP effects.

## Introduction

The analysis of molecular phenotypes is expected to bridge the gap between genotypic and phenotypic variation, thereby facilitating both basic and health research [1, 2]. Most approaches for understanding how genetic variation affects molecular phenotype use statistical tools, like the Genome Wide Association Study (GWAS) paradigm. However, as our understanding of biological systems deepens, it has become possible to build on the foundation of gene regulatory networks, in which “-omics” data allow us to develop a quantitative model underlying the genotype-to-phenotype map. As opposed to GWAS, which is focused on capturing associations, an advantage of this alternative approach is that we can infer specific mechanisms, or causal relations, linking variation in the regulatory DNA to its effect on gene expression patterns [3]. We apply this approach in the context of the gap gene network involved in early development of *Drosophila melanogaster*.

Transcription of developmental genes is coordinated by collections of binding sites for sequence-specific DNA binding proteins, combined in *cis*-regulatory modules (CRMs). Much experimental progress has been made in annotating *Drosophila* CRMs [4–6] and exploring the details of their function [7–9]. Regulation of developmental genes is complex in terms of the spatio-temporal coordination of expression and provided by variable regulatory mechanisms [10, 11]. This regulation can be domain specific, where some CRMs contribute to the anterior expression domain and others to the posterior, accompanied with a nonuniform temporal distribution of those contributions [9]. The regulatory role can also vary, as in the case of Hunchback (Hb) that bifunctionally regulates *even-skipped* (*eve*) stripe 7 expression through two separate “shadow enhancers”, activating one of them and repressing the other [7].

The expression patterns may also experience intraspecific variation. *eve* expression scales with egg size, showing the proportionality of *eve* stripe placement to embryo length [12]. Further studies of small and large embryos revealed an increased variability of stripe placement [13]. Measured natural variation of *eve* expression in three inbred lines of *Drosophila melanogaster* revealed an essential difference in spacing, amplitude, and timing of formation of the expression pattern [14]. This variation in *eve* expression was even larger than that observed between *Drosophila melanogaster* and *Drosophila pseudoobscura* and was provisionally attributed to the effect of a deletion in an intron of the gene *knirps*. The fact that large variation in expression of developmentally important genes does not lead to segmentation defects, viability, or fertility of adult flies may imply the action of buffering mechanisms active in later stages of development.

More data has become available on how mutations in regulatory sequence can influence gene expression through changing transcription factor (TF) binding [15]. The analysis of polymorphism in several CRMs and various *D. melanogaster* populations showed high frequency deletions removing two strong binding sites for Hb, despite the sites being conserved across *Drosophila* species [16]. These deletions affect developmental time in a non-deleterious way and have been suggested to reflect developmental system drift [16], a phenomenon that consists in changing the wiring of a development gene network over evolutionary time without strong perturbations of expression patterns [17, 18]. A tremendous progress in functional annotation of various mutations was associated with developing experimental methods proposed for parallel measurement of promoter activity *in vitro* [19] and *in vivo* [20–22]. With the help of a designed library containing thousands of yeast promoters, gene expression was measured for various combinations of transcription factor binding sites (TFBSs) having different affinities, total number, location in a promoter, and orientation [20]. It was found that the relationship between gene expression and TFBS number is well described by a logistic function, exhibiting fast saturation. Both the number and the local vicinity of TFBSs play a role, but the identity of TFBS is more important in determining high expression [20].

Analysis of effects on expression from single nucleotide substitutions may lead to different conclusions about the magnitude of these effects. For some mammalian enhancers, very few mutations yielded significant perturbations in expression [21, 22]. In contrast to these results, it was shown in another study that majority of single nucleotide substitutions in a mouse CRM alter its function, thus hypothesising about the fine-tuning of this CRM [23]. This study also reported only a modest correlation between affinity and observed expression for mutated TFBSs and importance of creation of novel sites.

In the current paper, we evaluate the effects of natural DNA variation on the modelled performance of gap genes during early development. The segmentation network that patterns the *Drosophila* blastoderm embryo appears ideal for this purpose. Decades of genetic work have identified all of the important genes in this system, and provided a well-supported network topology, making the modeling more reliable [10, 11]. While early models of gene expression often ignored information concerning the CRM sequence [24–26], more recently a thermodynamic approach has been developed in which sequence information regarding individual CRMs is incorporated [27–33]. An extension of this approach was undertaken and validated in our model for the gap gene regulatory network [34]. This model explicitly links the genetic organization of regulatory regions to the expression patterns of gap genes. We use a large panel of 213 sequenced *Drosophila* genotypes from two populations (Raleigh, NC, and Winters, CA [35]) and identify all SNPs in the regulatory regions of the gap genes, which are part of the segmentation gene system. We then apply the gene expression model to determine the functional consequences of these SNPs on expression level.

We aim to answer several specific questions in this study. One concerns understanding the type of action that a given SNP exerts on gene expression. Any mutation of the regulatory sequence may lead to either increasing or decreasing expression in a cell, so in this way we can assign the mutation as either activating or repressing. Analyzing gene expression in multiple cells and at multiple time-points, we may find that a SNP appears as an activator in some conditions and a repressor in others, thus demonstrating an alternating type of action. We elucidate the mechanisms shaping the activating and repressing actions of SNPs and their spatio-temporal distribution.

Another question is how the effects of combinations of polymorphisms aggregate on gene expression state. A similar question is relevant for functional combination of CRMs, as a phenotypic state often results from the interplay of multiple CRMs. Based on the studies of primary and shadow enhancers of the genes *hunchback*, *knirps*, and *snail*, it was shown that CRMs interacting with the target promoter weakly (‘weak enhancers’) combine their effects on gene expression additively, while strong CRMs exhibit a non-linear (sub-additive) combination [8]. It was also demonstrated that single nucleotide mutations in TFBSs within yeast and mammal promoters show complex interactions in their effects on expression, and these effects combine in a non-linear (non-additive) way [20, 23]. The thermodynamic approach in modeling gene expression generally provides a non-linear map accounting for the aggregating effects from multiple SNPs. We quantify these effects for the gap gene system both analytically and numerically and show that the naturally occurring SNPs demonstrate a more linear response in expression level than expected by chance.

Finally, we investigate for signs of purifying selection in the considered populations. The action of natural selection on noncoding DNA in *Drosophila* was previously demonstrated [36, 37]. Analysis of SNPs in TFBSs in two *Drosophila* species showed that positive selection has an effect on TFBS gain and loss (turnover), with purifying selection contributing to its maintenance [38]. Thermodynamic considerations have previously helped to clarify evolutionary aspects of *Drosophila* regulatory elements in related species [39–41]. Martinez et al. [41] studied evolution of the *Drosophila* *eve* stripe 2 enhancer using an expression model and data on expression of reconstructed ancestral sequences of this CRM. They demonstrated and analyzed in great details functional compensations between the binding sites for several TFs. In particular, their results indicate importance of various subtle effects to such compensations, including change in binding affinity and appearance of site overlap over evolutionary time.

The use of gene expression models in such studies is constrained by an important problem arising from the fact that one does not *a priori* know the applicability limits for a model formulated and validated on wild-type data. The evolutionary distance between species can be large enough to be associated with high relative levels of polymorphism, and this will most likely make it difficult to apply a model validated for one species to data for the other species. One possible solution would be to fit the model to both species simultaneously [41], but the necessary data is not always available. We utilize a different approach and contrast the effects from naturally occurring SNPs with those from SNPs chosen randomly from the set of all possible mutations. In this regard, we stay in a reasonable ‘vicinity’ of the wild-type conditions. As a result, we show that the evidence on purifying selection can reliably be detected only for combinations of SNPs, and not for single SNPs. This evidence is also vague if the binding affinities for TFBSs are used as a phenotypic characteristic. In addition, we demonstrate a bias in gene expression perturbations for the studied genotypes.

## Methods

### Model of gene expression

We analyze the regulatory effects of both single SNPs and combinations of SNPs using the previously reported sequence-level model of gap gene expression in early *D. melanogaster* embryos [34]. The model input is information about regulatory regions in the *D. melanogaster* genome, binding motifs for transcription factors (TFs) regulating gap gene expression, and concentrations of these factors in the embryos. The model then predicts gap gene expression dynamics at the RNA and protein levels at each nucleus in the blastoderm-stage embryos during early development (cleavage cycles 13 through 14A). The model describes expression of gap genes *hunchback* (*hb*), *Kruppel* (*Kr*), *giant* (*gt*), and *knirps* (*kni*), and consists of the following equations:

$$\begin{array}{c} \frac{du_{i}^{a}}{dt}=R_{u}^{a}E_{i}^{a}\left(t\right)-\lambda_{u}^{a}u_{i}^{a}+D_{u}^{a}\left(n\right)\left[\left(u_{i-1}^{a}-u_{i}^{a}\right)+\left(u_{i+1}^{a}-u_{i}^{a}\right)\right], \end{array}$$ (1)

$$\begin{array}{c} \frac{dv_{i}^{a}}{dt}=R_{v}^{a}u_{i}^{a}\left(t-\tau^{a}\right)-\lambda_{v}^{a}v_{i}^{a}+D_{v}^{a}\left(n\right)\left[\left(v_{i-1}^{a}-v_{i}^{a}\right)+\left(v_{i+1}^{a}-v_{i}^{a}\right)\right], \end{array}$$ (2)

where $u_{i}^{a}$ and $v_{i}^{a}$ are mRNA and protein concentrations, respectively, of the products of gene *a* in nucleus *i*, $R_{v}^{a}$ and $R_{u}^{a}$ are maximum synthesis rates, $D_{v}^{a}$ and $D_{u}^{a}$ are diffusion coefficients, $\lambda_{v}^{a}$ and $\lambda_{u}^{a}$ are decay rates, *n* is the cleavage cycle number, and the *τ*^*a*^ are time delay parameters accounting for the delay between transcription initiation and protein appearance. The function $E_{i}^{a}\left(t\right)$ describes the probability of transcriptional activation (the fractional occupancy of the promoter by the basal transcriptional machinery (BTM)) of gene *a* in nucleus *i* at time *t*, given the experimental concentrations of TFs and their binding sites. This is calculated using a thermodynamic approach, in which we quantify possible regulatory influence on gap genes from all configurations of the regulatory sequence of these genes. In what follows, we describe briefly the derivation of $E_{i}^{a}\left(t\right)$ omitting the indices *a* and *i* and time variable *t* for brevity; the full details can be found elsewhere [30, 34].

We consider a complex consisting of the regulatory region of a gene together with its basal promotor and analyze all possible configurations of the regulatory region associated with the BTM either bound to the promoter (the ‘ON’ state of the complex) or not bound (the ‘OFF’ state). A configuration of the regulatory region is defined as a set of free and occupied transcription factor binding sites (TFBSs) in this region. It is indexed by a vector *σ* = {*σ*(*s*)}, where *σ*(*s*) = 0 if TFBS *s* is free and *σ*(*s*) = 1 if this site is occupied by its TF. We calculate the statistical weight *q*_*s*_ of the occupancy of TFBS *s* as *q*_*s*_ = *Kv*_dat_ exp (*P*_*s*_), where *K* is the association constant for a strongest TFBS (one constant per TF), *v*_dat_ is the experimental concentration of the TF binding to site *s*, and *P*_*s*_ is a PWM (positional weight matrix) score of site *s* (see S1 Text for details).

Configuration *σ* of the regulatory region has statistical weight *W*_*σ*_ = ∏_*s*_ (*C*_*s*_*q*_*s*_)^*σ*(*s*)^, where the exponentiation to the power of *σ*(*s*) guarantees that only the weights *q*_*s*_ of occupied TFBSs are present in *W*_*σ*_. The parameter *C*_*s*_ is a product of constants quantifying possible interactions between TFs bound to site *s* and to other sites in a given sequence range. There are two types of such local interactions considered in the model: cooperative binding and short-range repression. If site *s* binds TF cooperatively with neighboring sites *j* of the same TF, we have *C*_*s*_ = ∏_*j*_*ω*^*σ*(*j*)^ with the cooperativity parameter *ω* > 1 (one parameter per TF). According to the short-range repression mechanism, TFBSs occupied by repressor TFs may forbid binding in the local vicinity of these sites. If site *s* binds TF-repressor and is in the effective state in configuration *σ* (i.e., no TFBSs are allowed to bind in the vicinity), we have *C*_*s*_ = *β*, where *β* is a factor quantifying the repression efficiency (one parameter per TF-repressor and target gene). The cooperativity and repression parameters are multiplied in *C*_*s*_ if the corresponding mechanisms coexist for a given site and configuration.

We estimate the statistical weights of the occupied state of the basal promotor in the context of the regulatory region. When a configuration *σ* leads to the promoter being occupied by the BTM, we get the statistical weight *W*_*σ*_*Q*_*σ*_ of such a state of the complex, where *Q*_*σ*_ accumulates parameters *α* > 1 (the activation efficiency; one constant for each activator TF and target gene) from each activating binding site occupied in configuration *σ*. When a configuration *σ* is associated with the free state of the promotor, the weight of this state of the complex is *W*_*σ*_. The probability of transcriptional activation is then defined as a fractional occupancy of the basal promotor in the complex with the regulatory region:

$$\begin{array}{c} E=\frac{Z_{ON}}{Z_{ON}+Z_{OFF}},\;Z_{OFF}=\sum_{\sigma }\;W_{\sigma },\;Z_{ON}=\sum_{\sigma }\;W_{\sigma }Q_{\sigma }, \end{array}$$ (3)

where ‘ON’ and ‘OFF’ designations are defined above, and *Z*_*ON*_ and *Z*_*OFF*_ sum up statistical weights of all possible states of the complex with the BTM bound and not bound to the basal promotor, respectively [30, 34].

Values of free parameters in the model were obtained by fitting the model output to the wild type gap gene expression data at cellular resolution [42] using the *D. melanogaster* reference genome, as described in [34]. The obtained parameter values were validated by several means [34]. The model scores on the testing data in a cross-validation analysis did not reveal a statistically significant difference with the fitting results for the full data. On the other hand, such a difference was observed between the the fitting results for the full data and for a set of nonsense data, in which the expression patterns were shuffled between gap genes (‘negative control’). There was a good correspondence between the topology of the regulatory network predicted by the model and that found in previous studies [10]. The model output for all good sets of parameter values was tested on the expression data for various experimentally characterized enhancers and for the *Kr*^−^ environment, and a parameter set exhibiting the best results was selected for further analysis as a representative set (S1 Table). Finally, a local sensitivity analysis for the selected parameter set revealed that only a few of the parameter values can be judged as poorly identifiable (those parameters are marked with an asterisk in S1 Table).

### Regulatory sequences and binding sites

We analyze the SNPs in TFBSs from regulatory regions of gap genes *hb*, *Kr*, *gt*, and *kni*. For putative regulatory regions, we consider genomic segment spanning 12 kbp upstream and 6 kbp downstream of the transcription start site for each gene in the reference *D. melanogaster* genome (dm3 / BDGP5). These regions comprise the classical developmental CRMs of gap genes according to the RedFly database [43]. Binding sites for Hunchback (Hb), Kruppel (Kr), Giant (Gt), Knirps (Kni), Bicoid (Bcd), Caudal (Cad), Tailless (Tll), and Huckebein (Hkb) TFs are predicted using positional weight matrices (PWMs) from [44] (http://autosome.ru/iDMMPMM) with score thresholds selected as in [45] (mean + 3 s.d. of PWM score distribution for all possible *k*-mers). Among these TFBSs, we include in the model only binding sites located in accessible parts of the chromatin according to the DNAse I accessibility data (stage 5 of early development, FDR 5% euchromatic accessible regions, downloaded from UCSC Genome Browser) [46]. There are in total 889 TFBSs included in the model, which we call the ‘model binding sites’. The accessible regions encompass several disjoint pieces of DNA and comprise the classical developmental CRMs. We do not use any specific assumptions about incorporation of these disjoint regions in the model. Each TFBS has a potential to affect transcription activation regardless of the accessible region in which it appears. We call TFBSs of activators (repressors) activating (repressing) TFBSs throughout the text.

As part of our analysis, we wish to compare the level of polymorphism in the model TFBSs with that in the other parts of the regulatory sequence. It is known that TFBSs with a regulatory function are expected to have reduced polymorphism as compared to other non-coding sequences [47]. We perform this comparison in the specified vicinity of the gap genes and use this analysis as a control for our method for selecting TFBSs for the model. For this purpose, in addition to the model TFBSs we consider all potential binding sites which have PWM scores higher than the threshold described above but lie outside of the DNase I accessibility regions. There are 3030 such ‘non-model binding sites,’ i.e. putative sites not included in the model. Additionally, we define as ‘non-functional sequence’ the regions of the considered genomic segment that do not include the model TFBSs, the non-model TFBSs, and the coding sequences for all gap genes. We note that the label ‘non-functional sequence’ should be considered only in the context of the gap gene network, as this sequence could contain regulatory elements responsible for other biological functions. We further consider two disjoint parts of this non-functional sequence: the subset that is ‘accessible’ (i.e. that is in DNase I accessibility regions), and the subset that is ‘non-accessible’ (i.e. not in DNase I accessibility regions). The total length of the analyzed sequence data is 5552 bp for the model TFBSs, 19233 bp for the non-model sites, and a further 36625 bp of non-functional sequence (8227 bps of which are accessible, while 28398 bps are non-accessible).

### SNP data

We use a SNP data set, (referred to as the ‘study population’), initially consisting of 216 sequenced genotypes from two populations of *D. melanogaster*, Raleigh, NC, and Winters, CA [35]. Polymorphic positions in the regulatory regions of the four gap genes are collected and filtered as follows. First, positions with more than two alleles are removed to simplify the subsequent analysis (among 30 such positions in the four regulatory regions, only 3 appear within TFBSs). Next, two genotypes with insufficient coverage are excluded. Finally, the heterozygous sites are relabeled as missing data, since our gene expression model does not allow for heterozygous data. The resulting data has sequence information for between 62 and 213 genotypes per SNP. To calculate the SNP density, we exclude all positions with sequence information for fewer than 170 genotypes. To unify sampling variance in our subsequent bootstrapping procedure, we down-sample all other positions to this same depth (number of genotypes): for SNPs at positions having more than 170 genotypes, a 170 allele subset is chosen randomly. If a SNP is no longer polymorphic after this random resampling, that position is considered as non-polymorphic. However, when we evaluate SNP influence on TF binding and on gene expression, we omit the resampling step and keep all polymorphic positions for the analysis (because no bootstrapping is needed here).

### SNP density calculation

We aim to compare the level of polymorphism in TFBSs with that in the non-functional sequence. Several complicating factors should be taken into account in order to ensure a fair comparison. First, the full sequence of binding sites typically has a smaller length than that of the non-functional sequence, so these lengths should be normalized for polymorphism estimation. Further, the GC content of the non-functional sequence is 45%, compared to 38% and 33% for the model and non-model binding sites, respectively. The higher GC content of the non-functional sequence means that this sequence is expected to exhibit a higher mutation rate, on average, compared to the binding sites [48]. This increases the relative density of polymorphism in these regions, which will obscure the putative effects of purifying selection on the binding sites (which is expected to reduce SNP density). Finally, possible spatial variations in the rates of mutation and recombination may influence the relative density of polymorphisms.

Given these considerations, when comparing the SNP densities between TFBSs and other regions, we control for these confounding factors by applying a modified version of a bootstrap procedure due to [49]. We first pair each nucleotide in the model TFBSs with a maximal set of nucleotides from each other region (non-model binding sites, non-functional sequence, and the accessible and non-accessible parts of the latter), under the constraint that each pair is required to have the same major allele at that position. We then randomly sample nucleotide pairs, with replacement, first drawing a nucleotide from the model TFBSs and then randomly picking one of its possible partners from the other regions. The sampling is performed until the number of random pairs drawn equals the length of the model TFBSs. The SNP densities are then estimated as the relative number of polymorphic positions in the resulting sets of data for each of the regions. This process matches the GC content in all sequence regions under comparison and normalizes the total number of positions from each region. We also performed an additional analysis in which we tested for the possible influence of spatial effects by estimating SNP density using the same procedure, but with an additional requirement for nucleotides to be within 1 kbp of each other during the pairing. No spatial effects were detected, and so we omitted this control for our study.

### Simulation of SNPs from the study population

We measure the strength of influence of a single SNP (or set of SNPs from a genotype) by comparing the model solution for the reference genome to that for the case when the SNP (or SNPs) is superimposed onto the reference sequence. In this way, we define a ‘regulatory score’ *w* for a SNP (or a genotype) according to a specific measure of the difference between the two solutions. We use two scalar measures, the root mean square distance (*RMS*) and the weighted pattern generating potential (*wPGP*). *RMS* is the standard Euclidian distance, and the corresponding regulatory score for this measure is as follows:

$$\begin{array}{c} w=\sqrt{\frac{1}{N}\;\sum_{t,i,a}\;{\left(v_{i}^{a}\left(t\right)-V_{i}^{a}\left(t\right)\right)}^{2}}, \end{array}$$ (4)

where $v_{i}^{a}$ is the protein concentrations in the model solution for the mutated genotype, and $V_{i}^{a}$ is the same for the reference genotype (the wild type solution); the summation in Eq (4) takes place over all gap genes *a*, nuclei *i*, and times *t* for which the experimental data on the wild type gene expression is available, and *N* is the total number of the terms in the sum. We consider 9 time points during the blastoderm stage of *Drosophila* development: one time in the middle of cleavage cycle 13 (C13) and eight time classes T1–T8 in cleavage cycle 14A (C14A) [50, 51]. The total duration of the two cycles is 71 min (21 min for C13 and 50 min for C14A). Each time class in C14A is approximately 6 min long. We also consider 50 nuclei in C13, 100 nuclei in C14A, and four gap genes, all together leading to *N* = 3400.

*wPGP* is a heuristic measure first proposed in [5] and further developed in [40] and [32]. This measure captures characteristic features of spatial expression patterns more accurately than standard measures, such as *RMS* or correlation coefficient [5]. In particular, it is more sensitive to such changes in expression patterns as scaling, shift in basal expression, change of the length of expression domain, and partitioning of expression domain (see Figure 3B in ref. [5] for more details). The regulatory score for the *wPGP* measure is defined by the formula:

$$\begin{array}{c} w=\sum_{t,a}\;f^{a}\left(t\right),\;f^{a}=0.5-0.5*\left(\text{reward}-\text{penalty}\right), \end{array}$$ (5)

where, for given gene *a* and time *t*, the reward and penalty terms are calculated from the spatial expression pattern as follows:

$$\begin{array}{c} \text{reward}=\frac{\sum_{i}\;V_{i}^{a}\;\min\left(V_{i}^{a},v_{i}^{a}\right)}{\sum_{i}\;{\left(V_{i}^{a}\right)}^{2}},\;\text{penalty}=\frac{\sum_{i}\;\max\left(0,v_{i}^{a}-V_{i}^{a}\right)\left(V_{\max}^{a}-V_{i}^{a}\right)}{\sum_{i}\;{\left(V_{\max}^{a}-V_{i}^{a}\right)}^{2}}, \end{array}$$ (6)

where $v_{i}^{a}$ and $V_{i}^{a}$ are the model solutions already introduced in Formula (4), and $V_{\max}^{a}=V_{\max}^{a}\left(t\right)$ is the maximum value of the spatial expression pattern $V_{i}^{a}$ for a given time. The reward and penalty terms react differently to overexpression ($v_{i}^{a}>V_{i}^{a}$) and underexpression ($v_{i}^{a}<V_{i}^{a}$) in the mutated solution, and the equality $v_{i}^{a}=V_{i}^{a}$ leads to the minimal value of the score (*w* = 0; no influence from the SNPs). The terms ‘reward’ and ‘penalty’ stem from the optimization context, when there is a need to minimize the score, so the reward should be increased and penalty decreased in this case. Both *RMS* and *wPGP* scores are positive, and *f*^*a*^(*t*) ≤ 1 for the *wPGP* score.

The scores *w* quantify the effect of how strongly the spatio-temporal expression pattern in the model solution for the mutated genotype deviates from the wild type solution, so that SNPs with stronger influence on gene expression lead to larger *w* values. The *RMS* score is not sensitive to the direction of solution change (either $v_{i}^{a}<V_{i}^{a}$ or $v_{i}^{a}>V_{i}^{a}$), while the *wPGP* score does account for it, but in a heuristic and non-symmetrical way. In order to take the directional change into consideration, we additionally calculate a signed score **Δv** as a vector of differences at each point between the mutated and the wild type expression patterns in the model: $\Delta v=\{\Delta v_{i}^{a}\left(t\right)\}$, $\Delta v_{i}^{a}\left(t\right)=v_{i}^{a}\left(t\right)-V_{i}^{a}\left(t\right)$, where the indices and time run over the same values as for the scores Eqs (4)–(6). The difference vector has *N* components, either positive or negative, thus representing a signed and multidimensional measure for the SNP influence strength. We also consider similar scores **ΔE** defined as the difference between the transcriptional activation levels *E* from Eq (3) for the same values of *i*, *a*, and *t*.

### Simulation of random mutations

To detect any possible effects of purifying selection on segregating SNPs, we compare properties of mutations observed in the study population data to those we might have observed in data in which mutations were introduced randomly. If we see differences between the study population and datasets containing randomly introduced mutations, this provides some evidence of functional constraints. For this purpose we simulate multiple versions of the regulatory sequence for the four gap genes, each containing randomly mutated model TFBSs. We then run our model on these data.

**Fig 1:**
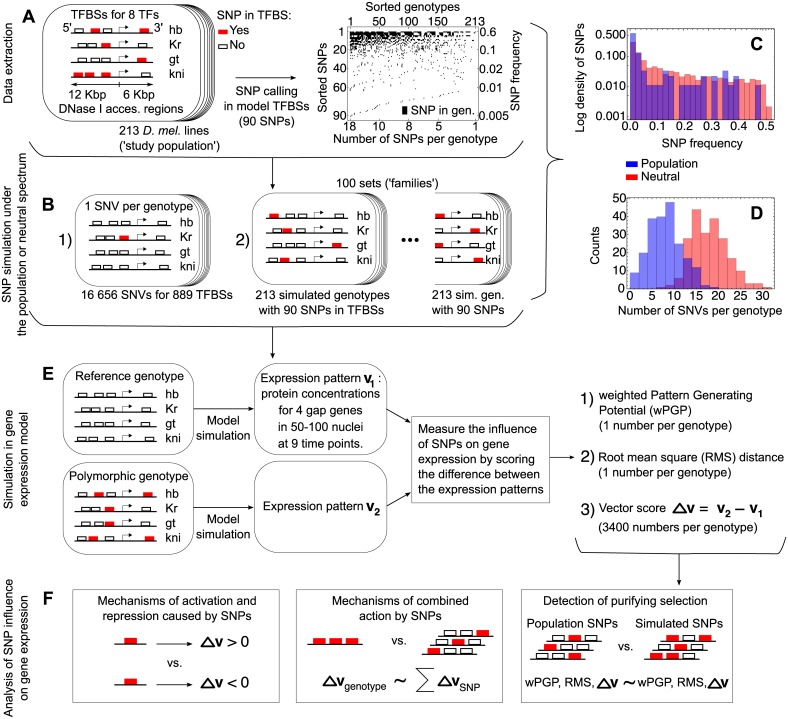
Summary of the approach. (A) Extraction of 90 SNPs within 889 model TFBSs of four gap genes from 213 polymorphic individual *D. mel* genotypes. The polymorphic TFBSs are marked by red. The SNP table is represented graphically, with a black box indicating that a given SNP is present in a given genotype. (B) Simulation of random mutations in the model TFBSs includes generating each possible SNV, or sets of SNVs randomly generated under either the neutral or population-derived site frequency spectrum (SFS). (C) The SNP frequency distributions derived from the study population (blue; the population-derived spectrum) and from the short intron sequences of the DGRP data (red; the neutral spectrum). (D) The distributions of the number of SNVs per genotype resulted from SNPs in the study population (blue) and in one family of randomly mutated genotypes. The family contains a total of 90 SNPs within the model TFBSs simulated under the neutral frequency spectrum (from C). The mean number of SNVs per genotype across 100 families is 17 ± 5. (E) The study population and randomly mutated genotypes were evaluated in the model of gap gene expression. The deviation of their expression patterns from the patterns for the reference genotype, estimated by the three scores, provides the measure of SNP influence on gene expression. (F) Three main directions for the analysis of SNP influence on gene expression.

The analysis of the model TFBSs in the population genotypes reveals 90 SNPs (Fig 1A). We perform three mutation experiments simulating random SNPs within the model TFBSs (Fig 1B). In the first, we introduce one point mutation per sequence. Repeating this for all possible single nucleotide variants (SNVs) (5552 positions in the model TFBSs multiplied by the three alternative nucleotides at each position), we obtain a total of 16656 sequences.

In the second experiment, we generate multiple random point mutations for each sequence. We simulate 100 sets (‘families’) of 213 randomly mutated sequences, considered as analogs of the natural population (213 genotypes from the data). For each family, we keep the total number of SNPs within the model TFBSs equal to this number observed in the model TFBSs of the study population genotypes (90 SNPs) and simulate each such SNP according to a ‘neutral’ frequency spectrum (Fig 1C, red). We define the neutral spectrum as the frequency distribution of SNPs extracted from short intron positions of the *D. melanogaster* genome, according to the DGRP data [52, 53]. The short intron positions provide the most neutral sequences across the *D. mel* genome [54, 55]. The short intron data were processed as described in ref. [49]. In particular, we selected only introns less than 86bp (the median length of introns in the *D. mel* genome is approximately 85bp), masked any short intron position which overlapped with UTR and CDS sequences, and masked out 8 base pairs on the intron edges (first and last 8 bases are highly conserved, probably for splicing). The resulting data set comprised 58041 polymorphic positions for 184 genotypes, and the site frequency spectrum (SFS) for this set was calculated and defined as the neutral spectrum (Fig 1C, red).

In the third mutation experiment, we simulate 100 families similarly to the second experiment except that each SNP is generated according to the spectrum derived from the 90 SNPs found in the model TFBSs of the study population (Fig 1C, blue). More precisely, each simulated genotype has exactly the same total number of mutations as some genotype from the study population, but the positions of these mutations are randomly chosen within the model TFBSs. For each mutated sequence from the three mutation experiments, we apply our model and calculate the same three regulatory scores as for the SNPs from the study population, according to Formulas (4)–(6) (Fig 1E).

## Results

We study polymorphism observed in regulatory regions of the gap genes *hb*, *Kr*, *gt*, and *kni* in the study population of 213 *Drosophila* lines (individual genotypes), in the context of our model of gene expression. Fig 1 presents a summary of our approach. We analyze 889 TFBSs (the model TFBSs) for the transcription factors Hb, Kr, Gt, Kni, Bcd, Cad, Tll, and Hkb. The set of experimentally observed SNPs in these TFBSs consists of a total of 90 SNPs at 139 unique bindings sites (Fig 1A; S2 Table). 34 of these SNPs fall into multiple TFBSs due to overlap, resulting in a total of 149 observed combinations of SNP and TFBS (S2 Table).

We also simulate genotypes containing various point mutations in the model TFBSs (Fig 1B), in order to use these genotypes for comparison with the study population. One set of such genotypes includes all possible single nucleotide substitutions within the model TFBSs, one SNV per genotype. We further use these genotypes to study the influence of a single point mutation on gene expression. Simulated genotypes from other sets contain multiple SNVs, generated using either the neutral site frequency spectrum or the spectrum observed in the population (Fig 1C). These sets, or families, serve as alternatives of the study population, since each family also comprise 90 SNPs but these polymorphisms are randomly distributed over genotypes and TFBSs. The use of two different spectra to generate SNPs in the families leads to different resulting distributions of the number of SNVs per genotype (Fig 1D). Given a genotype, either from the study population or from the simulated sets, we run our gene expression model on it and get three scores quantifying the perturbation that the polymorphism present in the genotype exerts on the gap gene expression patterns (Fig 1E). Finally, we analyze various aspects of SNP influence on gene expression using the scores (Fig 1F).

**Fig 2:**
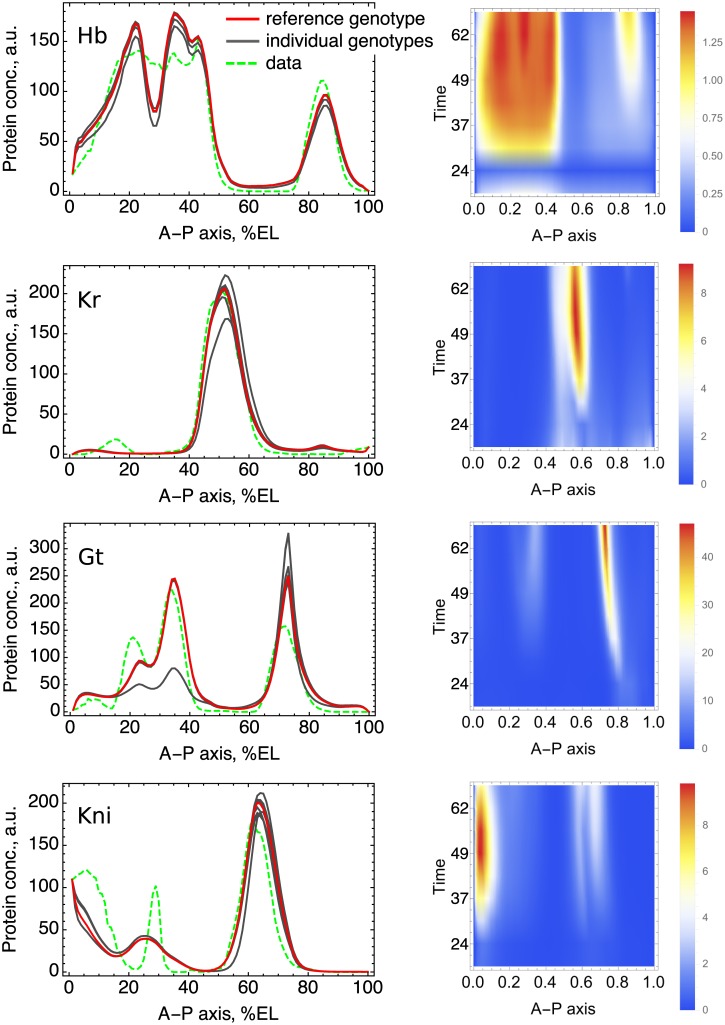
Variation of expression in the study population. The left column of panels shows expression patterns (model solutions) at the end of cleavage cycle 14A (time class T8; see Methods) for 213 individual genotypes and for the reference genotype, together with the observed expression patterns. Many gray curves group together in various spatial domains because the variation is small relative to the maximal expression level, as a consequence the curves coalesce in these parts of the figure. The same figures for all nine time points are shown in S1 Fig. The right column of panels represents the heat map for the population average of the expression difference **Δv**, for the same genes as for the panels on the left. The spatial coordinate represents the percent of the embryo length, and the time is in minutes from the start of the cleavage cycle 13.

Applying the model to the individual genotypes results in relatively little variation in expression patterns (Fig 2). The variation exists in all expression domains and spreads in time more or less homogeneously. In what follows, we analyze what determines the sign of SNP influence, investigate how combination of SNPs in the genotypes manifests at the expression level, and demonstrate evidence of purifying selection in the study population.

### SNPs activate (repress) expression via repressing (activating) TFBSs

When the presence of a SNP leads to increased expression comparing to the reference case, we can say that the SNP activates expression, and similarly we can define repression by a SNP. In the following two sections, we clarify the mechanisms of activation and repression caused by SNPs observed in the study population. We quantify the effect exerted on gap gene expression by a SNP by simulating the point mutation associated with this SNP in the regulatory regions of the gap genes and evaluating such genotype in the model (Fig 1B and 1E). We define a sign of the influence that a SNP exerts on expression via the combination of signs possessed by the components of the pattern difference vector **Δv** calculated for this SNP: the SNP has purely positive influence on expression (activation) if $\Delta v_{i}^{a}\left(t\right)\geq 0$ for all nuclei *i* and times *t*, and for gene *a* whose regulatory region contains this SNP, i.e. expression only increases in some or all spatial and temporal points when the SNP is inserted into the reference sequence; the SNP has purely negative influence (repression) if $\Delta v_{i}^{a}\left(t\right)\leq 0$ for all nuclei and times; the SNP has alternating sign if $\Delta v_{i}^{a}\left(t\right)$ is positive for some *i* and *t* and negative for others; and the SNP influence is evidently zero if $\Delta v_{i}^{a}\left(t\right)=0$ for all *i* and *t*. SNPs from the study population distribute almost symmetrically between the pure-activation and pure-repression groups: there are 36 SNPs with purely positive influence, and 38 SNPs with purely negative. Among the rest of the SNPs, 13 have alternating sign and 3 exhibit no influence.

**Fig 3:**
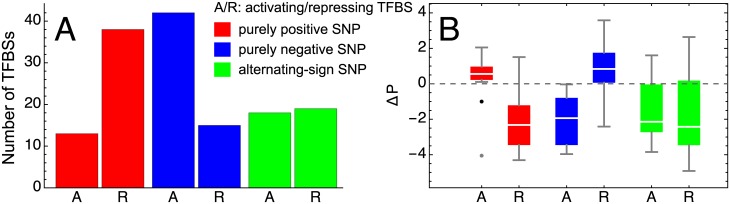
Positive and negative influence of SNPs from the study population. (A) Number of activating (‘A’) and repressing (‘R’) TFBSs containing SNPs with purely positive influence on gene expression (red), purely negative influence (blue), and with alternating sign (green). (B) Distribution of Δ*P* values for activating and repressing TFBSs and for different signs of SNP influence on expression. The labels and colors have the same meaning as in A. The dots show outliers.

We further analyze how the SNPs are distributed among the activating and repressing TFBSs and how this distribution relates to the SNP influence sign. Comparing the numbers of activating or repressing TFBSs containing SNPs from each of the influence sign groups, we find that it is more probable that a SNP with purely positive influence is located within a repressing binding site than within an activating site (*p* = 0.001 by a resampling test; S2 Fig), and the reverse is true for a SNP with purely negative influence on gene expression (*p* = 0.001; S2 Fig; Fig 3A). This means that activation from mutation occurs via alteration of repressing sites, while repression occurs via activating sites.

In order to explain this asymmetry, we first note that a SNP from a TFBS *s* exerts its effect in the model by changing the PWM score *P*_*s*_ of this site and, as a consequence, the statistical weight *q*_*s*_ (see Methods). This perturbation translates further to other levels in the model according to Eqs (1)–(3). We can estimate the difference between the binding probabilities of the mutated and reference states of each polymorphic TFBS by calculating the ratio of their statistical weights: $\log q_{s}/q_{s}^{\text{ref}}=P_{s}-P_{s}^{\text{ref}}=\Delta P$, where Δ*P* represents the corresponding difference in the PWM scores. The distribution of Δ*P* reveals a bias towards negative values (S3 Fig). The existence of this bias is expected and stems from the fact that TFBSs stand out against the genetic background by having larger values of the PWM score, representing better affinities of these DNA segments to specific TFs as compared to a random segment. Therefore, it is more likely that a mutation in a TFBS would decrease the affinity of the site, shifting its sequence closer to the background sequence, and this would lead to a negative value of Δ*P*.

Given that reduction of the TFBS affinity is the most likely output from a SNP appearance, it is clear that activation effects on gene expression should most likely come from mutating TFBSs for repressors, since reducing the probability for a repressor to bind would reduce its repressing action on expression and, hence, lead to effective activation. Similarly, SNPs affecting activating TFBSs lead to effective repressive action. In accordance with this logic, we see qualitative difference in the distributions for Δ*P* analyzed separately for activating and repressing TFBSs and for each group of SNPs associated with a specific sign of influence on expression (Fig 3B). For example, Δ*P* is mostly positive for activating TFBSs and negative for repressing TFBSs if these sites contain SNPs with purely positive action on expression, and the reverse is true if these sites contain purely repressing SNPs. However, we note that the presented analysis does not take into account novel TFBSs that may appear due to mutations. An analysis including this effect may change the patterns in Fig 3.

### Sign of SNP influence depends on local vicinity of this SNP in the sequence

The SNPs with alternating sign of influence on expression show no preference in choosing between an activating or repressing TFBS (Fig 3A) and tend to decrease the binding site affinity irrespective of the type of the site (Fig 3B). We can describe the mechanisms of how a SNP from a TFBS *s* can be activating in some conditions and repressing in other conditions by analyzing the derivative ∂*E*/∂*q*_*s*_, where *E* is the probability of transcriptional activation from Eq (3). The SNP can either increase or decrease the statistical weight *q*_*s*_ of the site, so, if the derivative has a definite sign for all parameters in the model, then the SNP will have a definite sign for its influence on expression. We note though that this analysis is true only if there are no indirect interactions (via TFs) between SNPs, or if such interactions are negligible.

Calculations reveal that the SNP influence may change its sign as a result of local interactions between the polymorphic site and other sites in the vicinity (see the calculation details in S1 Text). We say that two TFBSs locally interact with each other if the binding probability of one TFBS depends on a state of the other TFBS. Such interactions appear as a result of overlaps between TFBSs, due to cooperative binding, or if a TFBS occurs within the repression range of a repressing TFBS. If a polymorphic TFBS is independent of its neighbourhood, i.e. it does not participate in any such interactions with other sites, then SNPs at this site either always activate or always repress expression.

**Fig 4:**
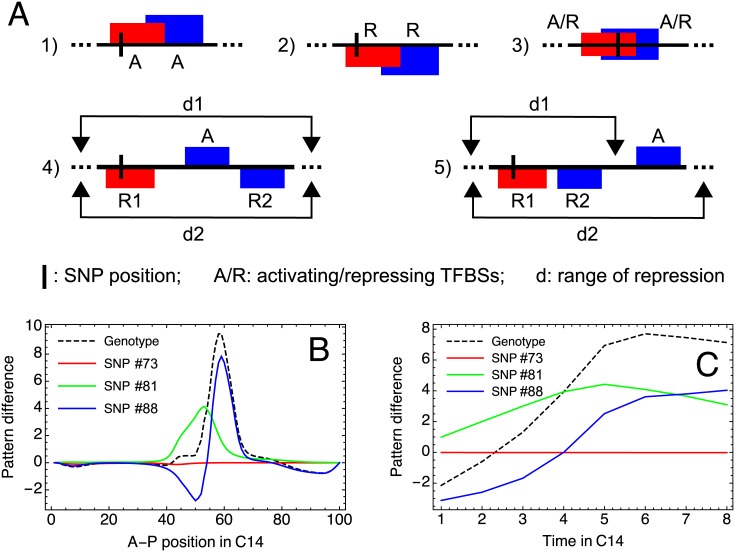
Mechanisms for SNP influence sign alterations. (A) Examples of local interactions of a polymorphic TFBS (red box) with other TFBSs (blue boxes) leading to alternating sign of SNP influence on expression. The horizontal lines represent segments of the regulatory region. Activating (repressing) TFBSs are shown above (below) these lines and labeled with ‘A’ (‘R’). The SNP position is marked via a short vertical line. In 3), the SNP appears in the overlap region of two TFBSs, so both sites are polymorphic in this picture. They are shown at the center of the DNA line to express that both sites can be either activating or repressing (in any combination) in this situation. In 4) and 5), *d*1 and *d*2 indicate the range of repression from sites R1 and R2, respectively. The activating site is repressed by both repressors in 4) and only by R2 in 5). (B) Graphs of the difference $\Delta v_{i}^{a}$ as a function of the spatial position *i* on the A–P axis of the embryo in the mid-cleavage cycle 14A (time class T4) and for the gene *Kr*. The graphs are shown for one genotype from the study population and its three SNPs from the regulatory region of *Kr*. The indices of these SNPs (#73, 81, and 88) are according to S2 Table. SNP #73 has the genomic position 21106823 and corresponds to the change of nucleotide G to A; SNP #81, 21113108, T/G; SNP #88, 21113686, C/T. Each SNP appears in multiple overlapping TFBSs of multiple TFs (S2 Table). (C) Dynamics of $\Delta v_{i}^{a}$ for the same genotype, gene, and SNPs as in (B), shown for time classes T1–T8 in the cleavage cycle 14A and for nucleus *i* = 54. The blue curve in (B) and (C) demonstrates the alternating sign of SNP influence.

Fig 4A shows some configurations in the vicinity of a polymorphic site which may lead to influence of alternating sign. The first two cases in Fig 4A correspond to the polymorphic site overlapping with another site of the same type (either activating or repressing). Explaining case 1 in the figure in more detail, suppose that the SNP increases the binding affinity of the polymorphic activating site, leading to a higher probability of binding for the corresponding activating TF and, consequently, to higher activation. On the other hand, such a SNP will reduce the probability of binding for the overlapping activating site, since the overlap forbids simultaneous binding to both sites, and this action represents an implicit repressive action of the SNP. The sign of the net influence of the SNP thus depends on the balance between these two actions, which in turn depends on the relative importance of the sites for activating the target gene and on the concentrations of corresponding TFs. Therefore, variation of TF concentrations in time and space may shift this balance to activation of expression at some spatial and temporal points and to repression at other points. Case 2 in Fig 4A can be analyzed in a similar way. These two configurations demonstrate that the sign change occurs because the polymorphic TFBS and the other depicted TFBS compete for influence of the same type (either activation or repression).

The SNP influence may change sign also when the SNP occurs in the overlapping region (case 3 in Fig 4A). As the SNP appears in two TFBSs simultaneously, the sign of the second derivative ∂^2^*E*/(∂*q*_1_∂*q*_2_) w.r.t. the statistical weights *q*_1_ and *q*_2_ of these sites should be analyzed in this case to prove the possibility for sign alteration (S1 Text). To find out which mechanism of sign alteration prevails in the study population, we compare the events of two types (Fig 4A, case 1 or 2, and Fig 4A, case 3), and compare the total numbers of such events observed for the population SNPs and for multiple positions randomly sampled from the model TFBSs (S4 Fig). All 13 sign alternating SNPs from the population appear in the overlapping region of multiple TFBSs, and the sampling does not produce the same number (*p* < 0.001). On the other hand, the overlaps from the cases 1 and 2 in which the sign alternating SNPs participate are as numerous as expected by chance (*p* = 0.577). Therefore, we conclude that the sign alteration mechanism from the case 3 is a distinctive feature of the population.

Another type of interaction associated with sign alteration concerns competition for the repressive function exerted on site-activators (cases 4–5 in Fig 4A). According to the short-range repression mechanism, a TFBS-repressor affects the target gene indirectly by reducing occupancy of activating sites within the repression range. When an activating TFBS appears in the repression ranges of two repressing sites, the cumulative repression effect on the activator is balanced by these two repressors. A SNP in one of these repressing sites may perturb this balance, shifting to less repression for some TF concentrations and to more repression for other concentrations. Calculations show that this may happen even when the activating site is not within the repression range of the polymorphic TFBS; it is enough for the polymorphic site to occur inside the repression range of a mediator in the form of another repressing site with direct influence on activating sites (case 5 in Fig 4A; S1 Text). Various combinations of local configurations from Fig 4A theoretically may lead to long interaction chains for a polymorphic TFBS, resulting in potentially complicated mechanisms of SNP influence on gene expression.

Fig 4B and 4C demonstrate that the sign alternating SNPs from the study population lead to both spatial and temporal variation in the influence sign. This figure presents specific examples of three SNPs in one individual genotype affecting *Kr* expression. It also shows different combination types for SNP influences, as the curve for the genotype in Fig 4B represents the sum of the curves for three SNPs at each spatial position. There can be almost exclusive control by a single SNP or aggregation of the influence of several SNPs, and they can compensate each other when they have different signs.

### Influences from multiple SNPs combine in a non-linear and non-local way under the thermodynamic approach

We now use the analytical predictions of our model to explore how combinations of SNPs jointly influence gene expression. The key component of the model equations (Eqs (1) and (2) in Methods) is the transcriptional activation level *E* (Eq (3) in Methods). The model allows for all possible molecular configurations of the regulatory sequence (combinations of free and bound TFBSs) and captures the influence of these configurations on the BTM. The binding state of a TFBS *S* is taken into account via the weight *q*(*S*) (we use this notation instead of the notation *q*_*s*_ used in Methods). A SNP at the *j*th position within *S* changes its binding affinity by replacing a nucleotide at that position and, as a consequence, affects the weight of the site. We quantify the change in binding affinity by calculating a quantity *δ* representing a scaled difference between normalized frequencies of the original and new nucleotides at the *j*th position (see S1 Text for details). Therefore, *δ* can be viewed as the first manifestation of the SNP in the model equations. The weight of the mutated version $\tilde{S}$ of the site depends linearly on *δ* as follows:

$$\begin{array}{c} q\left(\tilde{S}\right)=q\left(S\right)+q\left(\bar{S}\right)\delta , \end{array}$$ (7)

where $\bar{S}$ is the reduced form of site *S* obtained by removing the *j*th position, and the weight of site $\bar{S}$ is calculated by using the same PWM matrix as for site *S* but with *j*th column removed. Let us now consider a genotype containing *n* SNPs in binding sites *S*_1_, *S*_2_, …, *S*_*n*_ (one SNP per binding site) with corresponding perturbations *δ*_1_, *δ*_2_, …, *δ*_*n*_. The activation level $\tilde{E}$ for this genotype has the form:

$$\begin{array}{c} \tilde{E}=\frac{Z_{ON}+P_{n}\left(\delta_{1},\ldots ,\delta_{n}\right)}{Z_{ON}+Z_{OFF}+Q_{n}\left(\delta_{1},\ldots ,\delta_{n}\right)}, \end{array}$$ (8)

where *Z*_*ON*_ and *Z*_*OFF*_ are the relative probabilities of the states of the original (reference) genotype with the BTM bound and unbound, respectively, constituting the activation level *E* from Eq (3); *P*_*n*_ and *Q*_*n*_ are polynomial functions of their arguments which contain all possible products of *δ*_*i*_, with coefficients depending only on sites *S*_*i*_ and their reduced versions ${\bar{S}}_{i}$ (full details of the expressions are given in S1 Text).

The Formula (8) implies that the transcriptional activation in the model depends on the perturbations *δ*_*i*_ from the SNPs in a non-linear way. This expression also reveals that the magnitude of the effect that a set of SNPs has on the transcriptional activation is due not only to the TFBSs that physically interact with the mutated sites (for example, via cooperative effects or overlapping), but also a consequence of all binding sites in the regulatory sequence. This occurs because the thermodynamic approach describes a quasi-equilibrium situation, so that all sites participate in balancing the relative probabilities for site *S*_*i*_ being free or bound. As a consequence, changes to these binding probabilities are modulated by the weights of other binding sites. Therefore, SNPs have non-local effects in the thermodynamic model of gene expression. In other words, the effect of one SNP on gene expression is conditional on the effects of all other SNPs in the model.

### Combinations of SNPs from the study population exhibit increased levels of additivity of regulatory effects

Following the analytical predictions, we apply the model to simulate separate SNPs and individual genotypes from the study population. We aim to quantify the level of additivity in the influence of SNPs on expression. For each of the 90 SNPs from the study population, we simulate the corresponding SNV in the reference genotype, evaluate such genotype in the expression model, and calculate the score **Δv**_SNP_ measuring the perturbation which the selected SNP exerts on expression patterns (Fig 1E). Simulating a set of SNPs observed in each of the 213 population genotypes, we obtain the similar measure **Δv**_gen_, which we call the score for the genotype. Our population genotypes include from 0 to 18 SNPs per genotype within the model TFBSs, with a median of 7 SNPs (Fig 1D). We analyze the additivity by comparing the overall score **Δv**_gen_ for a genotype to the quantity ∑_SNP_ **Δv**_SNP_, where the sum is taken over all SNPs seen in this genotype. In what follows, we simplify the notation for the components $\Delta v_{i}^{a}\left(t\right)$ of the vector **Δv** by using a unified index *k* for the set of values {*i*, *a*, *t*}, so that we write $\Delta v_{\text{gen}}^{k}$ instead of $\Delta v_{i}^{a}\left(t\right)$ for the components of the genotype score **Δv**_gen_, and similarly for the SNP scores.

Exploring the genotype scores $\Delta v_{\text{gen}}^{k}$ for all 213 genotypes from the study population and all values of *k*, we see that 36% of all values correspond to cases in which two or more SNPs with non-vanishing influence combine in the regulatory region of the same gene *a*. We collect all pairs $\left(\sum_{\text{SNP}}\Delta v_{\text{SNP}}^{k},\Delta v_{\text{gen}}^{k}\right)$ for these cases of multi-SNP influence, where the sum is taken over the SNPs in the corresponding genotype. We next split this data in two sets according to the level of prevalence of the strongest SNP in a given genotype. The first set consists of pairs corresponding to cases for which the difference between the largest score $\Delta v_{\text{SNP}}^{k}$ for SNPs in the genotype, and the sum of the scores for the rest of SNPs in this genotype, is less than 70% of the largest score. In other words, the pairs $\left(\sum_{\text{SNP}}\Delta v_{\text{SNP}}^{k},\Delta v_{\text{gen}}^{k}\right)$ from this set correspond to the cases in which the score for the strongest SNP in the genotype is not very different from that for other SNPs in this genotype. Conversely, SNPs in the second set correspond to cases in which the score for the strongest SNPs differs more greatly from scores for other SNPs in the genotype.

**Fig 5:**
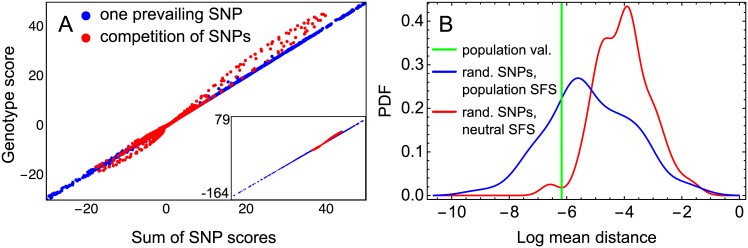
Combination of SNP influences for the 213 individual genotypes. (A) The scatterplot for the genotype score $\Delta v_{\text{gen}}^{k}$ vs. the sum $\sum_{\text{SNP}}\Delta v_{\text{SNP}}^{k}$ of the SNP scores over all SNPs from the genotype, for all genotypes and values of *k* corresponding to contribution from two or more SNPs from the same regulatory region. The blue color labels points with a prevailing influence from only one SNP within a genotype, and the red color corresponds to the competition from multiple SNPs, as described in the text. The threshold value (70% of the largest SNP score) chosen to discriminate the blue and red points was manually tuned to make the red points comprise all points outside the additivity line (S5 Fig shows the deviation from this line as a function of this threshold). The panel shows the magnified graph for better visualisation, and the inset displays the full range. (B) Measure of SNP influence additivity for the population (green line) in comparison with the same measure for families of genotypes randomly simulated under the neutral SFS (red) and the population-derived SFS (blue). The measure is the mean distance from the points $\left(x,y\right)=\left(\sum_{\text{SNP}}\Delta v_{\text{SNP}}^{k},\Delta v_{\text{gen}}^{k}\right)$ to the line of pure additivity *y* = *x*, as explained in the text. The distances were calculated for all *k* values related to a genotype (1 ≤ *k* ≤ 3400) and for 213 polymorphic genotypes from the population or from a family of randomly simulated genotypes, and an average value was obtained for the population and for each family. For each SFS type, the probability density function (PDF) was estimated from the mean distance distribution across 100 families of randomly simulated genotypes.

Visualization of this data demonstrates an essentially linear dependence between $\Delta v_{\text{gen}}^{k}$ and $\sum_{\text{SNP}}\Delta v_{\text{SNP}}^{k}$ (Fig 5A). This means that, in order to predict the variation in the expression pattern caused by the combined action of a set of SNPs, it is enough to sum up the variations that would be caused by each SNP separately. In other words, the effects of SNPs observed in the population are mostly additive. On the other hand, this additivity in the effects of SNPs appears mostly for genotypes in which one strong SNP contributes the greatest part of the variation in the expression pattern for this genotype (blue points in Fig 5A, corresponding to the second set described above). The non-linear portions of the scatter plot in the figure (red points, corresponding to the first set) are solely due to the non-additive combination of several SNPs in a genotype (S5 Fig), in agreement with the analytical results.

To test whether this level of additivity is specific to the study population genotypes, or rather a direct consequence of the model, we compare the relation between **Δv**_gen_ and ∑_SNP_ **Δv**_SNP_ observed in the population with that obtained for randomly simulated SNPs. The effects of SNPs are purely additive when **Δv**_gen_ = ∑_SNP_ **Δv**_SNP_. Plotting values $\sum_{\text{SNP}}\Delta v_{\text{SNP}}^{k}$ on the *x*-axis and $\Delta v_{\text{gen}}^{k}$ on the *y*-axis of the (*x*, *y*) plane (as in Fig 5A), pure additivity corresponds to the case when all these values appear on the line *x* = *y*. One measure for additivity is a distance from the points $\left(\sum_{\text{SNP}}\Delta v_{\text{SNP}}^{k},\Delta v_{\text{gen}}^{k}\right)$ to this line, and larger distances correspond to larger deviation from the additivity of SNP effects. We compute this distance for all values of *k*, related to a genotype, and for all genotypes from the study population and from the sets of random polymorphic genotypes simulated under two different SFS. Each set (‘family’) contains 213 randomly mutated genotypes, and we consider 100 such families for each SFS type. The mean distance for the study population is decreased compared to the genotypes randomly mutated under the neutral SFS (*p* = 0.02) and not distinguishable from the distances for the genotypes randomly mutated under the population-derived SFS (*p* = 0.24; Fig 5B). This result implies that the composite effects of the naturally occurring polymorphisms exhibit a higher level of additivity than expected from the conditions of neutral mutations.

### Variation of SNP influence is approximately the same on the levels of transcriptional activation and gene products

As the model links the transcriptional activation level with that of protein concentrations, we can compare variation of SNP influence on these two levels. This problem is partially related to the general question regarding the coexistence of the stochastic nature of transcription activation with the low level of variation of the gap gene products, which was observed experimentally and attributed to the action of the spatio-temporal averaging [56]. Even though our model is not stochastic, it contains information about the state of the target genes in the form of the transcriptional activation probability *E*, which can be viewed as an average of gene stochastic state. We measure influence of SNP combinations at the level of transcriptional activation with the help of scores **ΔE** calculated for each genotype from the study population similarly to the scores **Δv** for protein concentrations. We aim to explore whether the influence of SNPs becomes less variable when transitioning from the level of transcriptional activation to the level of protein concentrations.

**Fig 6:**
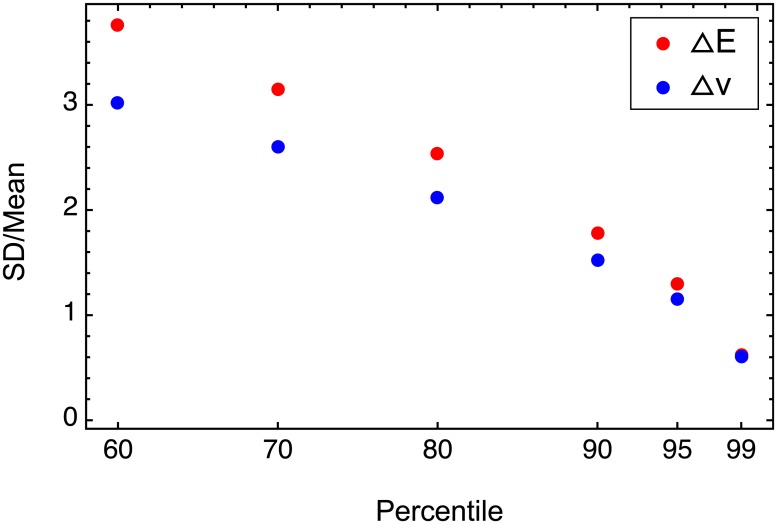
Variation of SNP influence on the levels of transcriptional activation and gene products. Coefficient of variation (the standard deviation to the mean ratio) for different subsets of the set of absolute values $|\Delta v_{\text{gen}}^{k}|$ (blue) and $|\Delta E_{\text{gen}}^{k}|$ (red) for all genotypes from the study population and all *k* values. The coefficient was calculated for values of $|\Delta v_{\text{gen}}^{k}|$ or $|\Delta E_{\text{gen}}^{k}|$ exceeding the percentile values shown on the horizontal axis.

As *E* is a continuous function of TF concentrations and other parameters, for a given genotype the spatio-temporal distribution of the components Δ*E*^*k*^ is the result of the spatio-temporal distribution for TF concentrations ‘filtered’ through all possible configurations of the regulatory regions for each nucleus and time-point, according to Eq (3). The protein concentrations are the dynamical solution of the reaction-diffusion equations, embracing *E* as the reaction term, so we can consider the spatio-temporal distribution of the components Δ*v*^*k*^ as the result of the spatio-temporal averaging (according to the model) controlled by the distribution of Δ*E*^*k*^. Changes of genotype lead to variation of **ΔE**, which then translates to variation of **Δv**. We calculate **ΔE** and **Δv** for all genotypes and compare the joint distribution of absolute values |Δ*E*^*k*^| for all genotypes and all values of *k* with the same joint distribution of |Δ*v*^*k*^| (S6 Fig). The coefficient of variation does not differ significantly for the two quantities, and the difference diminishes for SNPs with larger influence (Fig 6). Therefore, the influence of SNPs from the study population exhibits comparable variability on the levels of transcriptional activation and protein concentrations, as assessed by the thermodynamic model.

### TFBSs selected for the model exhibit reduced polymorphism

In what follows, we analyze possible signs of purifying selection in the data and aim to answer the following question: Do the properties and effects of polymorphisms observed in our natural population resemble those of random mutations, or have they additionally been shaped by selection? Before answering this question, we first test that the model includes a set of TFBSs appropriate to study purifying selection. TFBSs with a regulatory function should presumably be more conserved than other non-coding DNA regions [47]. We test for this property by comparing the distributions of the number of SNPs in the model binding sites to that in other parts of the regulatory regions of the four gap genes. To compare the levels of polymorphisms while controlling for confounding factors (GC content, sample length, and possible spatial effects), we apply the bootstrap method described in Methods.

**Fig 7:**
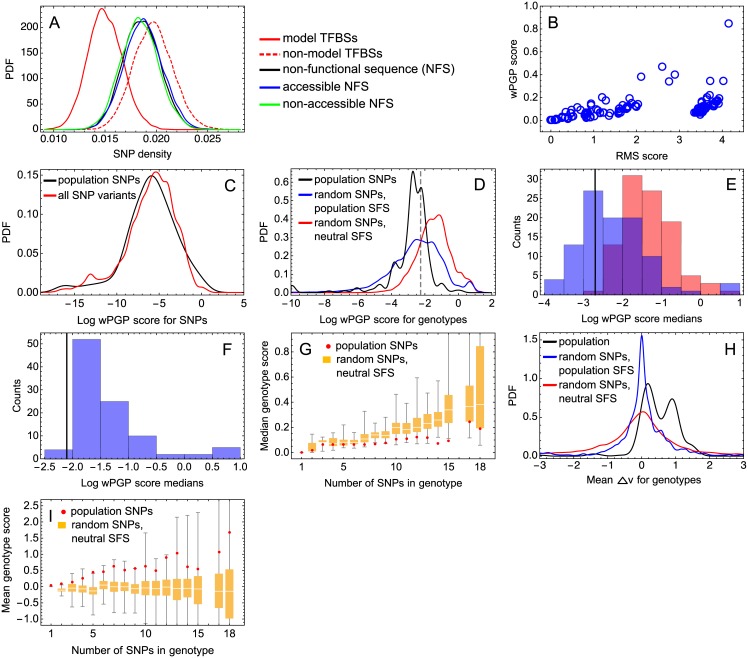
Signs of purifying selection acting on the gap gene network. (A) Comparison of SNP densities in the model TFBSs and in other parts of the regulatory sequence. The probability density function (PDF) for each genetic region is estimated from the joint sample obtained by the 20-fold resampling of 170 strains in the SNP data and by the 500-fold bootstrap procedure for the position sets chosen to control for GC content and equal length of the samples. The PDF is shown for the TFBSs from the model (red solid curve), putative TFBSs not included in the model (red dashed curve), the non-functional sequence (black) and its accessible (blue) and non-accessible (green) components (see Methods for definition of the regions). All distributions exhibit statistically significant mutual difference (*p* < 0.0001, according to the Mann-Whitney and sign tests, and to the bootstrap KS test). (B) Correlation between the *RMS* score from Eq (4) and the *wPGP* score from Eqs (5) and (6), for all genotypes from the study population. For better visualisation, an outlier with coordinates (10.7, 1.2) is not shown. (C) The distribution of the log-transformed *wPGP* regulatory scores for the 90 SNPs observed in the study population (black), and the same distribution for all possible SNP variants simulated in the model TFBSs (red). (D) The log-transformed *wPGP* score distributions for the 213 polymorphic genotypes from our study population (black), for 100 families of 213 artificial genotypes (pooled together) simulated under the neutral SFS (red), and for 100 families simulated under the population-derived SFS (blue). The dashed line marks the 2/3-quantile of the population score distribution, used for analysis in panel F. (E) The distributions of the family median of *wPGP* score for two types of families. The black line marks the population median. The colors are as in D. (F) The same as in panel E, but with medians calculated only for genotypes of strong effect on expression, i.e. genotypes with the score exceeding the value marked by the dashed line in panel D. Results for families with the neutral SFS are omitted. (G) The median *wPGP* score for genotypes vs. the number of SNPs in the genotype, for the observed genotypes (red points) and for genotypes simulated under the neutral SFS (box plots). Genotypes are grouped according to the number of SNPs they contain, and median scores are calculated for each such group. For the simulated genotypes, sampled values of the median are shown as box plots. For each group, we conduct 1000-fold sampling (with replacement) of simulated genotypes with the same depth as the number of the data genotypes in the group and calculate the median for each sample. (H) The distributions of the mean **Δv** calculated for each genotype from the study population (black) and for all genotypes from families simulated under the neutral SFS (red) or the population-derived SFS (blue). The difference between each pair of the distributions is statistically significant based on the bootstrap KS test (*p* < 0.0001). (I) The same as in G, but with the genotype related mean 〈**Δv**〉 used instead of the *wPGP* score.

This analysis reveals that SNPs occur less frequently in the TFBSs selected for the model compared to the non-functional sequence and the non-model binding sites (Fig 7A). Based on a 20-fold sampling of 170 genotypes in the SNP data set, we find on average 704 ± 5 polymorphic positions in the non-functional sequence, 363 ± 5 in the non-model binding sites, and 84 ± 2 in the model binding sites. The bootstrap procedure results in very similar median SNP densities in the non-functional sequence (0.019 ± 0.002 polymorphic positions per nucleotide) and in the non-model binding sites (0.020 ± 0.002), although the difference between these medians is still statistically significant (*p* < 0.0001; Fig 7A). However, the median SNP density for the model binding sites is 0.015 ± 0.002, which is 19% smaller than for the non-functional sequence. The model binding sites are in accessible chromatin, while a part of the non-functional sequence is not. To account for any possible differences due to accessibility, we split the non-functional sequence into accessible and non-accessible parts and then use the same bootstrap algorithm. The results show that these components of the non-functional sequence exhibit statistically significant difference (*p* < 0.0001), but this difference cannot explain the difference between the non-functional sequence and the model TFBSs (Fig 7A). Overall, our analysis demonstrates a higher level of conservation for the bindings sites of TFs regulating the gap gene network during early development. The model binding sites appear to experience purifying selection.

### Binding affinities provide vague evidence for the action of purifying selection

A SNP influences gene expression by modifying the binding affinity of a TFBS, which means this affinity can be viewed as an intermediate phenotypic characteristic [57]. It is interesting to track the SNP influence on this intermediate level and compare results with those seen at the gene expression level. We aim to explore whether information solely about the binding affinities of TFBSs is enough to detect action of purifying selection, without involving sophisticated models of gene expression based on those affinities.

Considering a TFBS *S* which contains a given SNP, we calculate a ‘binding score’ *w*_*b*_ of this SNP by quantifying the change of the binding energy of this site due to the presence of the SNP:

$$\begin{array}{c} w_{b}=\left|P\left(S_{\text{mut}}\right)-P\left(S_{\text{ref}}\right)\right|,\;P\left(S\right)=\sum_{i=1}^{n}\;P\left(S^{i}\right), \end{array}$$ (9)

where *S*_ref_ and *S*_mut_ are the reference and mutant (i.e. containing the SNP) states of the site *S*, respectively; *P* is the log likelihood ratio score, or the PWM-score, of the site *S* quantifying the binding affinity of the site [30, 58] (S1 Text). If the site consists of *n* positions *S*^*i*^, its PWM-score *P*(*S*) is calculated as a sum of the inputs *P*(*S*^*i*^) from each position. We investigate how the change in binding affinities of the model TFBSs caused by SNPs in our natural population compares to that caused by randomly generated SNPs (16656 substitutions in total, see Methods). We find no statistically significant difference between the distribution of the binding scores *w*_*b*_ for SNPs from the study population versus those resulting from randomly generated mutations (*p* = 0.27 using the bootstrap chi-square test, and *p* = 0.09 comparing the population and sampled medians; S7 Fig). However, the comparison of the population mean of the score with the sampled means for the random mutations shows the difference (*p* = 0.003; S7 Fig). As the mean is more sensitive to outliers than the median, this discrepancy in predictions may indicate that at least SNPs observed in the population contain fewer mutations associated with very large perturbation of binding affinity of TFBSs than expected by chance.

A possible explanation for the fact that the binding affinities are not very sensitive to the action of purifying selection stems from a rather small correlation between the binding and regulatory scores of SNP influence (Spearman’s *ρ* = 0.22 with *p* = 0.003 based on the permutation test; the Pearson correlation coefficient on the log-transformed values is *r* = 0.24 with *p* = 0.001; see the scatter plot in S8 Fig). This result shows that the binding and gene expression levels appear relatively independent, i.e. a SNP with a moderate influence on binding affinity may cause no substantial effect on expression patterns.

### Individual SNPs do not show evidence of being under purifying selection

To further analyze the action of purifying selection on the gap gene network, we consider the expression profiles computed in the model as a ‘phenotype’ and contrast i) the variation of this phenotype that results from SNPs observed in the natural population, to ii) the phenotypic variation that is predicted to result from randomly generated mutations. We use the regulatory scores from Eqs (4)–(6) as our measure of the perturbation resulting from a SNP or a set of SNPs. Two different measures (*RMS* and *wPGP*) lead to different distributions for the regulatory scores of the individual genotypes (Fig 7B). The *RMS*-based distribution exhibits a clear clustering of expression response to the polymorphisms. This is caused by the influence of a few particularly influential SNPs present in the genotypes of a subset of the population (see S2 Text for more details on the SNPs that cause this clustering). This comparison illustrates how different measures may lead to distinct qualitative effects and adds to previous comparison results for the measures [5, 32]. As we discussed in Methods, the *wPGP* measure is better in the context of gap gene expression patterns, so we use only this measure thereafter.

First, we consider individual SNPs. The model predicts an almost exponential distribution for the SNP regulatory scores (the distribution of log-transformed scores is shown in Fig 7C). This form of distribution will result from having many relatively weak sites working in concert, with only a few essentially strong sites [34, 40]. If strong SNPs are typically deleterious, we would predict that they have low frequency of occurrence in the population compared to SNPs of weaker effect. However, the negative correlation we observe between SNP regulatory scores and the SNP frequency in the population, although suggestive, is not very significant (the rank correlation *ρ* = −0.15, *p* = 0.086 by the one-tailed permutation test; the Pearson correlation on the log-transformed data *r* = −0.20, *p* = 0.035 by the one-tailed permutation test; scatterplots are shown in S9 Fig).

We further explore this by simulating all possible single nucleotide substitutions in all model TFBSs and calculate the *wPGP* regulatory scores for each such artificial SNP. The distribution of the scores for SNPs observed in our natural population does not exhibit a significant difference from the distribution of scores that results from this simulation (Fig 7C; *p* = 0.740 using the bootstrap Kolmogorov–Smirnov (KS) test). In addition to the non-signed measure provided by the *wPGP* score, we analyze the signed scores for SNP influence provided by **Δv** and calculate the mean 〈**Δv**〉 of this score by averaging Δ*v*^*k*^ over all *k*. We calculate 〈**Δv**〉 for each unique SNP from the study population and each artificial SNP. This mean can be either negative or positive, representing a prevalence of either positive or negative perturbations in the expression pattern caused by a SNP. Similarly to the case with the *wPGP* score, the distribution of 〈**Δv**〉 for the population SNPs is statistically indistinguishable from that for the artificial SNPs (*p* = 0.765 based on the bootstrap KS test; S10 Fig). Thus, individually, SNPs appear to be under very weak, if any, selection. To reconcile the weak purifying selection with the significantly lower SNP density in TFBSs, we re-invoke the skewed distribution of SNP effects; where a majority of strong effects SNPs would likely be removed from population, while weak-effect segregating SNPs would be nearly neutral.

### Combinations of SNPs show evidence of being under purifying selection, leading to specific changes in the expression patterns

Next, we test for putative effects of combinations of SNPs. We simulate 200 families of polymorphic regulatory sequences with random SNP combinations and calculate the regulatory scores for each simulated genotype. Each family resembles the observed study population in the total number of SNPs, and the family SNPs are simulated under either the neutral or population-derived frequency spectrum (100 families for each spectrum type; see Methods). Comparing the scores for the study population and for these families, we test two different hypothesis. Families with the neutral SFS, on average, have more mutations per genotype than observed in the population (Fig 1D), while mutations in families with the population-derived SFS differ from those in the population only by their positions within TFBSs. If the population scores differ from the scores for families of both types, we interpret this difference as a detection of purifying selection affecting both the number of mutations in TFBSs and the positions of mutations within TFBSs. If the difference with the population occurs for families with the neutral SFS but not for families with the population SFS, this corresponds to purifying selection affecting the number of mutations but not their positions.

In contrast to the case for individual SNPs, the distribution of the scores for the genotypes in our study population does significantly differ from the joint distribution for all simulated genotypes, in the case of families simulated under the neutral SFS (Fig 7D). We statistically evaluate the difference between the distributions by comparing the median score for the population and for each family (Fig 7E). The medians for all families with the neutral SFS are larger than the median score for the study population (*p* < 0.01), while the difference for the case of the population-derived spectrum is not significant (*p* = 0.34). This suggests the model perceives purifying selection acting at the level of the total number of mutations in TFBSs, but not at the level of specific positions of these mutations, at least for the given natural variation extent and for the chosen measure of SNP influence.

Interestingly, we do not see the difference between the population and randomly mutated genotypes for both types of SFS in the case of the *RMS* score used instead of the *wPGP* score (S11 Fig; *p* = 0.29 and *p* = 0.70 for the neutral and population-derived SFS, respectively). This is another indication that the two measures can be associated with qualitatively different conclusions [5, 32, 40].

Comparing the score distributions for the population genotypes and for random mutations with the population-derived SFS in more details, we note that they have rather similar form for weak perturbations (the left tails of the black and blue curves in Fig 7D), but essentially differ from each other for strong perturbations (the right tails of these curves; the bootstrap KS test shows a vanishing *p*-value for the difference between the distributions). We have also shown above that the population genotypes are not distinguishable from randomly generated genotypes in terms of individual SNP influence. These facts suggest that only strong enough perturbations should be analyzed when discriminating between the population and random mutations, because either the model or selection itself may not be sensitive to SNPs and their combinations having a weak effect on expression. Using the 2/3-quantile of the population score distribution (the dashed line in Fig 7D) as a threshold separating genotypes with strong SNP combinations from those with weak SNP combinations, we see that the population median score for genotypes of strong effect is significantly smaller than similar medians for families with the population-derived SFS (Fig 7F; *p* = 0.01). This result provides evidence that the model is able to detect purifying selection affecting not only the total number of mutations per genotype, but also their positions within TFBSs, if mutations have strong enough effect on expression. The *p*-value corresponding to Fig 7F as a function of the threshold shows gradual decrease with increasing threshold value and falls below the *p* = 0.05 level approximately at 62nd percentile, but starts to grow approximately after 80th percentile (S12 Fig). This growth may be related to either loss in statistical power with exhausting number of strong genotypes at higher threshold values, or influence of outliers in the population.

Since combinations of SNPs show a difference between the population genotypes and randomly generated genotypes and individual SNPs do not, we expect to detect consequences of this difference in the genotype scores as the number of SNPs increases. We do see that the median score for a randomly generated genotype increases as the number of SNPs accumulated in that genotype increases, while the population median stays almost unchanged, with values lying mostly below or at the 25% quartile for the simulated genotypes (Fig 7G). This illustrates that the study population ‘departs’ from the expected patterns as it adds more SNPs, starting from approximately 7 SNPs per genotype (the lowest number of SNPs for which the population median becomes smaller than a 25% quartile in the figure).

We also analyze the signed scores **Δv** for combinations of SNPs observed in the genotypes, calculating the means 〈**Δv**〉 as described above for the case of separate SNPs. This analysis reveals that the deviation of the study population from the randomly generated genotypes is associated with a specific change in the expression patterns, and not only with the general reduction of perturbation variation. In contrast to the randomly simulated genotypes, the genotypes from the study population aggregate SNPs with mainly positive means 〈**Δv**〉 (S13 Fig). Taking together the values 〈**Δv**〉 calculated for all SNPs inside each genotype, we find that the number of positive 〈**Δv**〉 is 2.8 larger than the number of negative ones for the study population, and this ratio is significantly larger than for randomly generated families (S14 Fig; *p* < 0.01 and *p* = 0.04 for the neutral and population-derived SFS, respectively). As a consequence, the distribution of the mean 〈**Δv**〉 calculated for each genotype is also shifted to positive values, as compared to a symmetrical distribution for the randomly simulated genotypes (Fig 7H). This shift becomes more pronounced for population genotypes having larger numbers of SNPs (Fig 7I, S15 Fig), since the presence of more SNPs with a positive mean 〈**Δv**〉 results in a sum with a larger mean 〈**Δv**〉 for the genotype. These results show that, on average, SNP combinations observed in genotypes from the study population mostly lead to higher expression levels than those seen in the wild type pattern (i.e., more activation than repression); however, as explained above, the magnitudes of such changes are rather low.

## Discussion

As there are large numbers of SNPs with putative regulatory effects, a fundamental challenge is to discern why some sets of sequence changes in CRMs lead to changes in gene expression, while others do not. While many CRMs are now well annotated [4–6], attempts to understand the effects of sequence variation within those CRMs are just beginning [20, 23]. A model-based understanding of how molecular pathways respond to genetic change at the level of molecular phenotypes, such as gene expression, to ultimately affect organismal phenotypes, such as propensity for disease, is a critical step in making sense of genetic variation.

As a step towards this ultimate goal, in this paper we annotated natural genomic variation in the context of a thermodynamic gene expression model and the *Drosophila* gap gene system [34]. Our goal was to determine how naturally segregating sequence variants combine in their functional consequence for gene regulation and to explore the directionality and magnitude of resulting changes.

It has been long posited that the majority of spontaneous mutations with phenotypic effects would cause only minimal perturbations, while relatively few would appear as ‘large effect.’ This notion is consistent with the inferences from QTL mapping and GWAS, but several biases make these sources of support somewhat non-robust [59]. A direct support comes from the analyses of P-element insertions. There, the distributions of effects on bristle number were seen to be symmetrical and highly leptokurtic. Indeed, a few inserts with large effects caused most of the increase in variance [60]. Here, we observe a similar ‘heavy-tailed’ distribution of mutational effects, arising naturally from our gene expression model and standing in line with some experimental studies showing that very few mutations lead to significant perturbations in expression [21, 22]. One possible interpretation is that the gap gene regulatory sequences are not ‘fine-tuned’ in this regard [23].

The thermodynamic approach to modeling transcriptional regulation of gene expression has the advantage of making it possible to predict the effects of even single-nucleotide genetic modifications on expression level. The transcriptional activation *E* in the model has statistical origins, representing a probability for activation under the thermodynamic equilibrium, and incorporates a summation over different states of the regulatory region [30, 61]. As a consequence, any change in the sequence results in the model determining a new activation level $\tilde{E}$, that must remain an equilibrium probability, but which takes into account the modified sequence (and correspondingly modified statistical weights of all other states). This means that even a local sequence perturbation such as a SNP can have a non-local response in $\tilde{E}$, because the effects of all SNPs are fit jointly, and are conditional on each other. Our calculations support the analytical prediction of non-locality of SNP influence.

It is the magnitude of the SNP effect that depends on the sequence non-locally, while the type, or sign, of this effect on the spatio-temporal expression pattern (activation, repression, or a combination of both) is determined via interplay of the polymorphic TFBS and its local vicinity. On the other hand, the chain of such local interactions, like those discussed in our paper, can be indefinitely long and involve many TFBSs, so that parameters of even distant binding sites may theoretically condition the sign of the SNP effect, thus blurring the difference between locality and non-locality. Another source of non-local conditions for the effect sign are the indirect interactions (via TFs) between TFBSs, which are not accounted for by our model and not analyzed in the paper.

Analytical derivation from the model predicts that transcriptional activation level responds to a SNP non-linearly, while numerical calculations on our population SNP data demonstrate that this non-linearity is flattened out to become an essentially additive effect for each SNP. How is it that this occurs? This question is central to quantitative genetics, as it is not generally clear how predominantly additive variation could originate from predominantly non-linear gene networks. For instance, Hill et al. [62] hypothesized that many low population frequency SNPs might contribute to phenotypic variation in an additive way (see [59] for more explanation and additional hypotheses). In our opinion, several factors participate in this transition. First, an essential part of the perturbations on the transcriptional activation level are exerted by single SNPs, with the input from other SNPs in the genotype being close to zero. Second, only a small fraction of perturbations of expression have a noticeable effect, so the regulatory score calculation on the full expression pattern is biased towards vanishing values. The complex interactions between single nucleotide mutations, predicted by the theoretical analysis and demonstrated experimentally for some enhancers [20, 23], do not show themselves by these reasons. SNPs split in ‘weak’ and ‘strong’ ones, and combination of weak and strong SNPs occurs in a way similar to the previously observed case of weak and strong enhancers [8]. In general, this illuminates how additivity might naturally arise for population variation in complex phenotypes.

The different levels of non-linearity in response to SNPs exhibited by the transcriptional activation and ultimate protein concentrations can be linked with a more general finding that gap gene expression demonstrates precision at the RNA level despite highly stochastic transcriptional activity. That finding was explained by straightforward spatio-temporal averaging, as a mechanism reducing variation in transition between the two levels [56]. In the context of SNP influence in the thermodynamic modeling approach, we may expect that similar mechanisms modulate variation of this influence observed at the transcriptional activation level and at the level of protein concentrations. In contrast, we do not see the difference between the two levels in terms of the variation of SNP influence. A possible explanation lies in the fact that the probability of transcriptional activation in the thermodynamic model is already a result of averaging across the ensemble of all configurations, and this type of averaging may be a source of noise reduction.

Our work demonstrates two types of evidence for the existence of purifying selection acting on the gap gene regulatory network. First, TFBSs of genes in the network are significantly less polymorphic than other sequence segments. A significant difference in mutation rate between TFBSs lying inside and outside the DNase I accessibility regions might in principle explain this difference [46], but our analysis controls for this. As this result can be expected according to the previous findings on conservation of TFBSs [47], it demonstrates the robustness of the procedure of TFBS selection for the model. Second, the combinations of SNPs observed in our study population genotypes result in substantially less deviation to the gene network state than SNP combinations randomly generated under the neutral site frequency spectrum. Overall, our current results complement existing examples in which a thermodynamic model was used for inter-species evolutionary simulations [39–41], as we operate on the data of natural genomic variation, which is of smaller evolutionary scale, and still see evidence for purifying selection.

Selection acts on whole organism phenotypes, but the outcome of that selection is accomplished through underlying molecular processes. Which molecular phenotypes are the primary evolutionary targets? Models of TFBS evolution have been used to infer a variety of evolutionary forces acting on the binding sites [38, 57, 63]. Our model predicts only a small correlation between the influence that SNPs exert on binding strength and on gene expression. Considering the gene network output as a more likely target for selection, it becomes unsurprising that the study population SNPs and random mutations are hardly distinguishable in terms of their properties of binding. We hypothesize that the reduced density of SNPs in TFBSs is due to stabilizing selection imposed on expression, rather than binding itself. This provides some support to previous studies hinting that there is no evidence that transcription-factor-binding strength is under strong selective constraints [64], as well as to the reported modest correlation between affinity and observed expression for mutated TFBSs [23]. We conclude that the strength of binding is under purifying selection only indirectly, through the effects it has on transcription, and the binding-to-expression map is complex due joint effects of many SNPs in many TFBSs.

It was demonstrated previously that a reliable analysis of models similar to our model should be based on the ensemble approach, involving analysis of the modeling results for many parameter sets of similar quality [65]. The results presented in this paper were obtained only for one set of parameter values. We chose these values as a representative set of the ensemble demonstrating the best visual correspondence with experimental data [34]. As a consistency test, we repeated some of the results for another set of parameter values from the ensemble. We found a similar magnitude of expression variation (S16 Fig) and confirmed the main conclusions about purifying selection in the population (S17 Fig). The *wPGP* scores of the population genotypes for the two parameter sets show a high correlation (Spearman’s *ρ* = 0.92). Therefore, our results demonstrate stability with respect to the choice of parameter values in the model.

We used the model that was fitted to the wild-type gap gene expression data, and the corresponding data approximation error determines the specificity of the model. An important question is whether this specificity is enough to study the effects of SNPs, especially taking into account that the predicted variation in expression is small for the population genotypes (Fig 2). We investigated the robustness of conclusions made in the paper by repeating the test on action of purifying selection with a set of redefined *wPGP* scores (S3 Text). These scores are similar to the score Eq (5), but quantify the perturbation in expression only for times and genes for which the model exhibits higher accuracy in a description of the wild-type expression data, i.e. exhibits a better specificity. We showed that our conclusions from the paper remain valid for all such scores (S3 Text). This analysis increases confidence in the ability of the model to study the effects of SNPs.

The general reliability of the performed analysis is based on the fact that our model has been validated in multiple ways, including tests on the expression data of known enhancers and mutant environment [34]. However, like any model, it has limitations. One limitation concerns possible incompleteness of the list of gap gene regulators considered in the model. In addition to the most important regulators that the model does account for, there are other potential candidates that are not included, such as the protein Zelda [66, 67]. This is a ubiquitous DNA-bound factor mediating chromatin accessibility and, thus, facilitating transcription-factor recruitment during the early gene expression [68]. It is possible that potential SNPs in Zelda’s binding sites may explain some of the functional variation missed by the model. Taking into account the ubiquitous nature of the factor, we may assume that the missed component of the variation should provide a homogeneous bias which would only strengthen the evolutionary insights. We generate random mutations only within TFBSs, thus excluding a possibility for a novel TFBS to appear due to such a mutation, while there are data showing importance of this phenomenon [23, 41]. Even though we believe that our pool of 889 TFBSs is representative and shows a wide range of both binding affinities and regulatory scores, we hope to consider the effect of novel sites in the future studies. Another constraint concerns the fact that the model assumes the concentrations of the TFs are constant in all genotypes. Therefore, we leave beyond the scope of this paper an important question about the impact of variation in TF input levels. The model currently does not take into account the fact that the type of influence (activation or repression) of a regulator may differ between different enhancers that control the same gene, and that enhancers may combine in relatively complex ways [7, 8]. Finally, the thermodynamic modeling approach brings constraints associated with the limiting assumptions of quasi-equilibrium, which can be relaxed, for example, by incorporating the facilitated diffusion mechanism for the formation of the molecular configurations of enhancers [69, 70]. A more thorough analysis of the data with the help of an improved model seems appealing, and we aim to elaborate our model in the future.

## Supporting information

S1 Text Analytical calculations for SNP influence in the thermodynamic model. (PDF)

S2 Text Description of SNPs underlying genotype grouping by *RMS* scores. (PDF)

S3 Text Analysis of purifying selection with a set of redefined *wPGP* scores. (PDF)

S1 Table Parameter values in the model. (PDF)

S2 Table 90 SNPs observed in the model TFBSs. The SNPs are arranged in the order of increasing genomic position. The first six columns provide the following information (from left to right): SNP index, gene, chromosome, genomic position (dm3 / BDGP5), reference and alternative nucleotides of the SNP, and SNP frequency in the study population. The next five columns present the following information about the model TFBSs containing these SNPs: TF, starting and ending positions of the binding site, reference sequence of the binding site, and its PWM-score. The table is divided into four parts, separately for each gap gene.
(PDF)

S1 Fig Variation of expression in the study population at 9 time points. The figure shows expression patterns (model solutions) at 9 time points for 213 individual genotypes and for the reference genotype, together with the observed expression patterns. The time points include the mid cleavage cycle 13 and eight time classes T1–T8 in cleavage cycle 14A, as described in Methods.
(PDF)

S2 Fig Statistical evaluation of the result from Fig 3A. (A) Results are shown for 1000 resampling experiments. For each experiment, a set of positions was randomly sampled within the model TFBSs, and each position was randomly assigned to one of the three groups of putative sign of SNP influence: 36 positions for the group of positive sign, 38 for negative sign, and 13 for alternating sign. The regulatory roles (activator or repressor) of all TFBSs containing these positions were recorded and their total number was counted for each sign group. These numbers are shown in the panel. (B)–(D) For the sampled positions from each experiment and each sign group, the ratio of the total number of activating TFBSs to the total number of repressing TFBSs was calculated. The distribution of 1000 ratio values is shown for each sign group, together with the population ratio values (red lines). The one-tailed *p*-values were estimated from these panels: *p* = 0.001 both for the positive and for the negative sign groups, and *p* = 0.45 is the minimal value of the two possible one-sided values for the group of alternating sign.
(PDF)

S3 FigDistribution of the binding affinity change caused by 90 SNPs from the study population.The probability density function is shown for $\Delta P=P_{s}-P_{s}^{\text{ref}}$, where *P*_*s*_ is the PWM score of binding site *s* containing a SNP, and $P_{s}^{\text{ref}}$ is the same score for the reference state of this site.(PDF)

S4 Fig Sign alteration of SNP influence in the study population occurs mostly due to affecting the overlap regions of multiple TFBSs (mechanism 3 from Fig 4A). (A–C) For each group of SNP influence sign (columns), the panels present the population values (red lines) for the number of SNPs appearing in the overlapping regions of multiple TFBSs as a fraction of the total number of SNPs in the group. These values are compared with the distributions of the same fraction, but for positions randomly sampled from the model TFBSs. We simulate 1000 sets of such random positions, and for each set we count the fraction of positions appearing in the overlapping region of multiple TFBSs. The total number of positions in each set equals to the number of the population SNPs from a given sign group (36, 38, and 13 positions corresponding to the groups of purely positive, purely negative, and alternating sign, respectively). (D–F) The same as in (A–C) but for the fraction of positions appearing in overlapping TFBSs of the same regulatory type (mechanisms 1 and 2 from Fig 4A). Positions associated with the sign alternating SNPs from the population appear more often in the overlap regions of multiple TFBSs than expected by chance (C; *p* < 0.001), while the frequency of their appearance in TFBSs overlapping with other TFBSs of the same type is not distinguished from randomly sampled positions (F; *p* = 0.58).
(PDF)

S5 FigDifference between genotype score and sum of SNP scores (‘deviation from additivity’) as a function of the fraction of the contribution of the strongest SNP to the variation in expression (‘strongest SNP prevalence’), for the 213 genotypes from the study population.The vertical axis shows the difference $|\Delta v_{\text{gen}}^{k}-\sum_{\text{SNP}}\Delta v_{\text{SNP}}^{k}|$, and the horizontal axis shows the ratio $(|\Delta v_{\text{SNPmax}}^{k}|-\sum_{\text{SNPi}}|\Delta v_{\text{SNPi}}^{k}|)/|\Delta v_{\text{SNPmax}}^{k}|$, where SNPmax is the strongest SNP in a given regulatory region and SNPi are all other SNPs from the same regulatory region. All designations are introduced in the part of the main text related with Fig 5.(PDF)

S6 Fig Distributions of the log-transformed absolute values of the normalized scores Δv (blue) and ΔE (red) for genotypes from the study population. The scores are normalized by the maximal values. Only non-zero scores are considered.
(PDF)

S7 Fig Effects on binding affinities from single nucleotide substitutions for the population SNPs and random mutations. (A) The distribution chart for the binding score *w*_*b*_ from Eq (9) calculated for SNPs observed in the population (red) and for all possible single mutations within the model TFBSs (yellow). The *p*-value for the comparison of the two distributions according to the bootstrap chi-square test: *p* = 0.27. (B) Comparison of the median *w*_*b*_ for the population SNPs (red) and the medians for 1000 sets randomly sampled from the all-mutations distribution (histogram); each sampled set has the same length as the data set. The one-tailed *p*-value: *p* = 0.09. (C) The same as in B, but for the means instead of medians (*p* = 0.003).
(PDF)

S8 Fig *wPGP* scores for SNPs from the study population vs. SNP binding score log-log scatter plot. (PDF)

S9 Fig SNP frequency in the study population vs. SNP *wPGP* score scatter plot and its log-log version. (PDF)

S10 Fig Distribution of the mean 〈Δv〉 for SNPs from the study population (black) and for all possible artificial SNPs in TFBSs (red). PDF stands for the probability density function. Long tails are not included in the scope of the figure.
(PDF)

S11 Fig The same as Fig 7D but with *RMS* scores used instead of *wPGP* scores. (PDF)

S12 Fig *p*-value of the result from Fig 7F as a function of the threshold separating genotypes of weak and strong effects. The threshold values were chosen as various quantiles of the *wPGP* score distribution for the population genotypes (black curve in Fig 7D).
(PDF)

S13 Fig The distributions of the mean 〈Δv〉 calculated for each SNP in a genotype, for all genotypes from the study population (black) and all genotypes from families randomly simulated under either the neutral SFS (red) or the population-derived SFS (blue). PDF stands for the probability density function. The difference between each pair of the distributions is statistically significant based on the bootstrap KS test (*p* < 0.0001).
(PDF)

S14 Fig The distribution of the ratio of the numbers of positive and negative 〈Δv〉 for SNPs from the study population (black line) and from 100 families of randomly simulated genotypes. The distributions are shown for the neutral SFS (red) and population-derived SFS (blue). The means 〈**Δv**〉 were calculated for each SNP inside each genotype in a family, and the ratio was then computed for this family. The one-tailed *p*-values: *p* < 0.01 for the neutral SFS and *p* = 0.04 for the population-derived SFS.
(PDF)

S15 Fig Analog of Fig 7I for the case of genotypes randomly mutated under the population-derived SFS. (PDF)

S16 Fig Expression patterns (model solutions) for the new set of parameter values. The spatial profiles of protein concentrations are shown at the end of cleavage cycle 14A for 213 individual genotypes and for the reference genotype, together with the observed expression patterns.
(PDF)

S17 Fig Signs of purifying selection in the analysis for the new set of parameter values. (A) The log-transformed *wPGP* score distributions for the 213 polymorphic genotypes from our study population (black), for 100 families of 213 artificial genotypes (pooled together) simulated under the neutral SFS (red), and for 100 families simulated under the population-derived SFS (blue). The dashed line marks the 2/3-quantile of the population score distribution, used for analysis in panel C. (B) The distributions of the family mean of the log-transformed *wPGP* scores for the two types of families. The black line marks the population value. *p*-values of the difference between the population and the randomly mutated genotypes: *p* = 0.01 for the neutral SFS and *p* = 0.57 for the population-derived SFS. (C) The distribution of the median *wPGP* scores calculated only for genotypes of strong effect on expression, i.e. genotypes with the score exceeding the value marked by the dashed line in panel A. The distribution corresponds to the genotypes randomly mutated under the population-derived SFS. *p*-value of the difference between the population and the randomly mutated genotypes: *p* < 0.01 for the population-derived SFS.
(PDF)
