# Supplementary material for: Translating natural genetic variation to gene expression in a computational model of the Drosophila gap gene regulatory network
Source: PLoS One. 2017 Sep 12;12(9):e0184657. doi: 10.1371/journal.pone.0184657 (PMC5595321; doi:10.1371/journal.pone.0184657)
Supplement: S1 Text — (PDF) [file pone.0184657.s001.pdf]

S1 Text: Analytical calculations for SNP influence in the thermodynamic model

1 SNPs in model equations

1.1 Promoter occupancy in thermodynamic model

Following the notation from (He et al., 2010; Kozlov et al., 2014), we write the transcriptional activation level E_i^a from the model equations (see the main text) as follows (the indices are omitted for brevity):

$$E = \frac{Z_{ON}}{Z_{ON} + Z_{OFF}} = \frac{\sum_\sigma W(\sigma)Q(\sigma)}{\sum_\sigma W(\sigma)Q(\sigma) + \sum_\sigma W(\sigma)}, \tag{1}$$

where Z_{ON} and Z_{OFF} are relative probabilities of the DNA states in which the basal transcriptional machinery is bound or not bound, respectively. These probabilities are expressed via the sum of all statistical weights $W(\sigma)$, which represent relative probabilities of all possible molecular configurations σ of the regulatory sequence. Each configuration $\sigma = \{\sigma(i)\}$ describes the set of bound and free TFBSs: $\sigma(i) = 1$ if site i is occupied and $\sigma(i) = 0$ if it's free. $Q(\sigma)$ quantifies the interactions of TFs bound in configuration σ with bound BTM. The weights W are expressed via the weights $q(S_i)$ of each binding site S_i bound in configuration σ:

$$W(\sigma) = \prod_i (\beta_i(\sigma)q(S_i))^{\sigma(i)} \prod_j \omega_{ij}^{\sigma(i)\sigma(j)}, \tag{2}$$

where ω_{ij} is a constant accounting for possible cooperative effect between the ith and jth binding sites, and β_i is a factor for repressing TFBSs implementing the short-range repression mechanism in the model: $\beta_i(\sigma) = 1$ if site i is an activating site or if it is a repressing site but is not effective in the configuration σ (the repression mechanism is not active for this site), and $\beta_i(\sigma) = \beta_i = const$ if site i is a repressing site and effective in the configuration σ. The weight q of a site $S = (S^1, \ldots, S^n)$ with nucleotides S^j depends on the concentration v of the TF and the binding affinity of the site, as follows:

$$q(S) = Kv \exp\left(P(S) - P(S_{max})\right), \quad P(S) = \sum_{j=1}^{n} P(S^j), \tag{3}$$

where K is the affinity constant for the strongest (consensus) site S_{max} of the TF, and P is the PWM-score of a site, which is calculated from the independent contributions from all constituent nucleotides. Apart from the modifications described in our previous work (Kozlov et al., 2014), formulas (1)–(3) are identical to those proposed in (He et al., 2010).

The PWM-score of a sequence segment is calculated from the positional weight matrix that defines the affinity of each position in the segment to the TF relative to some background value. We choose the following form for this matrix (Lifanov et al., 2003):

$$P_{\alpha,j} = \log \frac{x_{\alpha,j} + ab_\alpha}{(N + a)b_\alpha}, \quad a = \log N, \tag{4}$$

where $P_{\alpha,j}$ is a matrix element with α and j indexing the four nucleotide letters (rows) and the position in the binding site (columns), respectively; $x_{\alpha,j}$ is the marginal frequency of a nucleotide α at a position j (an element of the position count matrix, or PCM), resulting from alignment of a given motif collection; N is the total weight of the alignment, equal to the sum of any column in the PCM; a is the pseudocount; and b_α is the background probability of nucleotide α. Therefore, the score $P(S)$ is calculated via eq. (3) where $P(S^j) = P_{S^j,j}$ from eq. (4).

1.2 Single SNP in regulatory sequence

Consider a SNP in jth position of TFBS S, caused by a substitution $S^j \to \tilde{S}^j$, leading to the site S being substituted by its mutant version $\tilde{S}$. This one nucleotide substitution leads to the replacement $x_{S^j,j} \to x_{\tilde{S}^j,j}$ in the corresponding term $P(S^j)$ and, consequently, to the change of the PWM-score $P(S)$ and the statistical weight $q(S)$ of the site, according to eqs. (3) and (4). We account for the difference between the frequencies $x_{S^j,j}$ and $x_{\tilde{S}^j,j}$ by means of the quantity δ defined as follows:

$$\delta = \gamma \left(\frac{x_{\tilde{S}^j,j}}{b_{\tilde{S}^j}} - \frac{x_{S^j,j}}{b_{S^j}} \right), \quad \gamma = (N+a)^{-1}. \tag{5}$$

Therefore, δ can be viewed as the first manifestation of the SNP in the model equations. The statistical weight of the mutated TFBS can be expressed via the weight of the original TFBS in the following simple form:

$$q(\tilde{S}) = q(S) + q(\bar{S})\delta, \quad \bar{S} = (S^1, \ldots, S^{j-1}, S^{j+1}, \ldots, S^n), \tag{6}$$

where $\bar{S}$ is the reduced binding site, obtained by deleting jth position in site S. The statistical weight $q(\bar{S})$ is calculated via eq. (3)–(4) in which the jth column of the PWM (corresponding to the jth position in the motif) should be removed.

To see how δ is involved in the promoter occupancy E, we first note that the relative probabilities Z_{OFF} and Z_{ON} linearly depend on the statistical weight q of any chosen TFBS, so we can write:

$$Z_{OFF} = Z^0_{OFF} + q(S)Z^1_{OFF}, \tag{7}$$

and similarly for Z_{ON}. The superscripts '0' and '1' of the terms in this and further similar q expansions are chosen to indicate the power of q related with these terms. Here, neither Z^0_{OFF} nor Z^1_{OFF} contain $q(S)$. Z^0_{OFF} represents the total statistical weight of all states where site S is free, and qZ^1_{OFF}, where it is bound. Substituting (7) for the relative probabilities $\tilde{Z}_{OFF}$ and $\tilde{Z}_{ON}$ describing the regulatory sequence with the SNP in site S, and taking into account the connection between the statistical weights $q(S)$ and $q(\tilde{S})$, we have:

$$\tilde{Z}_{OFF} = \tilde{Z}^0_{OFF} + \tilde{q}\tilde{Z}^1_{OFF} = Z^0_{OFF} + (q + \bar{q}\delta)Z^1_{OFF} = Z_{OFF} + \bar{q}Z^1_{OFF}\delta, \tag{8}$$

and similarly for $\tilde{Z}_{ON}$. Here, we note that $\tilde{Z}^0_{OFF} = Z^0_{OFF}$, and similarly for $\tilde{Z}^1_{OFF}$, because these sums do not depend on site $\tilde{S}$ and, hence, are not changed by the SNP. For simplicity, we also write $\tilde{q} = q(\tilde{S})$ and $\bar{q} = q(\bar{S})$. The term $\bar{q}Z^1_{OFF}$ in (8) has an obvious interpretation: If we replaced site S in the regulatory sequence by its reduced version $\bar{S}$ (with the polymorphic position removed) and computed its statistical weight as described above, this term would represent the relative probability of all states in which this reduced site is bound. We note that the only information about the SNP in eq. (8) is in δ, which can be interpreted as the strength of the perturbation exerted by the SNP.

Finally, for the promoter occupancy of the mutated system, we have:

$$\tilde{E} = \frac{\tilde{Z}_{ON}}{\tilde{Z}_{ON} + \tilde{Z}_{OFF}} = \frac{Z_{ON} + \bar{q}Z^1_{ON}\delta}{Z_{ON} + Z_{OFF} + \bar{q}(Z^1_{ON} + Z^1_{OFF})\delta}. \tag{9}$$

There are two conclusions we can draw from this expression. First, the terms in the numerator and denominator incorporate the relative probabilities for a system in which site S can have three possible states: free, bound in its original form (without the SNP, with statistical weight q), and bound in its reduced form with statistical weight $\delta\bar{q}$. Second, the ultimate effect that the SNP has on promoter occupancy is caused not only by the TFBSs that physically interact with the mutated site (for example, via the cooperative effect), but by all binding sites in the regulatory sequence. This happens because all sites participate in determining the corresponding relative probabilities for site S being free or bound, and, as a consequence, changes to these probabilities due to the modification of the statistical weight of this site is modulated by the weights of other binding sites. Therefore, SNPs have non-local effects in the thermodynamic model of gene expression.

1.3 Multiple SNPs in regulatory sequence

Formula (9) can be extended to the case of multiple SNPs in the regulatory region. We suppose that each of I binding sites S_1, S_2, ..., S_I in this region contains one SNP, and these SNPs correspond to the perturbations $\delta(1)$, $\delta(2)$, ..., $\delta(I)$ as defined by eq. (5). The promoter occupancy in this case has the form:

$$\tilde{E} = \frac{Z_{ON} + \sum_{k=1}^{I} \sum_{i_1 \cdots i_k} G(i_1, \ldots, i_k)\delta(i_1)\cdots\delta(i_k)}{Z_{ON} + Z_{OFF} + \sum_{k=1}^{I} \sum_{i_1 \cdots i_k} F(i_1, \ldots, i_k)\delta(i_1)\cdots\delta(i_k)}, \tag{10}$$

where indices i_1, ..., i_k run over all values fulfilling $1 \leq i_1 \leq i_2 \leq \cdots \leq i_k \leq I$, and

$$\begin{aligned} G(i_1, \ldots, i_k) &= \bar{q}(i_1)\cdots\bar{q}(i_k) Z_{ON}^{i_1 \cdots i_k}, \\ F(i_1, \ldots, i_k) &= \bar{q}(i_1)\cdots\bar{q}(i_k) \left(Z_{OFF}^{i_1 \cdots i_k} + Z_{ON}^{i_1 \cdots i_k} \right), \end{aligned} \tag{11}$$

where $\bar{q}(i)$ denotes the statistical weight of the reduced version of site i, $\bar{q}(i_1)\cdots\bar{q}(i_k) Z_{OFF}^{i_1 \cdots i_k}$ is the total statistical weight of all states in which the sites i_1, ..., i_k are in their reduced forms and bound. The 'ON' term in (11) reads similarly.

Formula (10) corresponds to the case in which sites S_i do not mutually overlap. The overlap effectively reduces the nonlinearity in this formula; namely, if sites S_i and S_j overlap, there are no terms containing the product $\delta(i)\delta(j)$.

2 Sign of SNP influence

In this section, we analyze the sign of the influence that a SNP exerts on the transcriptional activation level E. This sign can be positive if E increases in SNP presence (activating action), negative if E decreases (repressive action), and it is zero if the activation state of the target gene does not change in SNP presence. This sign can be constant for a given SNP, or it may change depending on the state of other TFBSs. We study situations when this change is theoretically possible. The analysis presented here assumes that the indirect interactions between TFBSs (via their influence on TFs) are small enough to be neglected.

The sign of SNP influence can be deduced as follows. Let us suppose that a SNP falls into a TFBS with the statistical weight q. The SNP can either increase or decrease the binding affinity (and, thus, the weight q) of this site, and the sign of the change of q can never vary (assuming no indirect interactions between TFBSs). Given the sign of SNP influence on q, the sign of the influence on E is then determined by calculating the derivative $\partial E/\partial q$. Taking E from Eq. (1) and writing Z_{ON} and Z_{OFF} as in Eq. (7), we get the following expression for the

derivative:

$$\frac{\partial E}{\partial q} = \frac{Z^0_{OFF}Z^1_{ON} - Z^1_{OFF}Z^0_{ON}}{\left(Z_{OFF} + Z_{ON}\right)^2}. \tag{12}$$

As the denominator of this expression is always positive, the sign of $\partial E/\partial q$ coincides with the sign of the term:

$$\xi = Z^0_{OFF}Z^1_{ON} - Z^1_{OFF}Z^0_{ON}. \tag{13}$$

Note that ξ does not contain q but includes statistical weights of all other TFBSs and other regulatory parameters, so the specific balance between these weights and parameters determine the sign of ξ.

Denoting via $\Delta q = \tilde{q} - q$ the change in the weight of the site due to SNP presence, we get the positive sign of SNP influence (activation) if the signs of Δq and $\partial E/\partial q$ coincide, i.e. either $\Delta q > 0$ and $\partial E/\partial q > 0$ or $\Delta q < 0$ and $\partial E/\partial q < 0$. The SNP influence has the negative sign (repression) if Δq and $\partial E/\partial q$ differ in sign. Finally, the influence is absent if either Δq or $\partial E/\partial q$ is zero. In what follows, we study conditions leading to alternating sign of SNP influence. We start with general formulas and then consider specific examples.

2.1 Local context determines the sign of SNP influence

First, we show that any part of the regulatory sequence which does not interact (in a specific way) with the polymorphic TFBS cannot change the sign of the SNP influence. A TFBS can 'interact' with another TFBSs via several mechanisms. They include overlapping of the sites, the cooperative binding to these sites, or the participation of one site in the short-range repression of the other one (a detailed description of all possible mechanisms can be found in (He et al., 2010)).

Let us consider a regulatory region R consisting of two subregions R_1 and R_2: $R = R_1 \cup R_2$. If none of the TFBSs from the subregion R_1 interacts (in the above mentioned sense) with none of the TFBSs from the subregion R_2, the statistical weight $Z_{OFF}(R)$ for this regulatory region can be decomposed too: $Z_{OFF}(R) = Z_{OFF}(R_1)Z_{OFF}(R_2)$, where $Z_{OFF}(R_i)$ is the sum of the statistical weights of all possible configurations of TFBSs from R_i. In other words, R_1 and R_2 are independent subregions, so that nothing happening with the TFBSs in R_1 impacts the probability of any configuration of R_2, and vice versa. This decomposition of the statistical weight Z_{OFF} can be viewed as a mathematical definition of the independence of two (or more) subregions of the regulatory region in terms of TF binding in these regions. Similarly, we can define the independence of two subregions in terms of their influence on transcription activation: $Z_{ON}(R) = Z_{ON}(R_1)Z_{ON}(R_2)$. This type of independence is determined by an activation mechanism implemented in the model and, in the general case, not the same as the independence in terms of binding.

Consider now the set R_D including a binding site S with weight q and all TFBSs interacting (in terms of binding) with this site, and let R_N be the set of all TFBSs independent (in terms of binding) of R_D. We can write for the full statistical sum:

$$Z_{OFF} = Z_{OFF}(R_N)Z_{OFF}(R_D) = Z_{OFF}(R_N)\left(Z^0_{OFF}(R_D) + qZ^1_{OFF}(R_D)\right). \tag{14}$$

This formula is the analog of Eq. (7) where we take into account that the statistical sum for the region R_N, independent of the 'local' vicinity R_D of the site S, is a common factor in Z^0_{OFF} and Z^1_{OFF}. The dependency of the site S on its local vicinity R_D means that $Z^0_{OFF}(R_D) \neq Z^1_{OFF}(R_D)$.

If the site S contains a SNP, its ξ from Eq. (13) takes the form:

$$\xi = Z_{OFF}(R_N)\left(Z^0_{OFF}(R_D)Z^1_{ON} - Z^1_{OFF}(R_D)Z^0_{ON}\right). \tag{15}$$

Since $Z_{OFF}(R_N) > 0$, it is clear that the TFBSs from R_N do not affect the sign of the influence of the SNP from the site S (via the Z_{OFF} terms; some of those sites may affect the sign via the Z_{ON} terms in case they are dependent on the local vicinity of the site S in terms of the influence on transcription activation). Extracting similarly the subregion independent of the local vicinity of S in terms of the influence on transcription activation and adding the corresponding common factor to the expression (15), we can find the actual set of the TFBSs forming the local vicinity of the site S that determine the sign of the SNP influence.

2.2 Example of an isolated polymorphic TFBS-activator

As an extreme example, let us consider the case when the polymorphic site S binds a TF-activator and is completely independent of all other TFBSs, so that the vicinity R_D contains only this site. In this case, $Z^0_{OFF} = Z^1_{OFF}$, and $Z_{OFF} = (1+q)Z^0_{OFF}$. The direct calculations lead to the following formula for Z^1_{ON}, under the assumption about additivity of the activating actions from multiple bound TFs-activators implemented in the model (Kozlov et al., 2014):

$$Z^1_{ON} = Z^0_{ON} + \alpha Z^0_{OFF}, \tag{16}$$

where α ($\alpha > 1$) is the strength of the activating action from the bound S. Given these formulas, we have $\xi = (Z^0_{OFF})^2\alpha > 0$. Therefore, in agreement with the general formulas above, the absence of any 'dependent' TFBSs leads to the definite sign of the derivative $\partial E/\partial q$, and this sign is positive for the SNP in the binding site for a TF-activator. This means that, if the SNP increases (decreases) the binding affinity of the site, it provides the purely activating (repressing) action on gene expression.

In what follows, we will provide expressions for ξ in cases when the alternating sign (i.e., $\xi > 0$ under some conditions, and $\xi < 0$ under others) is possible. These cases are illustrated in the figure below, which is a copy of Fig. 2C from the main text. For notational convenience, we will assume for all configurations shown in the figure (except for the case 2, see below) that there are no other TFBSs in the regulatory region except the depicted ones. As explained above, this assumption can be weakened without reducing generality if we suggest that all other sites which are not depicted are independent (both in terms of binding and in terms of action on transcriptional activation) of the depicted sites, i.e. each depicted configuration represents an isolated part of the regulatory region.

2.3 Overlap of TFBSs of the same type (cases 1 and 2)

The case 1 from the figure corresponds to the polymorphic site-activator S (with the weight q and the activation strength $\alpha > 1$) overlapping with another site-activator (with the weight q_1 and the activation strength $\alpha_1 > 1$). We have:

$$Z^0_{OFF} = 1 + q_1, \quad Z^1_{OFF} = 1, \tag{17}$$

$$Z^0_{ON} = 1 + \alpha_1 q_1, \quad Z^1_{ON} = \alpha, \tag{18}$$

$$\xi = \alpha - 1 + q_1(\alpha - \alpha_1). \tag{19}$$

It follows from these expressions that $\partial E/\partial q$ is positive if

$$\alpha > \frac{1 + \alpha_1 q_1}{1 + q_1} \tag{20}$$

and negative otherwise. In particular, the derivative is always positive when $\alpha \geq \alpha_1$, i.e. if the polymorphic site is a stronger activator than the site-activator it overlaps with, in which case

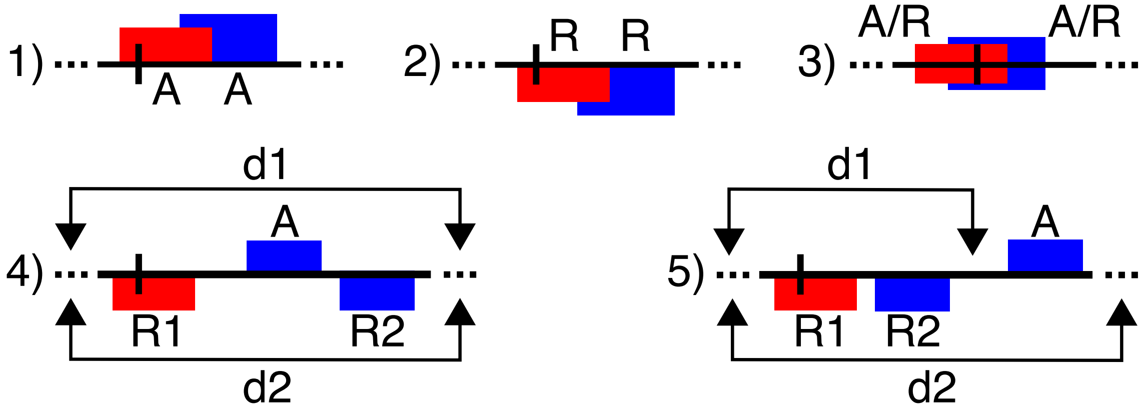

Figure: Local interactions of a polymorphic TFBS (red box) with other TFBSs (blue boxes) leading to alternating sign of SNP influence on expression. The horizontal lines represent segments of the regulatory region. Activating (repressing) TFBSs are shown above (below) these lines and labeled with 'A' ('R'). The SNP position is marked via a short vertical line. In 1) and 2), the polymorphic site overlaps with another site of the same type. In 3), the SNP appears in the overlap region of two TFBSs, so both sites are polymorphic in this picture. They are shown at the center of the DNA line to express that both sites can be either activating or repressing (in any combination) in this situation. In 4) and 5), $d1$ and $d2$ indicate the range of repression from sites R1 and R2, respectively. The activating site is repressed by both repressors in 4) and only by R2 in 5).

the state of the polymorphic site is more important in the competition with the overlapping site. Note that the relation (20) depends on the statistical weight q_1, so varying its value may lead to alternative sign of the SNP influence. This weight depends on the TF concentration, which changes in time and space, and, hence, this change is potential for the sign of the SNP influence to vary in time and space as well.

Consider now the case 2 from the figure, where the polymorphic site-repressor S (with the weight q and the repression strength $\beta > 0$) overlaps with another site-activator (with the weight q_1 and the repression strength $\beta_1 > 0$). The repression in the model is represented as a short-range action (Kozlov et al., 2014; He et al., 2010). A TF-repressor may bind to its site in two forms, either an effective or ineffective. The repression takes place if the effective binding occurs: the bound TF makes a vicinity of the binding site inaccessible for all TFs except other repressor molecules, and those repressor molecules are allowed to bind also only in the effective form. The repression parameters β and β_1 participate in the model equations as the weights in the statistical weights (see Eq. (2)) for those configurations in which the TF-repressors are bound to their sites in the effective form.

As the short-range repression does not influence E directly, but only through forbidding activators to bind, the derivative $\partial E/\partial q$ is always zero for the site-repressor S if there are no sites-activators placed in the repression range of S. Therefore, in addition to the depicted two sites-repressors in the case 2 in the figure, we assume that their repression range includes a site-activator with the weight q_2 and the activation strength $\alpha_2 > 1$. Then, we have:

$$Z^0_{OFF} = 1 + q_1(1+\beta_1) + q_2 + q_1 q_2, \quad Z^1_{OFF} = 1 + \beta + q_2, \tag{21}$$

$$Z^0_{ON} = 1 + q_1(1+\beta_1) + \alpha_2(q_2 + q_1 q_2), \quad Z^1_{ON} = 1 + \beta + \alpha_2 q_2, \tag{22}$$

$$\xi = q_2(\alpha_2 - 1)(\beta_1 q_1 - \beta(1+q_1)). \tag{23}$$

It follows from these expressions that $\partial E/\partial q$ is positive if

$$\beta < \frac{\beta_1 q_1}{1+q_1} \tag{24}$$

and negative otherwise.

2.4 SNP in two TFBSs (case 3)

When a SNP appears in the overlap region of two TFBSs (case 3 in the figure), it affects affinities of both sites. Therefore, we should analyze the second derivative $\partial^2 E/(\partial q_1 \partial q_2)$ with respect to the weights q_1 and q_2 of the polymorphic sites. As for the the first derivative, the denominator of $\partial^2 E/(\partial q_1 \partial q_2)$ is always positive, so it can be omitted for the analysis of the sign. We denote the numerator also by ξ. Direct calculations reveal that ξ has alternating sign regardless of whether the overlapping sites are of the same or different types.

If both TFBSs are activators with the activation parameters α_1 and α_2, we have:

$$Z_{OFF} = 1 + q_1 + q_2, \quad Z_{ON} = 1 + \alpha_1 q_1 + \alpha_2 q_2, \tag{25}$$

$$\xi = 2 + q_1(1+\alpha_1)(\alpha_1 - \alpha_2) - 2\alpha_1\alpha_2 - q_2(1+\alpha_2)(\alpha_1 - \alpha_2). \tag{26}$$

It is evident that ξ has alternating sign depending on the parameters. However, when $\alpha_1 = \alpha_2$, ξ is always positive, taking into account that $\alpha_1 > 1$ and $\alpha_2 > 1$.

If the site with q_1 is an activator with the activation parameter α and the site with q_2 is a repressor with the repression parameter β, we have:

$$Z_{OFF} = 1 + q_1 + q_2(1+\beta), \quad Z_{ON} = 1 + \alpha q_1 + q_2(1+\beta), \tag{27}$$

$$\xi = (\alpha - 1)(1+\beta)\left(q_1(1+\alpha) - 2(1 + (1+\beta)q_2)\right). \tag{28}$$

Similarly, it can be shown that the second derivative $\partial^2 E/(\partial q_1 \partial q_2)$ has alternating sign in the case when the two overlapping sites are repressors and they repress a third site-activator (the expressions are more complicated in this case).

2.5 SNP in a site-repressor competing with another site-repressor (cases 4 and 5)

In the case 4 from the figure, we assume that the polymorphic site-repressor (R1) has the statistical weight q and the repression strength β, and these quantities are q_1 and β_1 for the second site-repressor (R2). The site-activator (A), repressed by both site-repressors, has the weight q_2 and the activation strength α. This leads to the following ξ from Eq. (13):

$$\xi = q_2(\alpha - 1)(1+q_1)\left(\beta_1 q_1 - \beta(1+\beta_1 q_1)\right), \tag{29}$$

which means that ξ is positive if

$$\beta < \frac{\beta_1 q_1}{1+\beta_1 q_1}, \tag{30}$$

and negative otherwise.

We leave the same designations for the case 5 in the figure. Now the polymorphic site-repressor does not repress the site-activator, but its repression range d_1 contains the second site-repressor. In this case, we have:

$$\xi = -q_1 q_2(\alpha - 1)\beta_1\left(\beta q_1 - q_1 - 1\right), \tag{31}$$

which means that ξ is positive if

$$\beta < \frac{1+q_1}{q_1}, \tag{32}$$

and negative otherwise.

The alternating sign in the case 5 can be explained as follows. In the model, if a binding site for a repressor is bound effectively, the short-range repression starts to act in the repression range of this site. If there are other sites for repressors appear in this range (as in the cases 4 and 5 from the figure), they can be bound only in the effective form. For example, if site R1 in the case 5 is bound in the effective form (with the term βq in the statistical weight of the corresponding configuration), the site R2 also can be bound only in the effective form in this configuration (with the term $\beta_1 q_1$, and not with the term q_1 for the ineffective binding). In other words, the effective form of bound R1 does not prevent R2 from its repressive action. Now suppose that R1 is bound ineffectively (with the term q in the statistical weight of the corresponding configuration). The presence of R1 in the repression range of R2 means that such configuration does not allow binding to R2 in the effective form (with the term $\beta_1 q_1$), and this means that R2 does not act as a repressor and the site-activator can be bound. Therefore, there are two opposite ways of influence from the polymorphic site: the state βq promotes repressive action, and the state q implicitly promotes activation. The conflict between these types of influence yields the alternating sign of the derivative $\partial E/\partial q$.

We note that all cases considered above remain sign-alternating when cooperative binding is added, but the cooperative binding alone is not able to change the sign of SNP influence in these cases.

References

Xin He, Md. Abul Hassan Samee, Charles Blatti, and Saurabh Sinha. Thermodynamics-based models of transcriptional regulation by enhancers: The roles of synergistic activation, cooperative binding and short-range repression. *PLoS Computational Biology*, 6:e1000935, 2010.

Konstantin Kozlov, Vitaly Gursky, Ivan Kulakovskiy, and Maria Samsonova. Sequence-based model of gap gene regulatory network. *BMC Genomics*, 15 Suppl 12:S6, December 2014. doi: 10.1186/1471-2164-15-S12-S6.

Alexander P. Lifanov, Vsevolod J. Makeev, Anna G. Nazina, and Dmitri A. Papatsenko. Homotypic regulatory clusters in drosophila. *Genome Research*, 13(4):579–588, 2003. doi: 10.1101/gr.668403.
