# Supplementary material for: Translating natural genetic variation to gene expression in a computational model of the Drosophila gap gene regulatory network
Source: PLoS One. 2017 Sep 12;12(9):e0184657. doi: 10.1371/journal.pone.0184657 (PMC5595321; doi:10.1371/journal.pone.0184657)
Supplement: S2 Text — (PDF) [file pone.0184657.s002.pdf]

S2 Text: Description of SNPs underlying genotype grouping by RSS scores

The RMS scores for genotypes from the study population group into two visually distinct clusters of values (Figure 1) and one outlier (RMS score = 10.7).

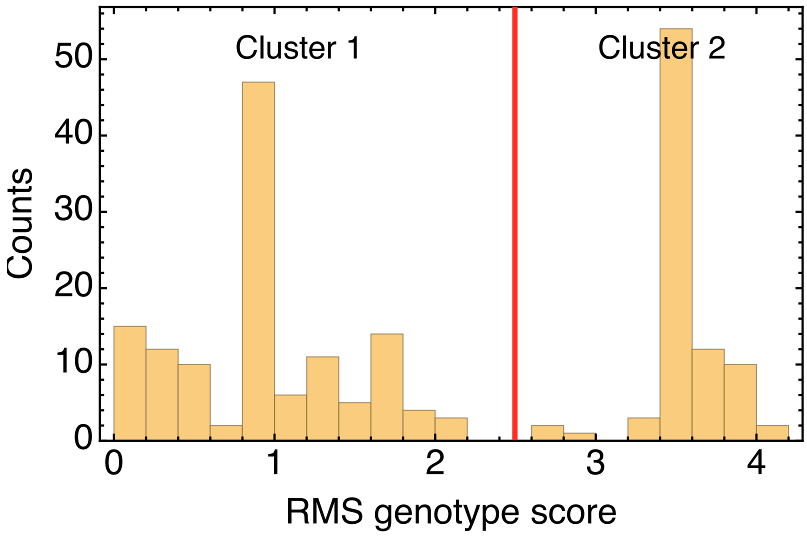

Figure 1. Genotype scores based on the RMS measure. The red line separates two clusters of the score values; the outlier (score = 10.7) is not shown.

The outlying genotype contains 11 SNPs in the regulatory regions, but only one of these SNPs contributes in a substantive way to the genotype score. This SNP is the one that has strongest effect in the study population and has the following parameters:

Global coordinate	TFBS	Target gene	CRM
2 329 021	2 sites for Giant	*giant*	*gt_−6*

The SNP appears in the *gt_−6* module of the *giant* regulatory region, which controls the anterior expression of the gene [1]. The model solution for the genotype with this SNP exhibits a significant decrease in the anterior expression domain for *giant* (see Figure 1 from the main text).

Clusters 1 and 2 contain 129 and 84 genotypes, respectively. Some SNPs are shared between genotypes from the two clusters, while others are specific to a given cluster. To understand which SNPs determine the clustering, we classified all SNPs from all genotypes into three sets: a SNP is included in set 1 if it appears in one or several genotypes from cluster 1 and does not appear in any genotype from cluster 2; a SNP is included in set 2 if it appears in one or several genotypes from cluster 2 and does not appear in any genotype from cluster 1; and a SNP is included in set 3 if it is included in at

least one genotype from each cluster. In other words, sets 1 and 2 contain SNPs unique for genotypes from cluster 1 and 2, respectively. Figure 2 shows how the regulatory scores of SNPs are distributed over these three sets.

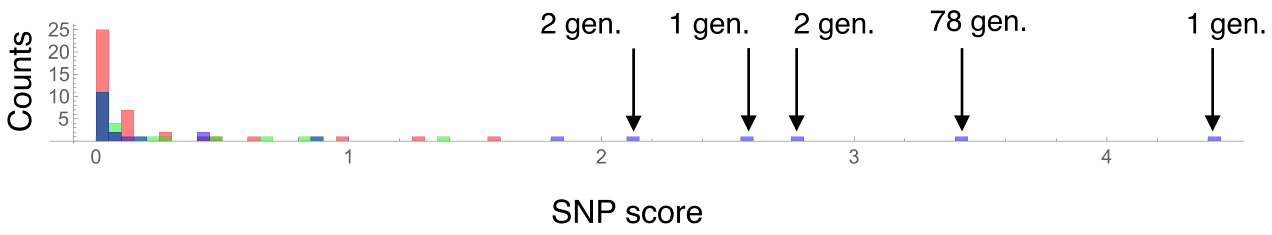

Figure 2. Distribution of RMS scores for SNPs in the three sets of SNPs: set 1 (green), set 2 (blue), and set 3 (red). Five strongest SNPs are marked by arrows with indication of how many genotypes contain each of these SNPs. The outlier is excluded from the analysis.

The analysis shows that the five strongest SNPs (shown in Figure 2) are enough to determine the clustering of genotypes. Namely, each genotype from cluster 2 contains at least one of these five SNPs, and no genotypes from cluster 1 contain any of these SNPs. This fact is not true if we consider any subset of these five SNPs; therefore, given the additivity of SNP effects, these SNPs form the minimal set of SNPs characteristic for the clusterization. The table below presents parameters of these five SNPs (the SNPs in the table are ordered by decreasing regulatory score, with the highest score on the top):

Global coordinate	TFBSs	Target gene	CRM
21 113 582	2 sites for Giant,<br>1 site for Hunchback	*Kruppel*	*Kr_SN1.7KrZ*
2 325 239	1 site for Knirps,<br>1 site for Hunchback	*giant*	*gt_3, gt_CE8001*
4 526 964	2 sites for Hunchback,<br>1 site for Caudal	*hunchback*	*hb_HZ1.4, hb_HZ526,<br>hb_upstream_enhancer*
20 693 004	1 site for Hunchback	*knirps*	*kni_-5, kni_anterioventral*
21 113 423	1 site for Knirps	*Kruppel*	*Kr_SN1.7KrZ*

All annotated CRMs are according to the REDfly database [2].

References

[1] Schroeder, M. et al. (2004) Transcriptional Control in the Segmentation Gene Network of Drosophila. PLoS Biology 2, e271.

[2] REDfly database (Release 5; http://redfly.ccr.buffalo.edu). Gallo, S.M., Gerrard, D.T., Miner, D., Simich, M., Des Soye, B., Bergman, C.M. and Halfon, M.S. (2010). REDfly v3.0: Toward a comprehensive database of transcriptional regulatory elements in Drosophila. *Nucleic Acids Res.*; doi: 10.1093/nar/gkq999.
