# Supplementary material for: Translating natural genetic variation to gene expression in a computational model of the Drosophila gap gene regulatory network
Source: PLoS One. 2017 Sep 12;12(9):e0184657. doi: 10.1371/journal.pone.0184657 (PMC5595321; doi:10.1371/journal.pone.0184657)
Supplement: S3 Text — (PDF) [file pone.0184657.s003.pdf]

S3 Text: Analysis of purifying selection with a set of redefined $wPGP$ scores

Main idea

The hybrid model of expression described in the main text was fitted to the wild-type gap gene expression data and validated as described by Kozlov et al. (2014). The wild-type solution of the model equations approximates the wild-type data with some error $\epsilon(x, t, a)$, which in some spatiotemporal domains (for some x and t) and for some genes a exceeds the expression variation predicted by the model for the population genotypes (see, for example, the data and the model solutions in Figure 2 of the main text). Therefore, a question arises whether the model has the power to study the effects of SNPs.

As a possible way to tackle this problem, we suggest the following analysis which should demonstrate the robustness of the conclusions made in the main text with respect to variation of the approximation error shown by the model. This analysis is based on a redefined $wPGP$ score, which is a measure of the perturbation in the modelled expression exerted by genetic variation and, thus, a measure underlying fitness of an individual genotype in the population. The $wPGP$ score used in the main text sums up the perturbations of the spatial profiles of expression for all times t and gap genes a, even though different (t, a) are related with different approximation errors ϵ. The main idea of the suggested analysis is to arrange these (t, a)-dependent perturbations according to decreasing values of the error ϵ and define a new $wPGP$ score by subtraction of those (t, a)-dependent perturbations which are associated with the largest error ϵ. Therefore, the new score will be the measure of perturbation taking into account only those (t, a) pairs which correspond to a small error ϵ. If we were able to reproduce the results from the main text for this new score, this would demonstrate the robustness of the conclusions in regard to the variable approximation error and, thus, increase the general reliability of these conclusions. We do this for the analysis of purifying selection in more details in what follows.

Redefined $wPGP$ scores

The $wPGP$ score w is defined by eqs. (5)–(6) of the main text:

$$w = \sum_{t,a} f^a(t), \quad f^a = 0.5 - 0.5 * (\text{reward} - \text{penalty}), \tag{1}$$

where, for given gene a and time t, the 'reward' and 'penalty' terms are calculated from the spatial expression pattern as follows:

$$\text{reward} = \frac{\sum_i V_i^a \min(V_i^a, v_i^a)}{\sum_i (V_i^a)^2}, \quad \text{penalty} = \frac{\sum_i \max(0, v_i^a - V_i^a)(V_{\max}^a - V_i^a)}{\sum_i (V_{\max}^a - V_i^a)^2}, \tag{2}$$

where $v_i^a = v_i^a(t)$ is the protein concentration in the model solution for the mutated genotype (for nucleus i, gene a, and time t), V_i^a is the same for the reference genotype (the wild-type solution), and $V_{\max}^a = V_{\max}^a(t)$ is the maximum value of the spatial expression pattern V_i^a for a given time. For the reference genotype, i.e. when there are no mutations, we have $w = 0$, and $w > 0$ when mutations are introduced in the sequence.

$f^a(t)$ represent the (t, a)-dependent scores of the perturbed spatial expression profiles $v_i^a(t)$. We can map these scores to the values of the approximation error ϵ introduced above. This error quantifies the difference between the gap gene expression patterns in the model for the reference genome and the wild-type expression data via the *wPGP* measure as follows:

$$\epsilon = \sum_{t,a} \epsilon^a(t), \tag{3}$$

where $\epsilon^a(t) = f^a(t)$ from eqs. (1)–(2) in which v_i^a is replaced with the model solution for the reference genome and V_i^a is replaced with the wild-type expression data. The values of $\epsilon^a(t)$ are shown in Fig. 1 together with the wild-type expression patterns. We wish to sort these patterns according to the data approximation quality of the model. These patterns are indexed by the pairs (t, a), so we arrange these pairs in the order of increasing values of error $\epsilon^a(t)$; for this purpose, we introduce the index $1 \leq j \leq 36$ and the dependences $a = a(j)$, $t = t(j)$, so that

$$\epsilon^{a(j)}\left(t(j)\right) \leq \epsilon^{a(j+1)}\left(t(j+1)\right), \quad j = 1, \ldots, 35. \tag{4}$$

The arranged errors $\epsilon^{a(j)}\left(t(j)\right)$ are shown in Fig. 2. Therefore, we classified the pairs (t, a) according to the data approximation quality of the model.

We wish to use this classification in the score w from (1). We define new scores $w^{\text{new}}(k)$ by summing not all $f^a(t)$, but only those which correspond to pairs (t, a) with smaller approximation error $\epsilon^a(t)$, starting with the smallest error and adding more and more terms as k increases:

$$w^{\text{new}}(k) = \sum_{j=1}^{k} f^{a(j)}\left(t(j)\right), \quad k = 1, \ldots, 36. \tag{5}$$

The meaning of the new scores can be explained as follows. Given a mutated genotype, the score $w^{\text{new}}(k)$ quantifies the resulted total perturbation in the model solution for this genotype accounted only for those times t and genes a which correspond to k smallest errors $\epsilon^a(t)$. In other words, when we use the scores $w^{\text{new}}(k)$ with small values of index k, we concentrate on selected spatiotemporal states of the gene network which are described by the model with high accuracy, in contrast to the full state that may be approximated by the model with large errors. However, the cost of this refinement is the loss in statistical power of any analysis with the new scores and small values of k due to less information about the network states encoded in such scores.

As we aim at describing a state of the network consisting of four genes, we further take only those values of k for which at least one f^a for each of the four genes $(a = 1, \ldots, 4)$ is present in the sum in eq. (5). The calculations show that this condition holds for $k \geq 13$, so we consider in total 24 scores $w^{\text{new}}(k)$, $k = 13, \ldots, 36$. By construction, $w^{\text{new}}(36) = w$, which is the full score used in the main text. In the next section, we repeat one of the analysis from the main text using the new scores.

We should note that we characterise the data approximation error of the model by breaking the 'full gene network state' into the spatial expression profiles (like in Fig. 1), indexed by the pairs (t, a). As the approximation error may also vary within the spatial domain, i.e. for any fixed (t, a), it would also be informative to break the spatial domain into parts related with different values of the approximation error. However, it is not feasible for the *wPGP* measure, since the essence of this measure is in its ability to more properly assess the spatial

features of expression (Kazemian et al., 2010; He et al., 2012; Samee and Sinha, 2013). It would be appropriate for the RMS score, but, as discussed in the cited papers, this score is less accurate than $wPGP$ and, as we showed in the main text, it even leads to different predictions. Therefore, we do not consider this score.

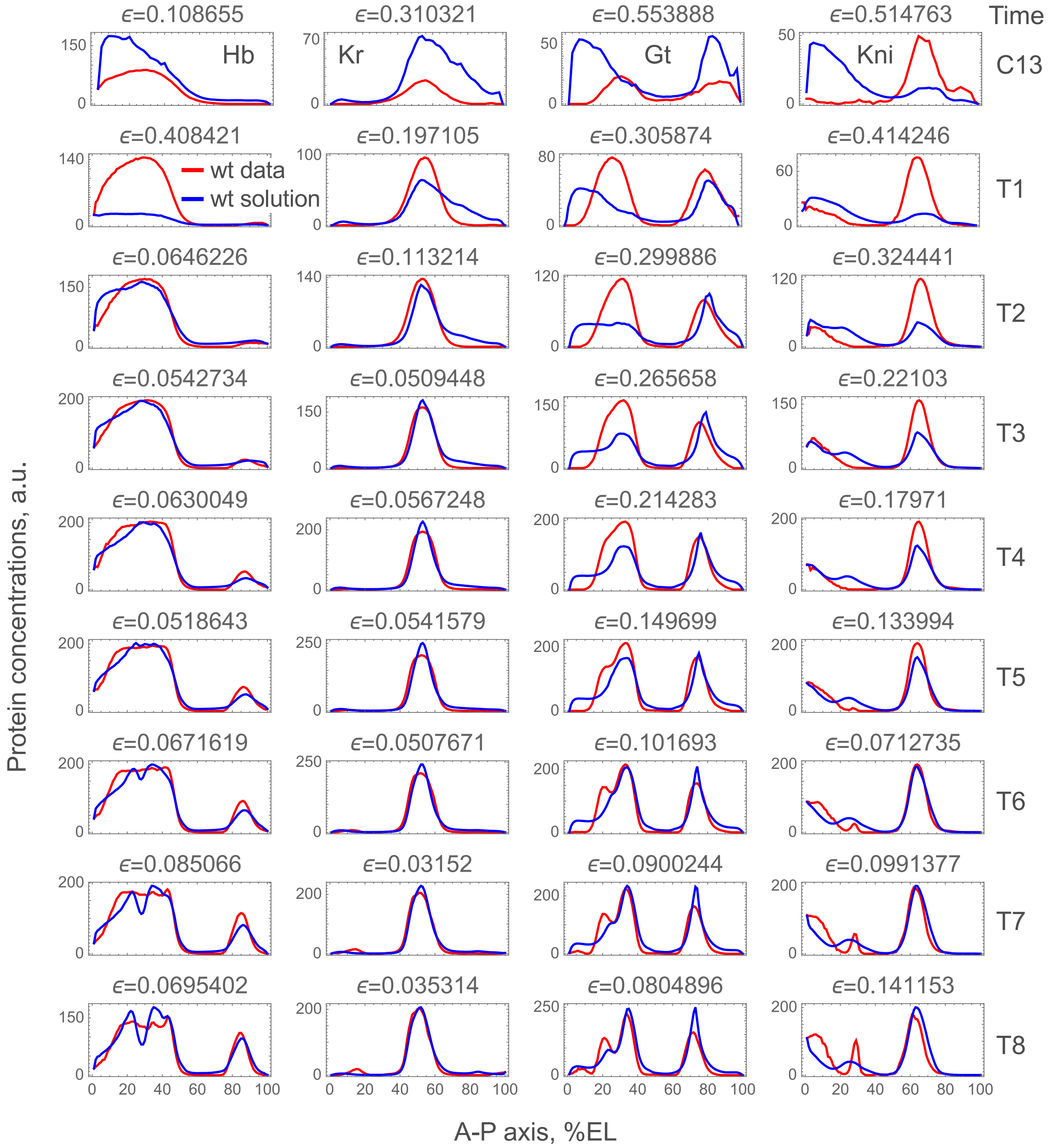

Figure 1: Wild-type expression patterns and the data approximation errors $\epsilon^a(t)$. The spatial profiles of protein concentrations in the expression data (red) and the model solution for the reference genome (blue) are shown for the gap genes *hb*, *Kr*, *gt*, and *kni* (columns from left to right, respectively), for nine time points (mid cleavage cycle 13 and eight time classes T1–T8 in cycle 14A; the time classification is according to Surkova et al. (2008)). The value of $\epsilon^a(t)$ from (3) is given for each gene a and time t. There are in total 36 (4 genes × 9 time points) spatial expression profiles and corresponding errors.

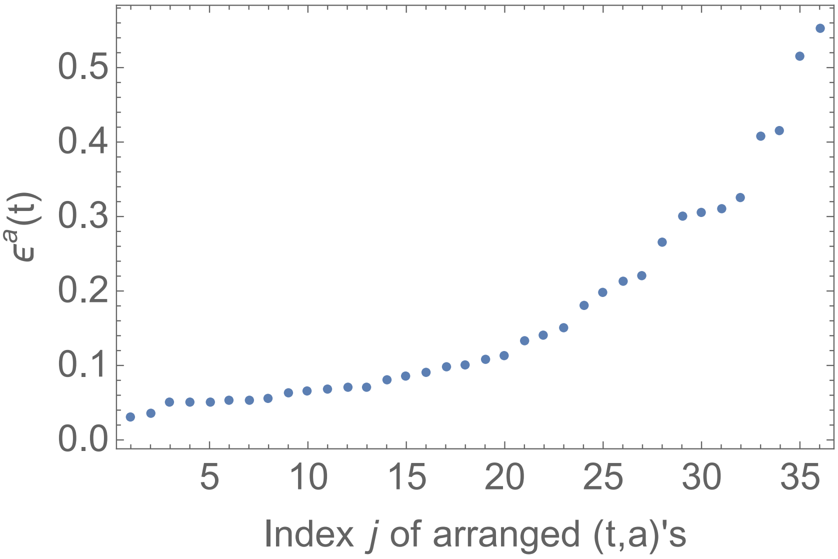

Figure 2: Arranged values of the data approximation errors $\epsilon^a(t)$.

Analysis of purifying selection with the new scores

We repeated the analysis of purifying selection from the main text using the new scores and for the case of comparison between the population genotypes and genotypes randomly mutated under the neutral site frequency spectrum. For each k, we calculated the p-value for the null hypothesis that the population genotypes and the randomly mutated genotypes provide the same variation in gap gene expression under the assumption that we quantify this variation by the score $w^{\text{new}}(k)$. Just as it was done for the full $wPGP$ score in the main text, this p-value was computed by comparing the median score for the population with the distribution of such median scores for 100 families of randomly mutated genotypes. The results show statistically significant evidence for action of purifying selection in the population for all new scores (Fig. 3). Even more, Fig. 3 demonstrates that the domain of small values of k, corresponding to higher precision of the model as described above, is associated with the same p-value as for the full score ($k = 36$). This result indicates the robustness of our predictions.

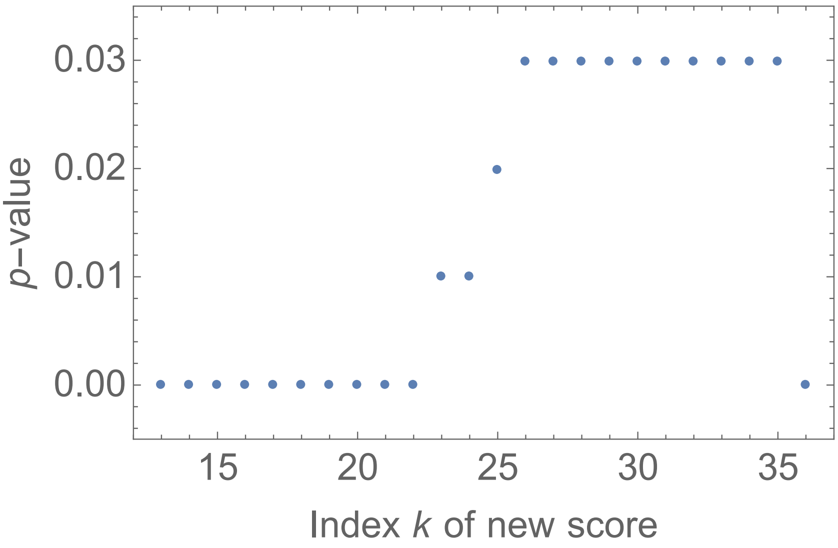

Figure 3: p-values of the test on purifying selection using 24 new scores.

References

Xin He, Duque, Thyago S. P. C., and Saurabh Sinha. Evolutionary Origins of Transcription Factor Binding Site Clusters. *Molecular biology and evolution*, 29(3):1059–1070, March 2012. doi: 10.1093/molbev/msr277.

M Kazemian, C Blatti, Adam Richards, Michael McCutchan, Noriko Wakabayashi-Ito, Ann S Hammonds, Susan E Celniker, Sudhir Kumar, Scot A Wolfe, Michael H Brodsky, and Saurabh Sinha. Quantitative analysis of the Drosophila segmentation regulatory network using pattern generating potentials. *PLoS biology*, 8(8):e1000456, 2010. doi: 10.1371/journal.pbio.1000456.

Konstantin Kozlov, Vitaly Gursky, Ivan Kulakovskiy, and Maria Samsonova. Sequence-based model of gap gene regulatory network. *BMC Genomics*, 15 Suppl 12:S6, December 2014. doi: 10.1186/1471-2164-15-S12-S6.

A. H. Samee and S. Sinha. Evaluating thermodynamic models of enhancer activity on cellular resolution gene expression data. *Methods*, 62:79–90, 2013.

Svetlana Surkova, David Kosman, K N Kozlov, Manu, Ekaterina Myasnikova, Anastasia A Samsonova, Alexander Spirov, Carlos E Vanario-Alonso, Maria G Samsonova, and John Reinitz. Characterization of the Drosophila segment determination morphome. *Developmental biology*, 313(2):844–862, 2008.
