# Supplementary material for: Translating natural genetic variation to gene expression in a computational model of the Drosophila gap gene regulatory network
Source: PLoS One. 2017 Sep 12;12(9):e0184657. doi: 10.1371/journal.pone.0184657 (PMC5595321; doi:10.1371/journal.pone.0184657)
Supplement: S1 Table — (PDF) [file pone.0184657.s004.pdf]

Table S1. Parameter values in the model.

	Hb	Kr	Gt	Kni	Bcd	Cad	Tll	Hkb
α/β for *hb*	2572	11	473	704	3821	1233	824*	5488
α/β for *Kr*	136	223*	311	60	33	9084*	3389	994*
α/β for *gt*	1528	4251	8310	1513	918	2272	3613	1209
α/β for *kni*	3947	2424	3400	8974	5233	11	5126	9176
K^a	0.0057	0.0049	0.0364	0.0499	0.0080	0.0056*	0.0002	0.0013
ω^a	5	4.96	5	1	1	1*	4.57	1.13*
τ^a	2	2.13	2	2	–	–	–	–
d_R^a	100	400	100	141	–	–	–	–
λ_u^a	1.14	1.38	3.07	1	–	–	–	–
λ_v^a	1.55	16.61	8.93	8.28	–	–	–	–
$q_{\rm BTM}$	1.0e−05	0.8e−05	0.5e−05	5.0e−05	–	–	–	–

The parameter values are as reported by Kozlov et al. (2014). The first four rows contain the activation efficiency parameters (α, shown in red) and the repression efficiency parameters (β, shown in blue) for each TF (in columns) and target gene (in rows). Each TF can be either activator or repressor for a given target gene. K^a is the affinity constant for the strongest binding site of each TF. ω^a is the cooperativity parameter. τ^a is the delay time (in minutes). d_R^a is the repression range (in basepairs) for the short-range repression mechanism. λ_u^a and λ_v^a are log2/halftime (in minutes^{-1}) for mRNA and protein, respectively. $q_{\rm BTM}$ is a factor by which Z_{ON} from Eq. (3) of the main text is multiplied. This parameter represents the basal level of the BTM-promotor interaction, so the statistical weights corresponding to all other ON-states are counted from that level (He et al., 2010). The parameters from the last five rows are only for the four gap genes. All parameter values in the table were found by the differential evolution optimization method minimizing the difference between the model output and the wild type gap gene expression data. The following additional parameters from the model equations were fixed during optimization: $R_u^a = 10$, $R_v^a = 30$, $D_u^a = 0.015$, and $D_v^a = 0.15$ for each gene a. The asterisks mark poorly identifiable parameters according to the identifiability analysis (Kozlov et al., 2014).

References

Xin He, Md. Abul Hassan Samee, Charles Blatti, and Saurabh Sinha. Thermodynamics-based models of transcriptional regulation by enhancers: The roles of synergistic activation, cooperative binding and short-range repression. *PLoS Computational Biology*, 6:e1000935, 2010.

Konstantin Kozlov, Vitaly Gursky, Ivan Kulakovskiy, and Maria Samsonova. Sequence-based model of gap gene regulatory network. *BMC Genomics*, 15 Suppl 12:S6, December 2014. doi: 10.1186/1471-2164-15-S12-S6.
