# Supplementary material for: Translating natural genetic variation to gene expression in a computational model of the Drosophila gap gene regulatory network
Source: PLoS One. 2017 Sep 12;12(9):e0184657. doi: 10.1371/journal.pone.0184657 (PMC5595321; doi:10.1371/journal.pone.0184657)
Supplement: S2 Table — The SNPs are arranged in the order of increasing genomic position. The first six columns provide the following information (from left to right): SNP index, gene, chromosome, genomic position (dm3 / BDGP5), reference and alternative nucleotides of the SNP, and SNP frequency in the study population. The next five columns present the following information about the model TFBSs containing these SNPs: TF, starting and ending positions of the binding site, reference sequence of the binding site, and its PWM-score. The table is divided into four parts, separately for each gap gene. (PDF) [file pone.0184657.s005.pdf]

#	Gene	Chrom	Position	Ref/Alt	Freq	TFBSs				
						TF	Start	End	Word	PWM-score
1	*gt*	X	2320021	T/C	0.127	Cad	2320014	2320024	TTTTTTTATT	5.058
						Cad	2320017	2320027	TTTTATTATA	5.586
						Hb	2320011	2320021	ATTTTTTTTT	3.336
						Hb	2320012	2320022	TTTTTTTTTA	2.905
						Hb	2320013	2320023	TTTTTTTTAT	3.983
						Hb	2320014	2320024	TTTTTTTATT	8.246
						Gt	2320012	2320024	TTTTTTTTTATT	4.366
						Gt	2320014	2320026	TTTTTTTATTAT	4.511
2	*gt*	X	2320035	T/G	0.051	Cad	2320026	2320036	ATTTATTATT	6.182
						Hb	2320026	2320036	ATTTATTATT	3.256
						Gt	2320031	2320043	TTATTATAGATC	4.308
3	*gt*	X	2320036	T/G	0.026	Cad	2320026	2320036	ATTTATTATT	6.182
						Hb	2320026	2320036	ATTTATTATT	3.256
						Gt	2320031	2320043	TTATTATAGATC	4.308
4	*gt*	X	2320037	A/G	0.026	Gt	2320031	2320043	TTATTATAGATC	4.308
5	*gt*	X	2320041	A/T	0.011	Gt	2320031	2320043	TTATTATAGATC	4.308
6	*gt*	X	2323038	C/G	0.134	Bcd	2323032	2323039	AAAATCC	4.076
						Hb	2323028	2323038	ACCGAAAATC	3.368
7	*gt*	X	2323129	C/G	0.019	Kr	2323127	2323138	CCAAAGGGCAA	5.406
						Tll	2323122	2323132	AAAGGCCAAA	6.825
8	*gt*	X	2324364	G/A	0.024	Hkb	2324359	2324369	TGCGGCGTGG	5.046
9	*gt*	X	2324393	T/G	0.019	Kni	2324389	2324402	TTGTTTCACTTTG	5.520
						Tll	2324392	2324402	TTTCACTTTG	4.538
10	*gt*	X	2324921	A/T	0.005	Hb	2324917	2324927	TGTAAAAACC	3.057
						Tll	2324920	2324930	AAAAACCAGA	5.134
11	*gt*	X	2325006	C/G	0.244	Cad	2325003	2325013	CGCCATAATG	4.534
12	*gt*	X	2325223	G/A	0.113	Cad	2325221	2325231	GGCCCTAAAA	5.009
13	*gt*	X	2325238	C/T	0.014	Kni	2325231	2325244	CCGCTGCAGTTTT	6.284
14	*gt*	X	2325239	A/T	0.373	Kni	2325231	2325244	CCGCTGCAGTTTT	6.284
15	*gt*	X	2325334	T/C	0.142	Cad	2325330	2325340	TTATATTGGT	4.949
						Hb	2325325	2325335	GATTTTTATA	3.719
						Hb	2325327	2325337	TTTTTATATT	4.815
16	*gt*	X	2325381	G/A	0.005	Kr	2325379	2325390	TGAAAGGGGTT	7.269
						Kr	2325380	2325391	GAAAGGGGTTT	7.609
17	*gt*	X	2325413	C/A	0.005	Kr	2325409	2325420	CCACCCGTCCA	4.659
18	*gt*	X	2325427	G/A	0.005	Kr	2325420	2325431	AAAAAGGGTGA	7.957
19	*gt*	X	2327069	A/G	0.464	Hb	2327063	2327073	AGCAAAAGAA	5.411
20	*gt*	X	2329021	T/A	0.005	Gt	2329012	2329024	ATGTTACGTTAC	7.616
						Gt	2329014	2329026	GTTACGTTACAT	4.940
21	*gt*	X	2329112	T/C	0.308	Cad	2329111	2329121	TGCAATAAAG	5.704
22	*gt*	X	2329129	C/A	0.014	Kni	2329116	2329129	TAAAGTAGAGCTC	6.437
23	*gt*	X	2329182	A/G	0.009	Hb	2329177	2329187	TTCGAAAAAC	3.289
						Gt	2329175	2329187	ATTTCGAAAAAC	4.155
24	*gt*	X	2329338	G/A	0.005	Hkb	2329329	2329339	GGAGGTGGGG	3.922
25	*gt*	X	2330586	C/A	0.009	Kni	2330584	2330597	GCGCTCAATTTTT	5.265
26	*gt*	X	2331029	A/T	0.225	Kni	2331028	2331041	AGAATCCGAGCAG	5.675
27	*gt*	X	2331091	T/A	0.009	Gt	2331082	2331094	TTGTTGGTTAAT	6.626
28	*gt*	X	2331288	C/T	0.272	Tll	2331282	2331292	TTCGACTTTG	6.545
29	*gt*	X	2332432	C/A	0.009	Kr	2332427	2332438	CAATCCCTTGC	4.946
30	*gt*	X	2332803	G/A	0.024	Kni	2332793	2332806	GGATTTAGCGCAA	4.875
						Hb	2332801	2332811	CGCAAACAGA	3.028
31	*gt*	X	2332929	G/A	0.005	Kni	2332923	2332936	GCATCGGGATCAG	4.846
32	*gt*	X	2333163	C/G	0.033	Bcd	2333157	2333164	GTAATCC	6.044
33	*gt*	X	2333974	T/C	0.352	Tll	2333971	2333981	TTTGATCTTT	5.398
34	*gt*	X	2334048	G/A	0.005	Kr	2334045	2334056	CCGAAGGATAA	4.786

#	Gene	Chrom	Position	Ref/Alt	Freq	TFBSs				
						TF	Start	End	Word	PWM-score
35	*hb*	3R	4520484	T/C	0.005	Kni	4520474	4520487	TTGATCCATTCTG	4.647
36	*hb*	3R	4522364	A/G	0.005	Cad	4522356	4522366	GGCAAAAAAC	4.800
						Kni	4522361	4522374	AAAACAAGTTCAC	6.057
						Hb	4522355	4522365	GGGCAAAAAA	3.423
						Hb	4522356	4522366	GGCAAAAAAC	8.497
37	*hb*	3R	4523422	G/T	0.005	Cad	4523415	4523425	TTTAATGGCA	5.382
38	*hb*	3R	4523654	A/G	0.014	Hb	4523647	4523657	AAGCAAAAAC	3.595
						Hb	4523648	4523658	AGCAAAAACA	5.729
						Gt	4523644	4523656	GCAAAGCAAAAA	3.865
39	*hb*	3R	4523696	C/T	0.005	Hkb	4523691	4523701	AGAGCCGTTA	4.367
40	*hb*	3R	4523824	A/G	0.005	Gt	4523820	4523832	CAAAAGCAATAA	3.954
41	*hb*	3R	4524787	C/A	0.009	Kni	4524776	4524789	AACAACAGAGCAA	5.714
						Hb	4524784	4524794	AGCAAACAAA	5.431
42	*hb*	3R	4524952	G/T	0.019	Bcd	4524945	4524952	GACTTAG	4.273
						Hb	4524950	4524960	AGCGAAGAAA	3.285
43	*hb*	3R	4524991	C/G	0.009	Hkb	4524989	4524999	GCCCGCCCAG	5.084
44	*hb*	3R	4525176	G/A	0.023	Bcd	4525169	4525176	CGATTAG	4.740
45	*hb*	3R	4525279	T/C	0.028	Hb	4525276	4525286	CATAACAAAT	3.095
						Gt	4525272	4525284	AGGACATAACAA	4.366
46	*hb*	3R	4525771	C/T	0.005	Hb	4525763	4525773	TTTCTTTCCT	4.496
47	*hb*	3R	4526184	T/C	0.005	Hb	4526174	4526184	ACCAAACAAT	3.099
48	*hb*	3R	4526441	G/A	0.029	Hkb	4526436	4526446	ACGGGCGTTC	5.548
49	*hb*	3R	4526793	A/C	0.088	Gt	4526792	4526804	AAGACACACAAA	4.508
50	*hb*	3R	4526924	C/T	0.019	Kr	4526919	4526930	CATCCCATTTC	4.485
51	*hb*	3R	4526964	G/A	0.009	Cad	4526957	4526967	TTTTATGACC	8.683
						Hb	4526954	4526964	GTTTTTTATG	8.489
						Hb	4526956	4526966	TTTTTATGAC	3.980
52	*hb*	3R	4527104	T/G	0.009	Hb	4527100	4527110	GTTTTCTCCT	3.743
						Gt	4527093	4527105	GCTTTGCGTTTT	4.353
53	*hb*	3R	4527146	C/G	0.048	Hb	4527145	4527155	CTTCTTTATG	4.247
						Tll	4527140	4527150	ATTGTCTTCT	4.960
54	*hb*	3R	4531424	G/T	0.009	Hb	4531414	4531424	TTTGTTTAAG	4.032
55	*hb*	3R	4531521	G/A	0.024	Gt	4531519	4531531	TGTTTGTGTGTT	4.075
56	*hb*	3R	4531528	T/A	0.005	Gt	4531519	4531531	TGTTTGTGTGTT	4.075
57	*hb*	3R	4531588	G/T	0.009	Kni	4531585	4531598	TTGGTCTAATATA	4.462

#	Gene	Chrom	Position	Ref/Alt	Freq	TFBSs				
						TF	Start	End	Word	PWM-score
58	*kni*	3L	20688664	G/T	0.196	Hkb	20688659	20688669	GCACGCACAC	5.016
59	*kni*	3L	20688755	C/A	0.005	Kr	20688753	20688764	TCACTCATTCA	4.681
60	*kni*	3L	20688778	G/A	0.067	Hkb	20688768	20688778	TCACGCTCAG	4.613
61	*kni*	3L	20688857	T/G	0.005	Hb	20688852	20688862	TTTATTTATT	4.591
62	*kni*	3L	20688962	A/T	0.005	Hb	20688954	20688964	CATTTTTAAC	3.411
63	*kni*	3L	20688981	G/C	0.014	Cad	20688973	20688983	TTTTTTTGGC	5.701
						Cad	20688974	20688984	TTTTTTGGCA	5.099
						Hb	20688972	20688982	ATTTTTTTGG	3.112
						Hb	20688973	20688983	TTTTTTTGGC	7.849
						Hb	20688974	20688984	TTTTTTGGCA	3.003
						Gt	20688973	20688985	TTTTTTTGGCAT	4.100
64	*kni*	3L	20689895	A/T	0.005	Cad	20689887	20689897	TATTATAAAA	4.688
						Gt	20689888	20689900	ATTATAAAATAT	4.617
						Gt	20689893	20689905	AAAATATAAAAC	4.680
65	*kni*	3L	20689965	C/T	0.120	Tll	20689959	20689969	GCTGACTTCC	5.853
66	*kni*	3L	20690032	G/T	0.143	Kni	20690025	20690038	AAAACGGGAAAAA	5.129
						Kr	20690025	20690036	AAAACGGGAAA	4.566
						Hb	20690029	20690039	CGGGAAAAAA	5.658
						Hb	20690030	20690040	GGGAAAAAAC	7.286
67	*kni*	3L	20690657	A/T	0.019	Kni	20690650	20690663	CAAACAAGAGCAC	8.372
						Hb	20690648	20690658	GCCAAACAAG	3.265
68	*kni*	3L	20690799	C/T	0.005	Tll	20690792	20690802	AAAAGTCAAT	8.990
69	*kni*	3L	20692902	C/A	0.009	Kni	20692891	20692904	GGAAATGCGGCAA	4.444
70	*kni*	3L	20693004	G/T	0.005	Hb	20693000	20693010	CTTGTTTGAT	3.245
71	*kni*	3L	20693120	T/A	0.009	Bcd	20693118	20693125	CTAATGC	4.109

#	Gene	Chrom	Position	Ref/Alt	Freq	TFBSs				
						TF	Start	End	Word	PWM-score
72	*Kr*	2R	21106810	C/A	0.01	Kni	21106809	21106822	CTGATTTATTTGT	4.459
						Tll	21106808	21106818	TCTGATTTAT	4.392
73	*Kr*	2R	21106823	G/A	0.387	Hb	21106813	21106823	TTTATTTGTG	5.456
						Hb	21106820	21106830	GTGTTTTATG	4.741
						Hb	21106822	21106832	GTTTTATGTC	4.885
74	*Kr*	2R	21106902	T/A	0.024	Hb	21106900	21106910	GTGGAAAAAA	3.776
						Hb	21106901	21106911	TGGAAAAAAC	5.654
75	*Kr*	2R	21110483	C/G	0.271	Kr	21110476	21110487	AAATCCCTCTG	4.515
76	*Kr*	2R	21110775	T/C	0.051	Tll	21110769	21110779	AAATGTCAAA	5.566
77	*Kr*	2R	21110799	T/G	0.348	Hb	21110789	21110799	TGCAAAAAAT	5.314
78	*Kr*	2R	21112768	T/C	0.343	Hb	21112762	21112772	TTGTTTTGGT	4.520
79	*Kr*	2R	21112791	T/A	0.207	Tll	21112785	21112795	TGAAGTCGAA	4.631
80	*Kr*	2R	21113102	T/C	0.009	Cad	21113098	21113108	ATTTTTTATT	4.306
						Cad	21113101	21113111	TTTTATTATA	5.586
						Hb	21113098	21113108	ATTTTTTATT	6.628
81	*Kr*	2R	21113108	T/G	0.317	Cad	21113098	21113108	ATTTTTTATT	4.306
						Cad	21113101	21113111	TTTTATTATA	5.586
						Hb	21113098	21113108	ATTTTTTATT	6.628
						Gt	21113103	21113115	TTATTATATTTC	5.124
82	*Kr*	2R	21113130	C/T	0.005	Kr	21113125	21113136	GAATCCGTTTT	5.116
83	*Kr*	2R	21113139	A/G	0.024	Cad	21113131	21113141	GTTTTTTACT	4.234
						Hb	21113131	21113141	GTTTTTTACT	8.361
84	*Kr*	2R	21113279	C/T	0.023	Cad	21113269	21113279	GGTAAAAAAC	4.696
						Hb	21113269	21113279	GGTAAAAAAC	7.942
85	*Kr*	2R	21113423	C/A	0.009	Kni	21113416	21113429	CTGTTTCATTTTC	6.024
86	*Kr*	2R	21113555	C/T	0.005	Hb	21113552	21113562	CTCTAAAAAC	3.931
87	*Kr*	2R	21113582	G/T	0.005	Hb	21113572	21113582	TCTTTATGTG	2.968
						Gt	21113577	21113589	ATGTGGTGTAAT	4.209
						Gt	21113579	21113591	GTGGTGTAATAT	4.611
88	*Kr*	2R	21113686	C/T	0.390	Kni	21113680	21113693	TAGTTCTGATTTT	5.310
						Tll	21113684	21113694	TCTGATTTTT	6.239
89	*Kr*	2R	21113857	A/C	0.005	Kni	21113852	21113865	TCGCACCAATTTG	6.066
90	*Kr*	2R	21113974	T/A	0.005	Hb	21113972	21113982	GTTTTTCGCT	6.493
						Gt	21113972	21113984	GTTTTTCGCTTT	3.898
