# Supplementary figures and images for: Translating natural genetic variation to gene expression in a computational model of the Drosophila gap gene regulatory network
Source: PLoS One. 2017 Sep 12;12(9):e0184657. doi: 10.1371/journal.pone.0184657 (PMC5595321; doi:10.1371/journal.pone.0184657)

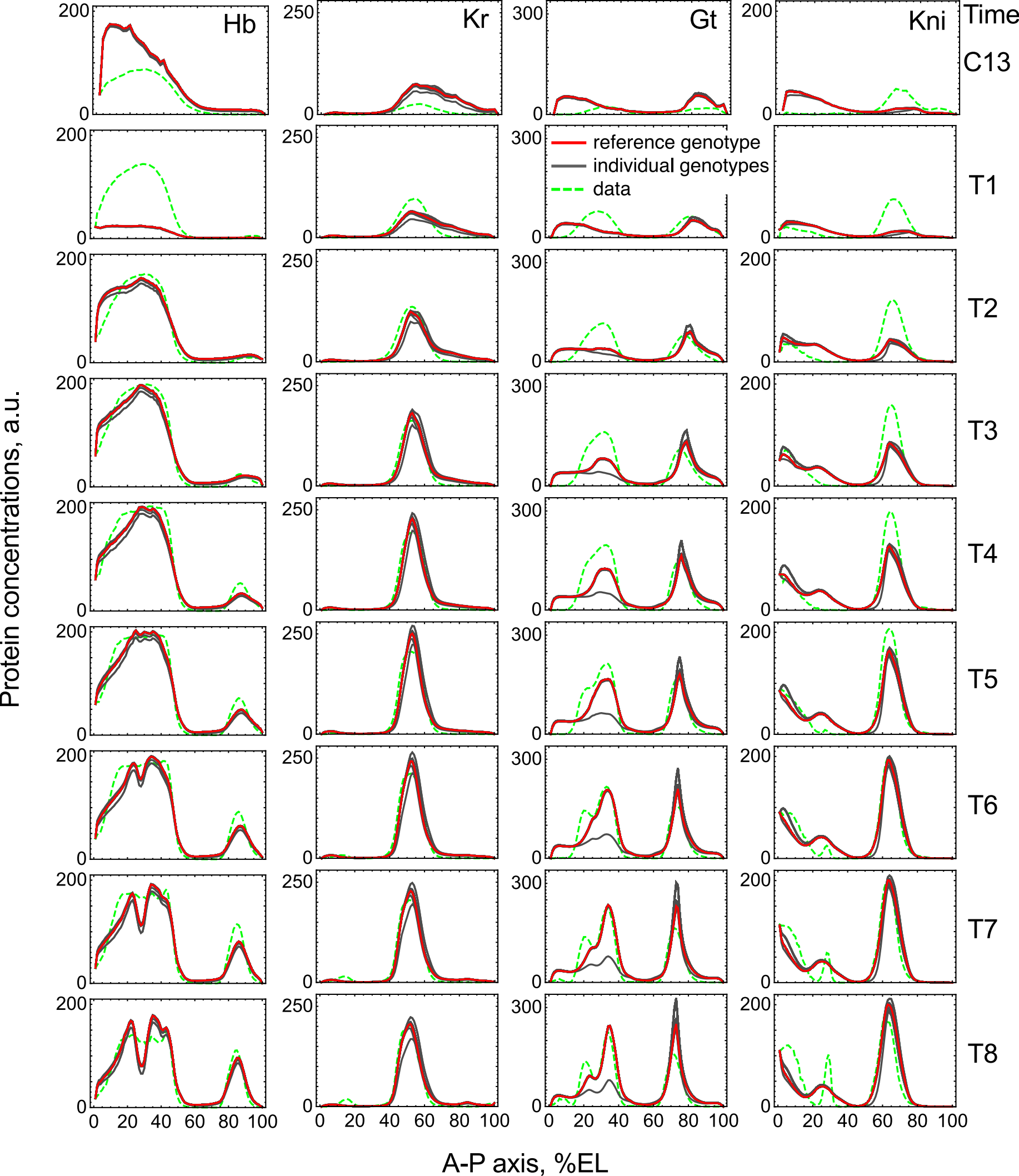

Supplement: S1 Fig — The figure shows expression patterns (model solutions) at 9 time points for 213 individual genotypes and for the reference genotype, together with the observed expression patterns. The time points include the mid cleavage cycle 13 and eight time classes T1–T8 in cleavage cycle 14A, as described in Methods. (PDF) [file pone.0184657.s006.pdf]

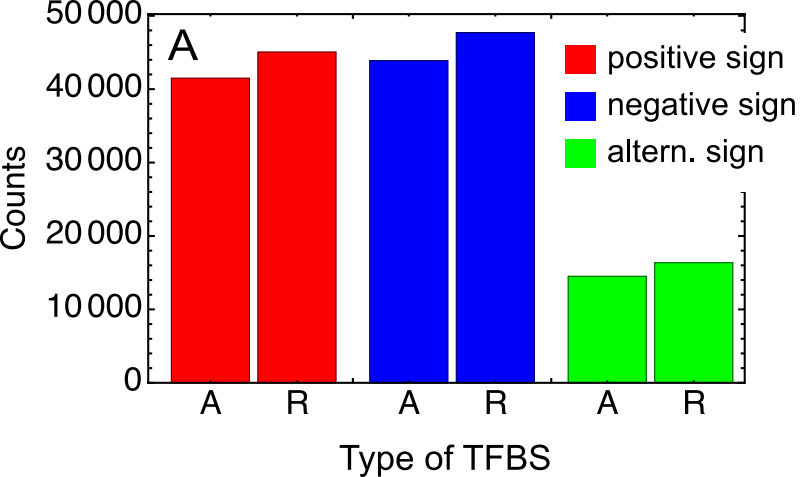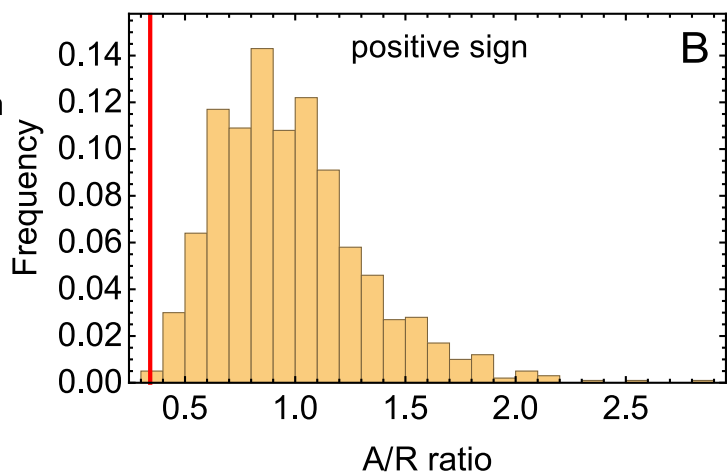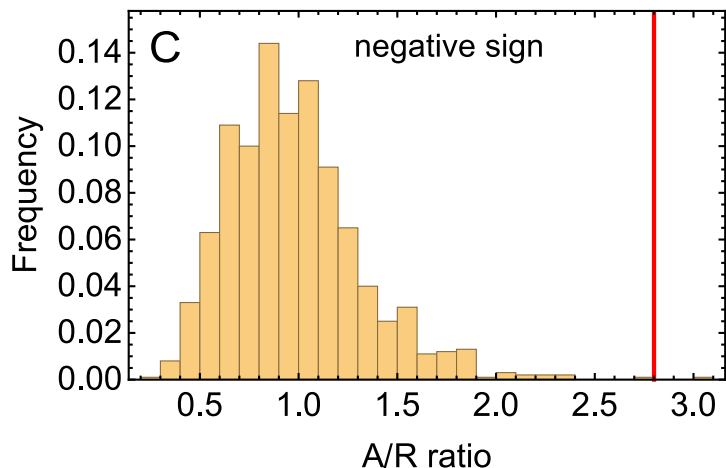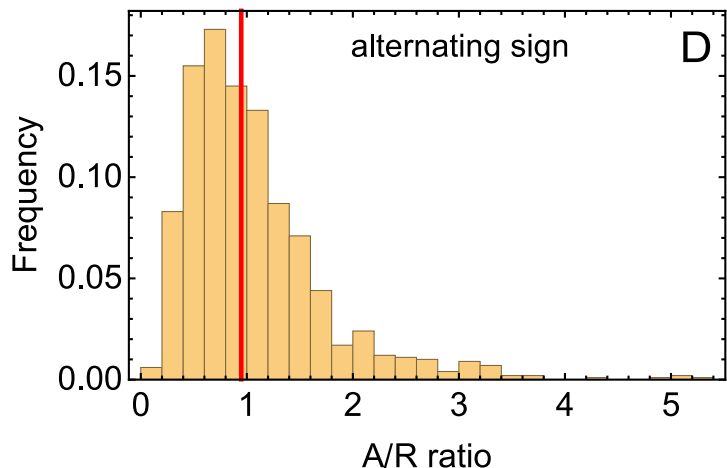

Supplement: S2 Fig — (A) Results are shown for 1000 resampling experiments. For each experiment, a set of positions was randomly sampled within the model TFBSs, and each position was randomly assigned to one of the three groups of putative sign of SNP influence: 36 positions for the group of positive sign, 38 for negative sign, and 13 for alternating sign. The regulatory roles (activator or repressor) of all TFBSs containing these positions were recorded and their total number was counted for each sign group. These numbers are shown in the panel. (B)–(D) For the sampled positions from each experiment and each sign group, the ratio of the total number of activating TFBSs to the total number of repressing TFBSs was calculated. The distribution of 1000 ratio values is shown for each sign group, together with the population ratio values (red lines). The one-tailed p-values were estimated from these panels: p = 0.001 both for the positive and for the negative sign groups, and p = 0.45 is the minimal value of the two possible one-sided values for the group of alternating sign. (PDF) [file pone.0184657.s007.pdf]

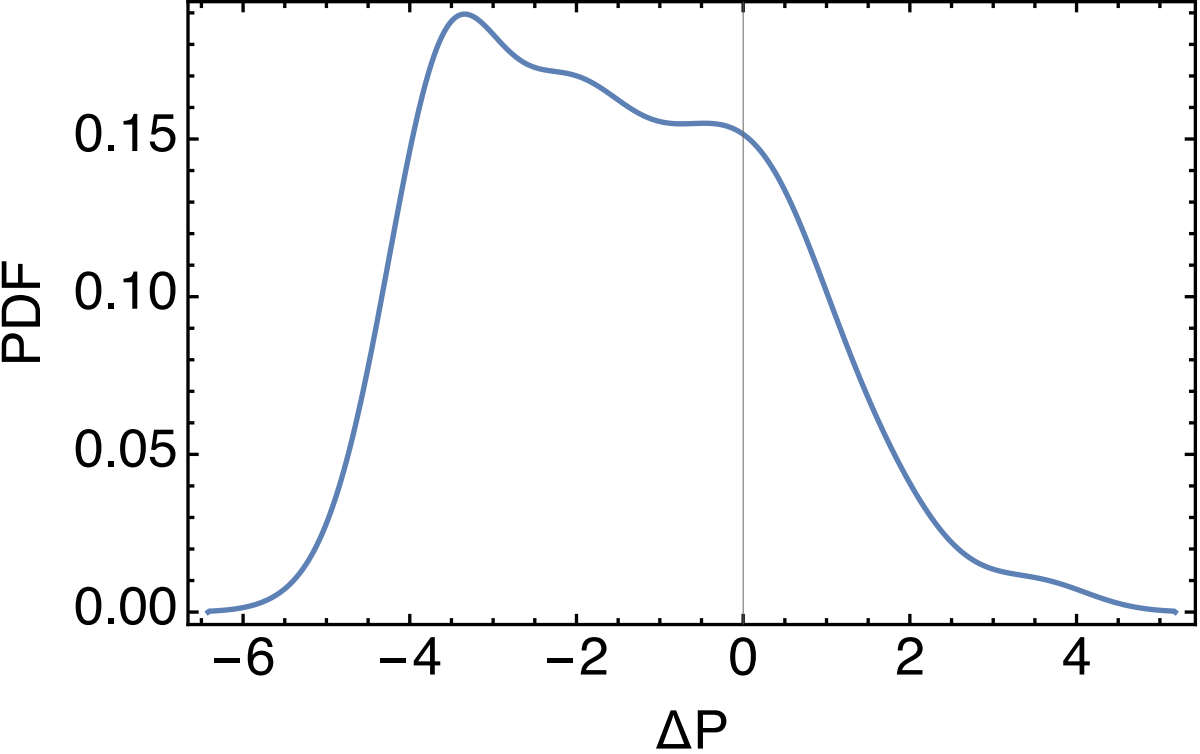

Supplement: S3 Fig — The probability density function is shown for ΔP=Ps-Psref, where Ps is the PWM score of binding site s containing a SNP, and Psref is the same score for the reference state of this site. (PDF) [file pone.0184657.s008.pdf]

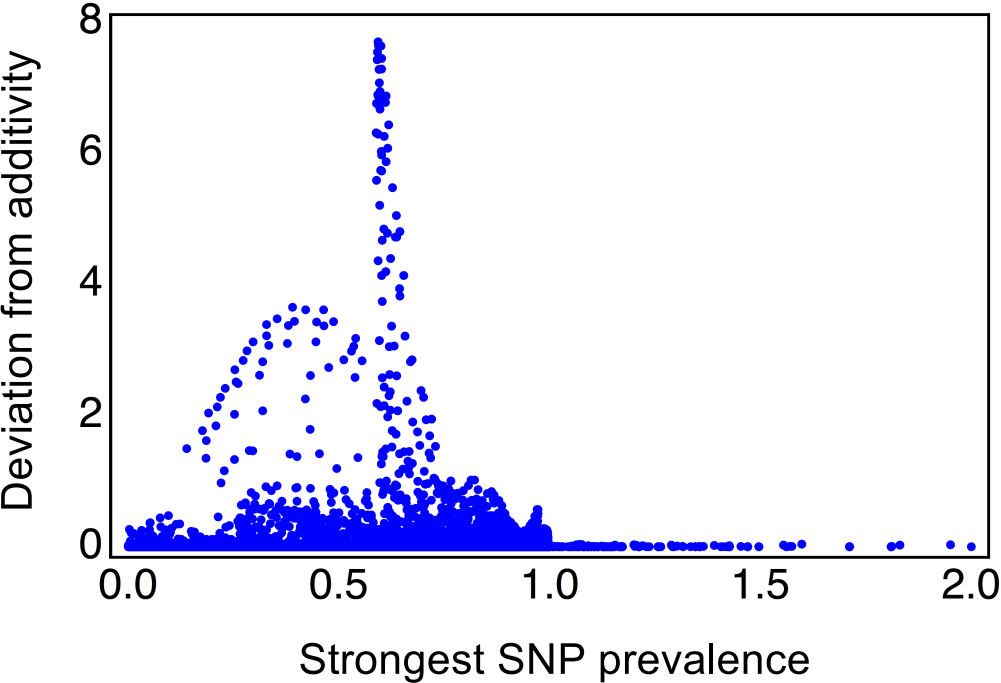

Supplement: S5 Fig — The vertical axis shows the difference |Δvgenk-∑SNPΔvSNPk|, and the horizontal axis shows the ratio (|ΔvSNPmaxk|-∑SNPi|ΔvSNPik|)/|ΔvSNPmaxk|, where SNPmax is the strongest SNP in a given regulatory region and SNPi are all other SNPs from the same regulatory region. All designations are introduced in the part of the main text related with Fig 5. (PDF) [file pone.0184657.s010.pdf]

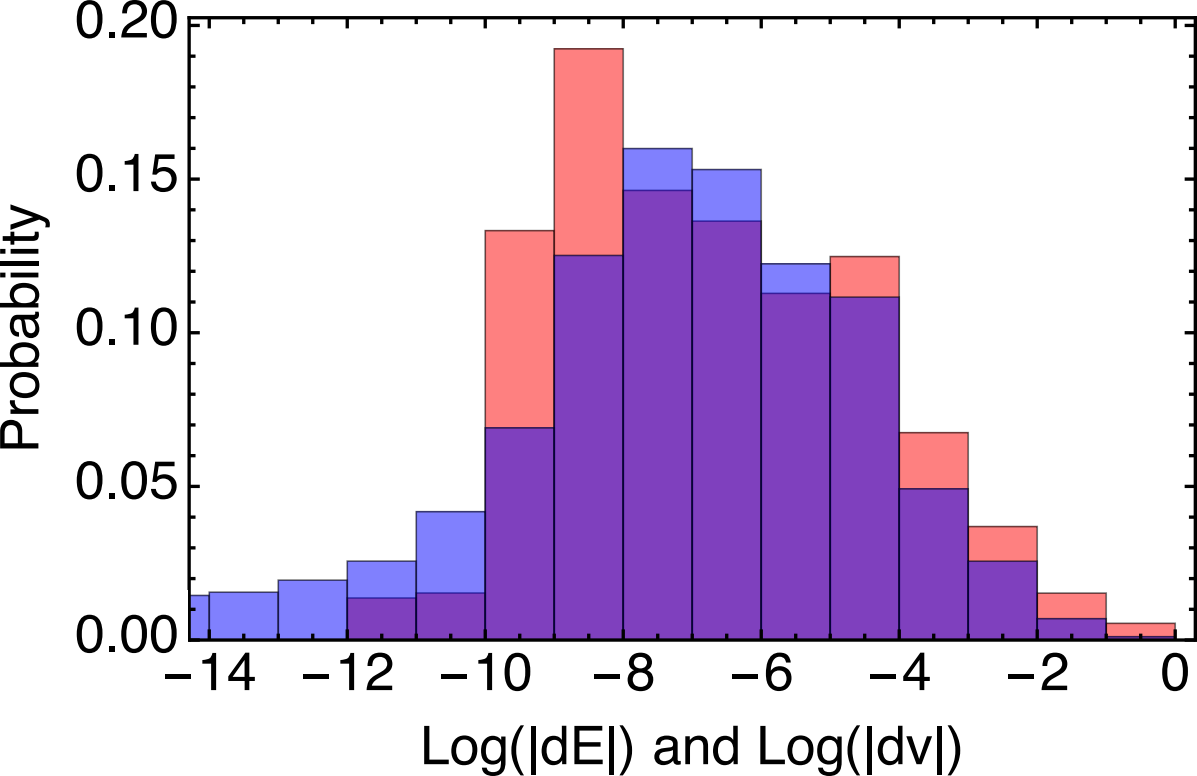

Supplement: S6 Fig — The scores are normalized by the maximal values. Only non-zero scores are considered. (PDF) [file pone.0184657.s011.pdf]

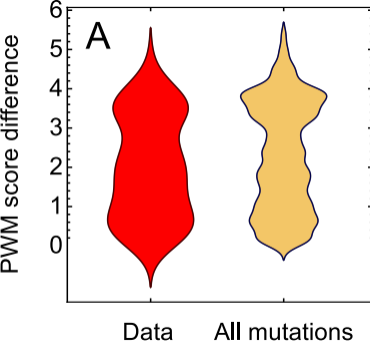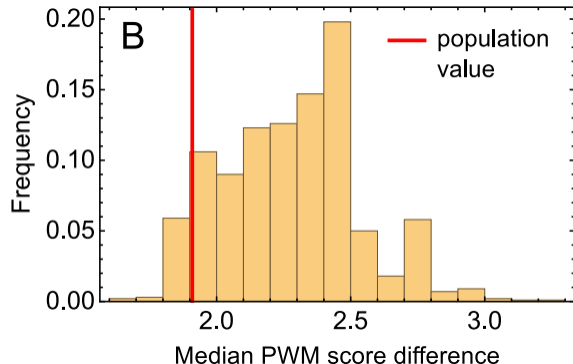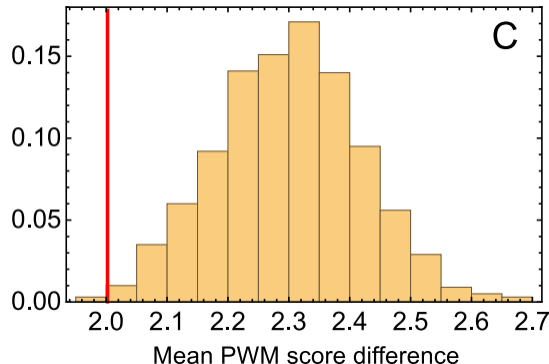

Supplement: S7 Fig — (A) The distribution chart for the binding score wb from Eq (9) calculated for SNPs observed in the population (red) and for all possible single mutations within the model TFBSs (yellow). The p-value for the comparison of the two distributions according to the bootstrap chi-square test: p = 0.27. (B) Comparison of the median wb for the population SNPs (red) and the medians for 1000 sets randomly sampled from the all-mutations distribution (histogram); each sampled set has the same length as the data set. The one-tailed p-value: p = 0.09. (C) The same as in B, but for the means instead of medians (p = 0.003). (PDF) [file pone.0184657.s012.pdf]

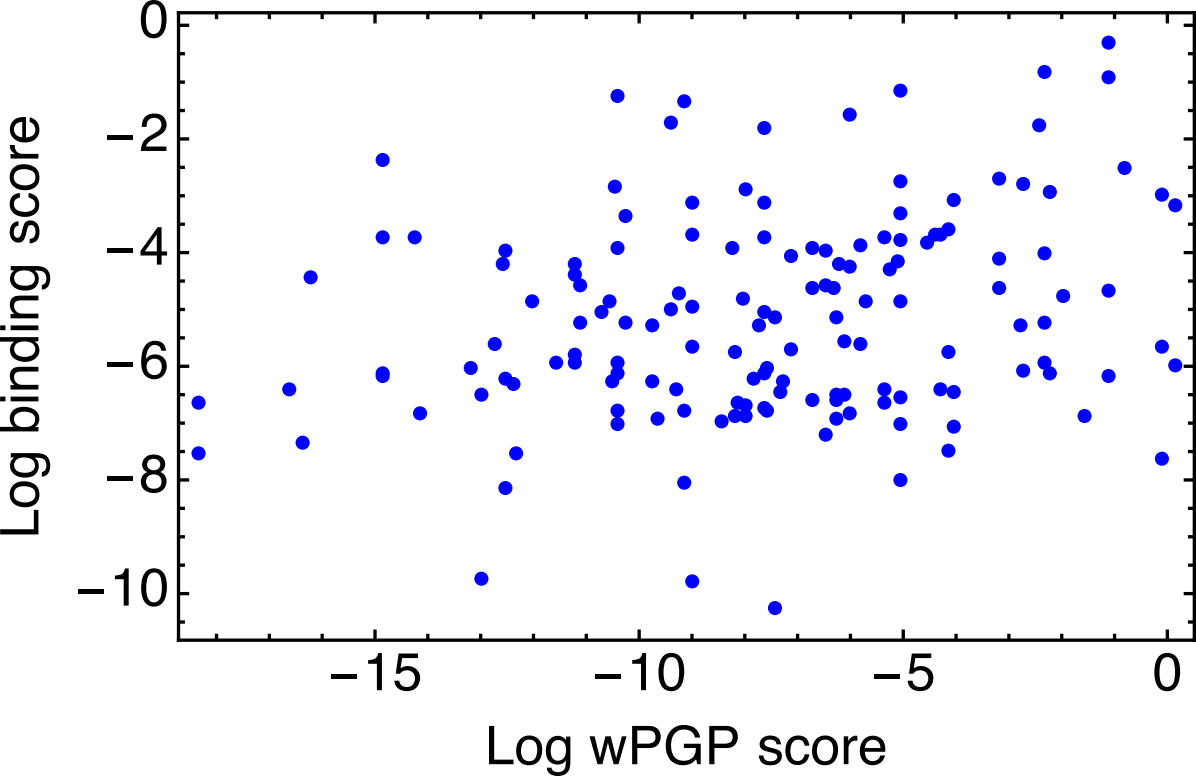

Supplement: S8 Fig — (PDF) [file pone.0184657.s013.pdf]

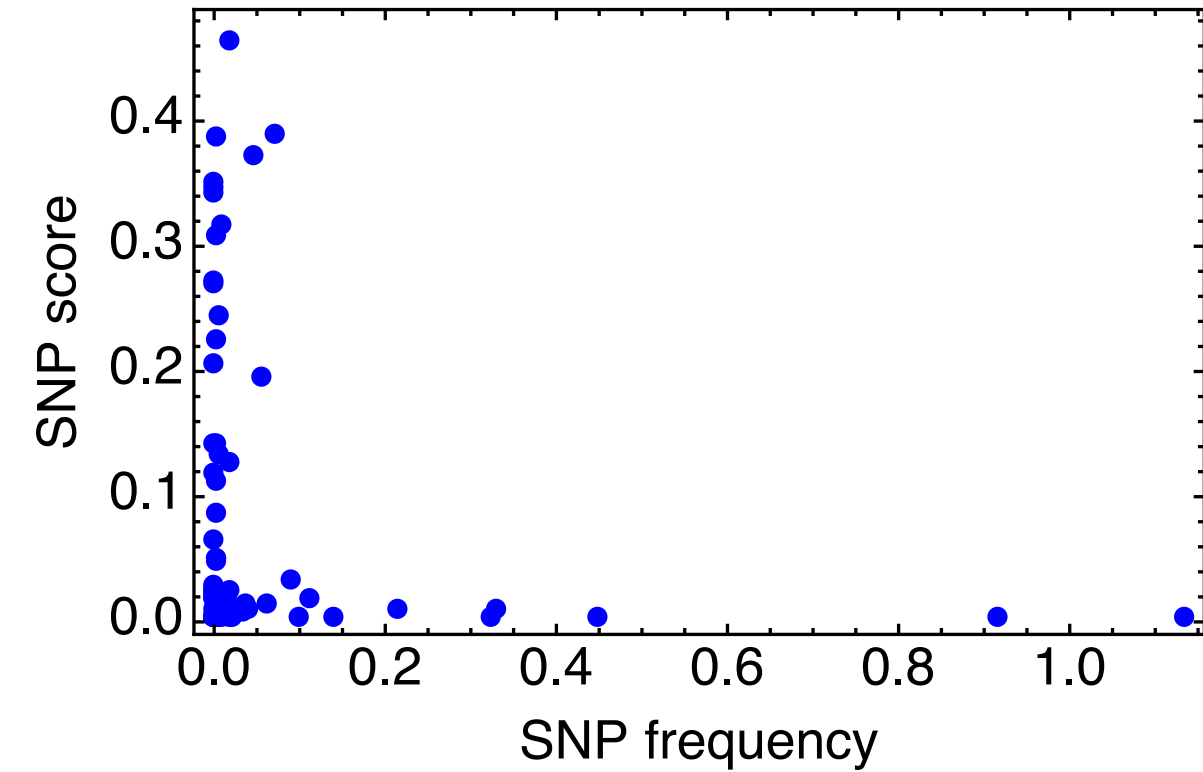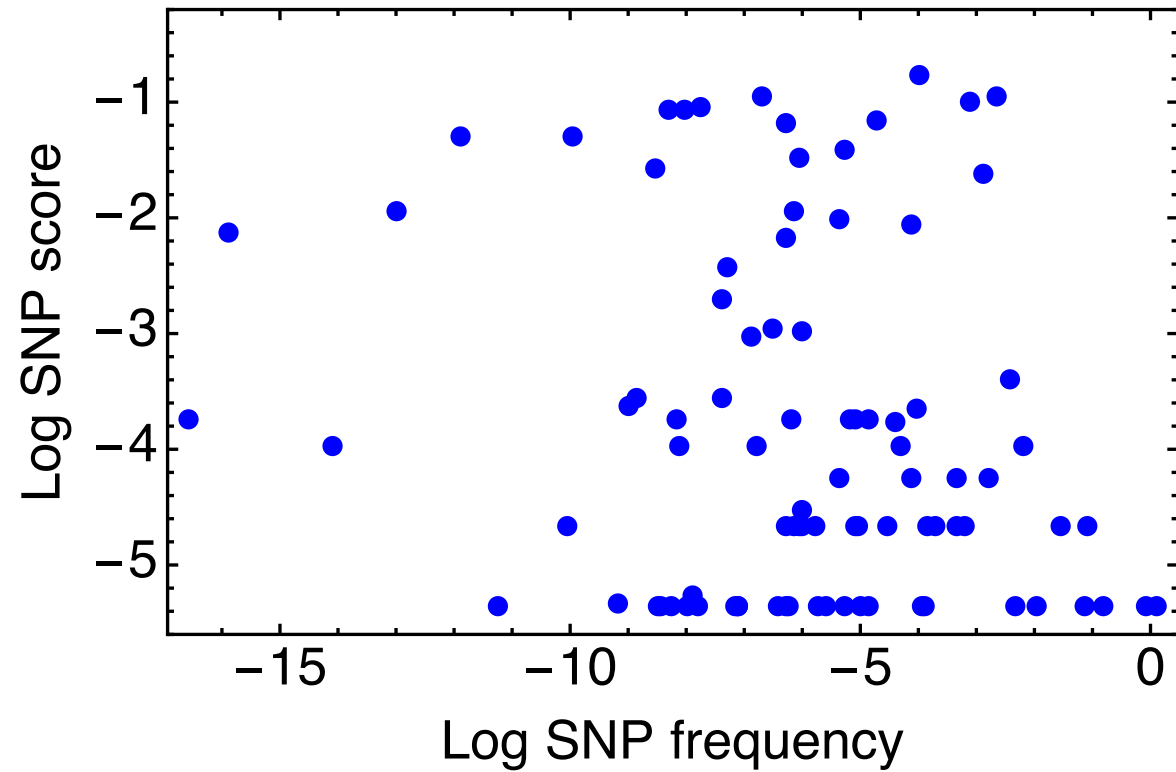

Supplement: S9 Fig — (PDF) [file pone.0184657.s014.pdf]

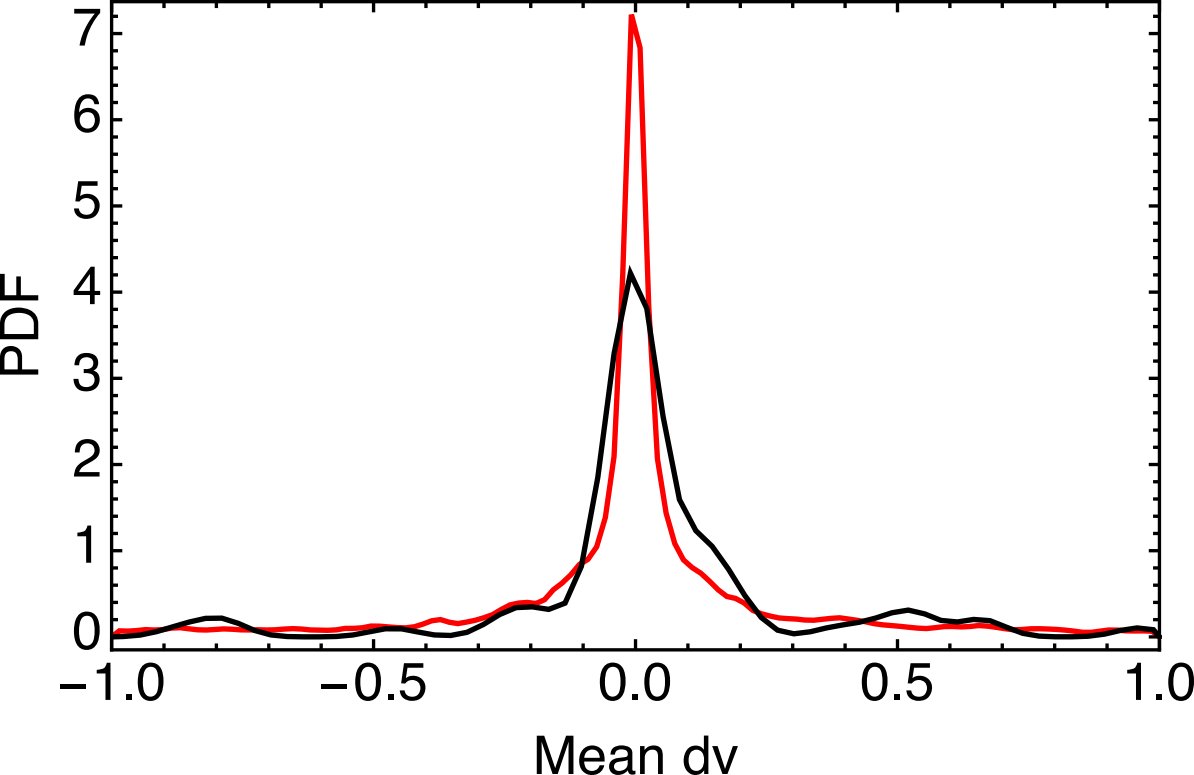

Supplement: S10 Fig — PDF stands for the probability density function. Long tails are not included in the scope of the figure. (PDF) [file pone.0184657.s015.pdf]

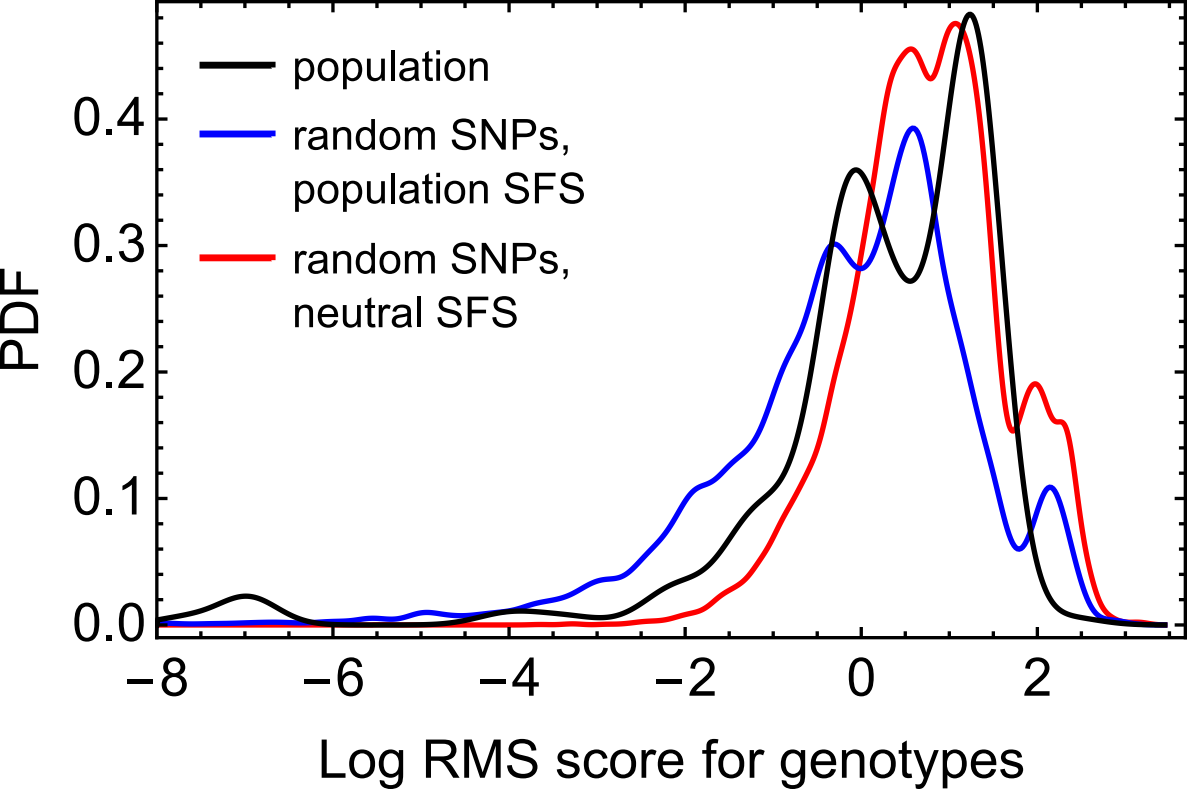

Supplement: S11 Fig — (PDF) [file pone.0184657.s016.pdf]

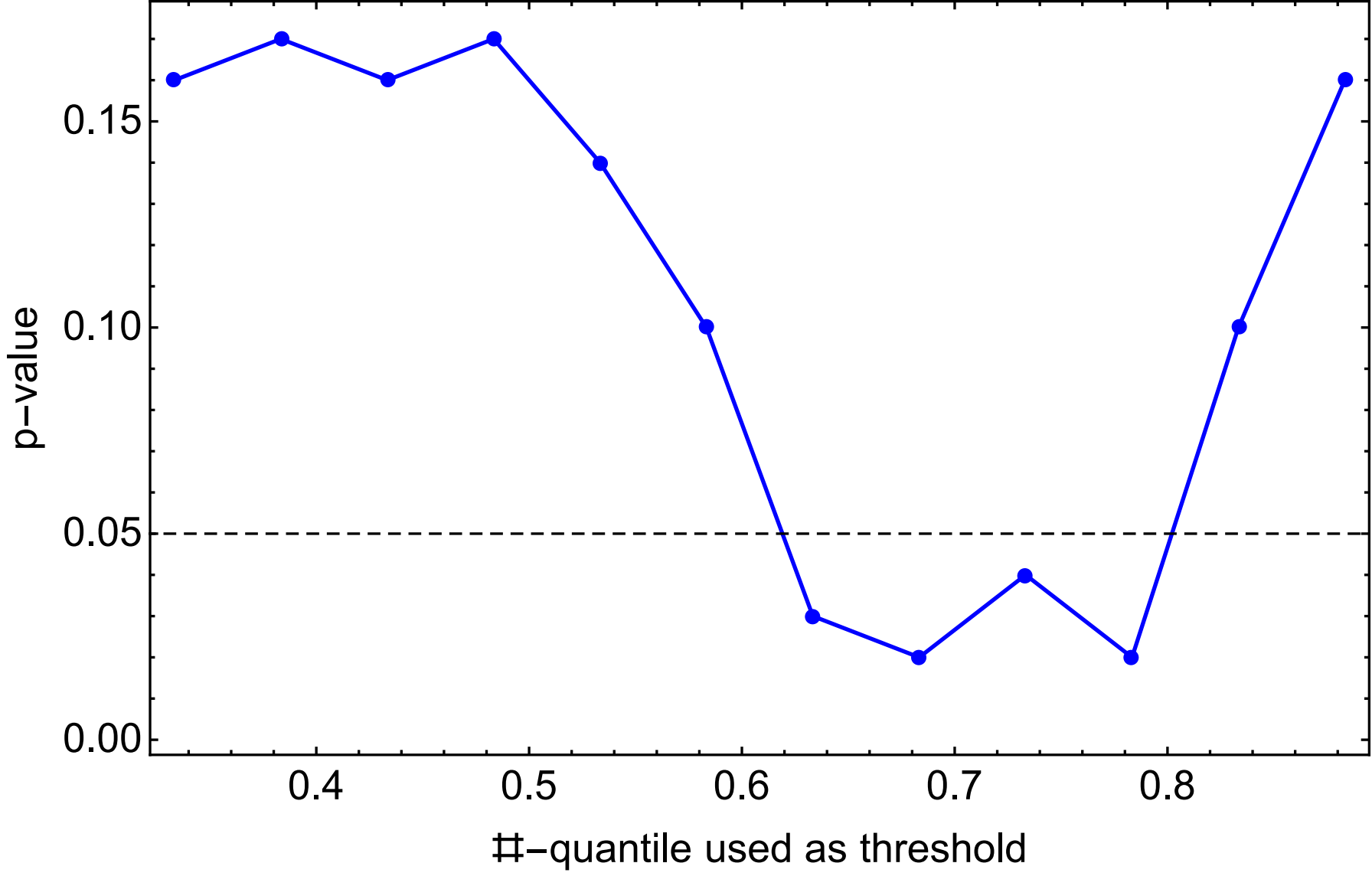

Supplement: S12 Fig — The threshold values were chosen as various quantiles of the wPGP score distribution for the population genotypes (black curve in Fig 7D). (PDF) [file pone.0184657.s017.pdf]

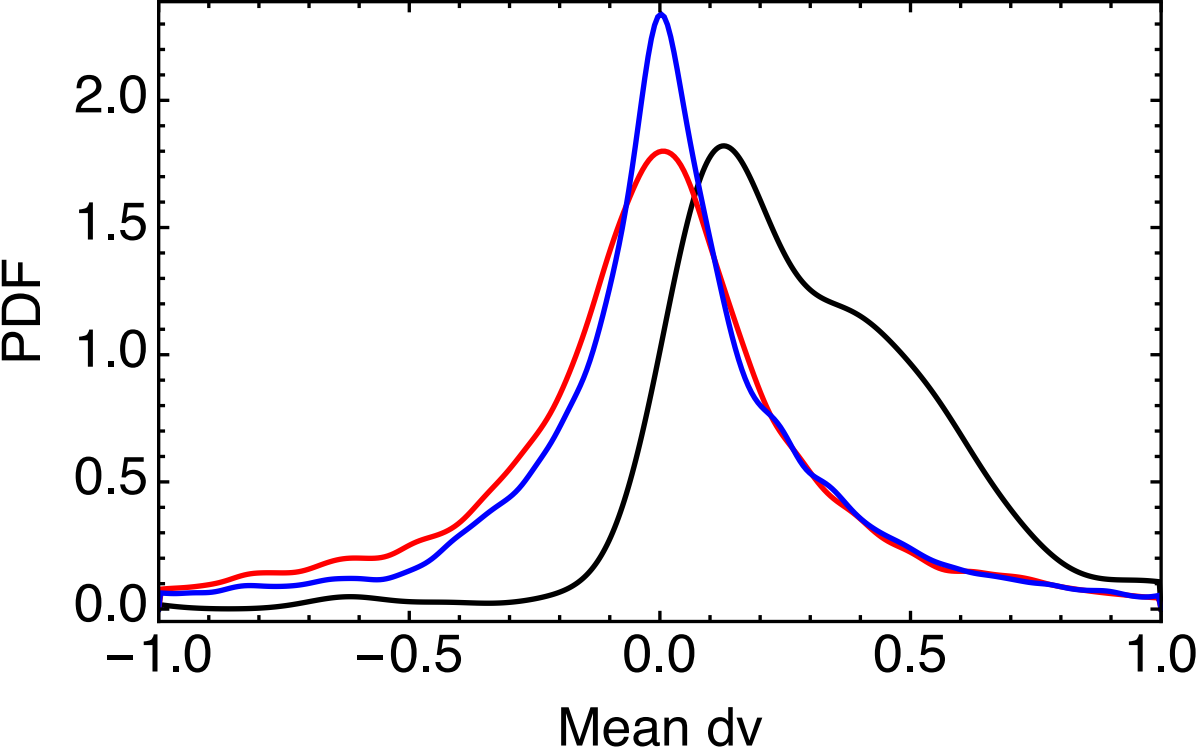

Supplement: S13 Fig — PDF stands for the probability density function. The difference between each pair of the distributions is statistically significant based on the bootstrap KS test (p < 0.0001). (PDF) [file pone.0184657.s018.pdf]

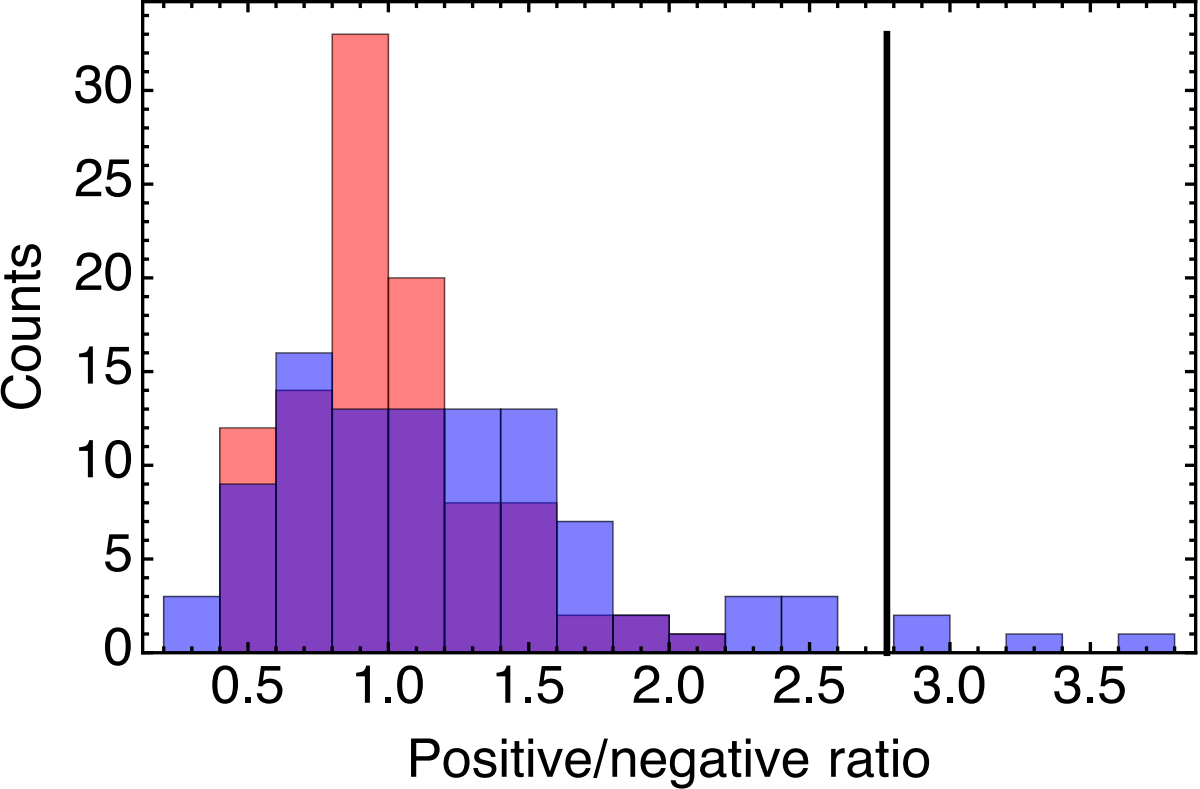

Supplement: S14 Fig — The distributions are shown for the neutral SFS (red) and population-derived SFS (blue). The means 〈Δv〉 were calculated for each SNP inside each genotype in a family, and the ratio was then computed for this family. The one-tailed p-values: p < 0.01 for the neutral SFS and p = 0.04 for the population-derived SFS. (PDF) [file pone.0184657.s019.pdf]

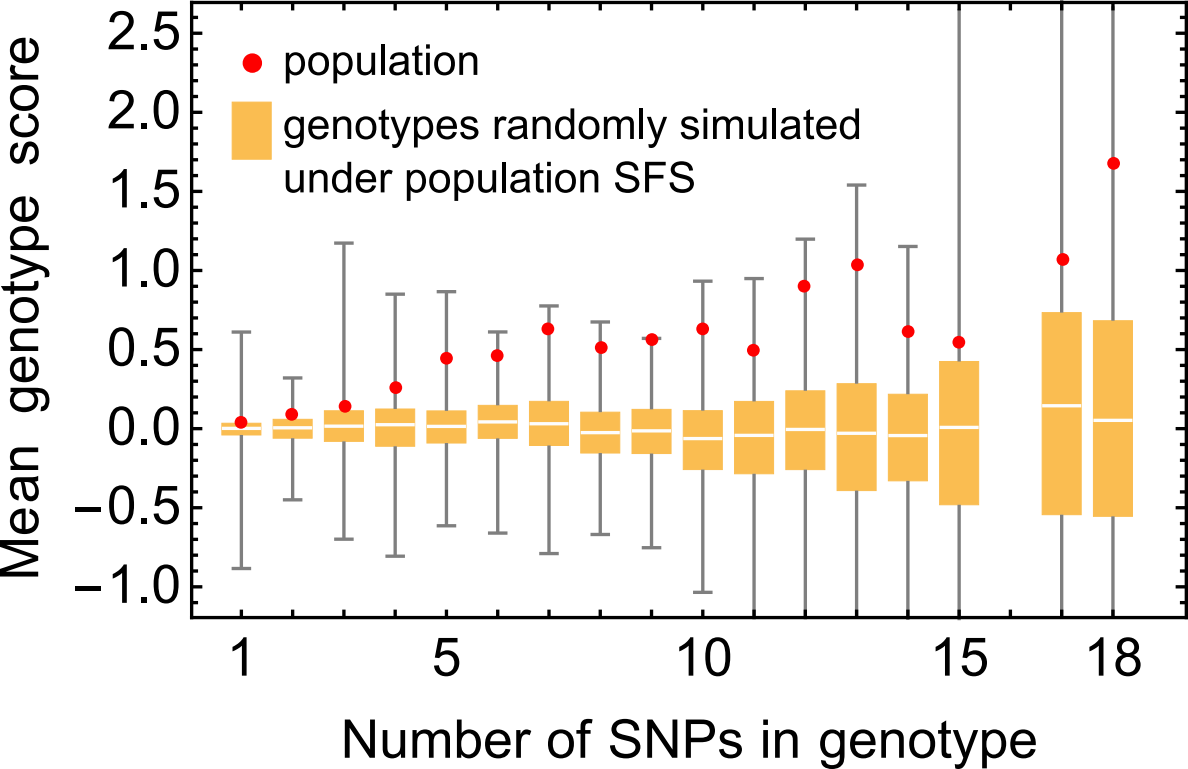

Supplement: S15 Fig — (PDF) [file pone.0184657.s020.pdf]

Protein conc., a.u.

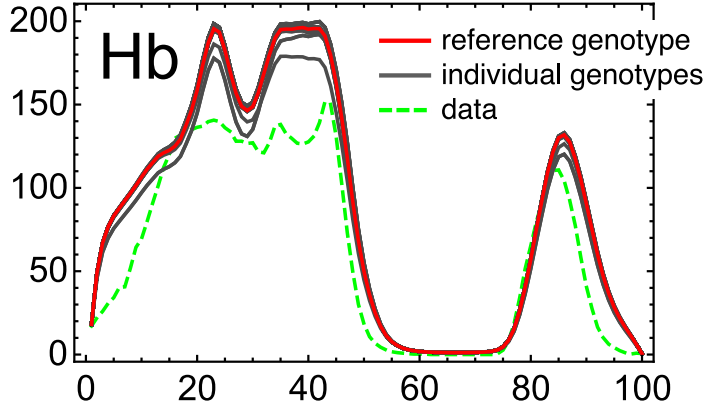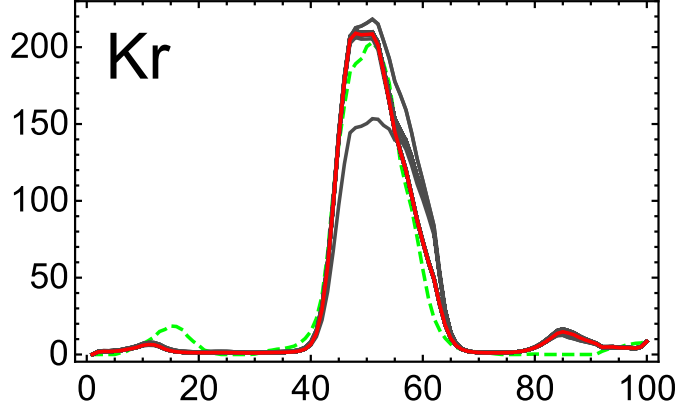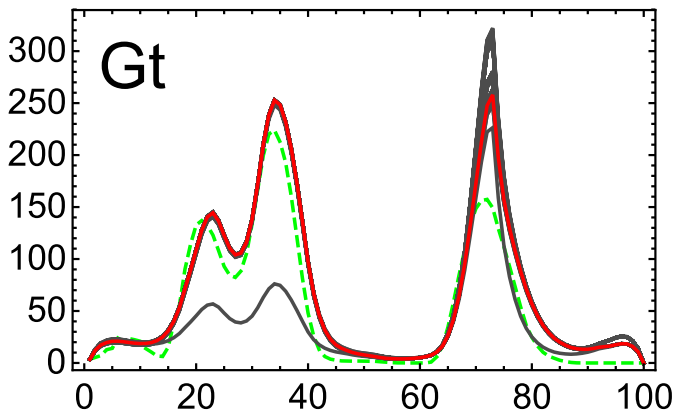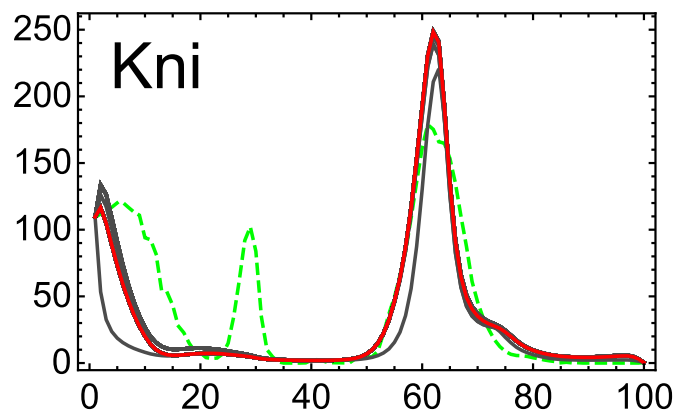

A-P axis, %EL

Supplement: S16 Fig — The spatial profiles of protein concentrations are shown at the end of cleavage cycle 14A for 213 individual genotypes and for the reference genotype, together with the observed expression patterns. (PDF) [file pone.0184657.s021.pdf]

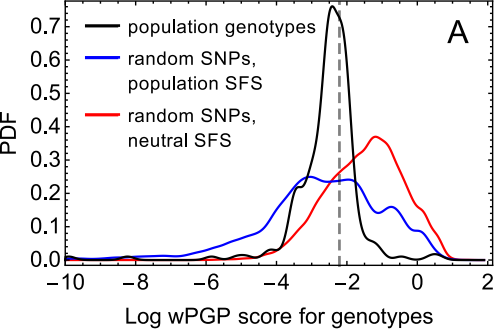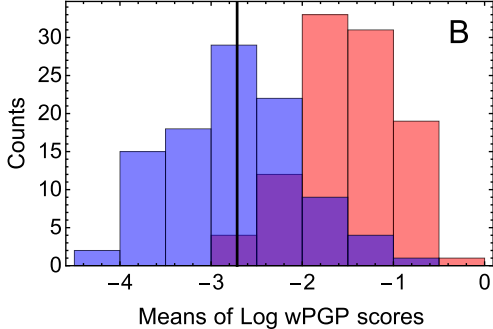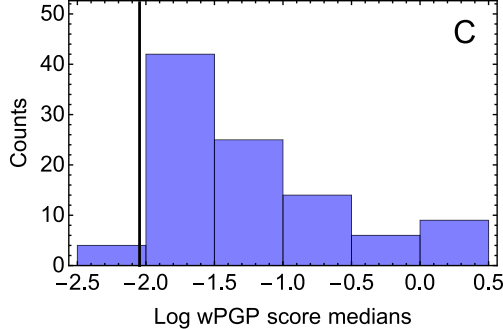

Supplement: S17 Fig — (A) The log-transformed wPGP score distributions for the 213 polymorphic genotypes from our study population (black), for 100 families of 213 artificial genotypes (pooled together) simulated under the neutral SFS (red), and for 100 families simulated under the population-derived SFS (blue). The dashed line marks the 2/3-quantile of the population score distribution, used for analysis in panel C. (B) The distributions of the family mean of the log-transformed wPGP scores for the two types of families. The black line marks the population value. p-values of the difference between the population and the randomly mutated genotypes: p = 0.01 for the neutral SFS and p = 0.57 for the population-derived SFS. (C) The distribution of the median wPGP scores calculated only for genotypes of strong effect on expression, i.e. genotypes with the score exceeding the value marked by the dashed line in panel A. The distribution corresponds to the genotypes randomly mutated under the population-derived SFS. p-value of the difference between the population and the randomly mutated genotypes: p < 0.01 for the population-derived SFS. (PDF) [file pone.0184657.s022.pdf]
