# Supplementary material for: Translating natural genetic variation to gene expression in a computational model of the Drosophila gap gene regulatory network
Source: PLoS One. 2017 Sep 12;12(9):e0184657. doi: 10.1371/journal.pone.0184657 (PMC5595321; doi:10.1371/journal.pone.0184657)
Supplement: S4 Fig — (A–C) For each group of SNP influence sign (columns), the panels present the population values (red lines) for the number of SNPs appearing in the overlapping regions of multiple TFBSs as a fraction of the total number of SNPs in the group. These values are compared with the distributions of the same fraction, but for positions randomly sampled from the model TFBSs. We simulate 1000 sets of such random positions, and for each set we count the fraction of positions appearing in the overlapping region of multiple TFBSs. The total number of positions in each set equals to the number of the population SNPs from a given sign group (36, 38, and 13 positions corresponding to the groups of purely positive, purely negative, and alternating sign, respectively). (D–F) The same as in (A–C) but for the fraction of positions appearing in overlapping TFBSs of the same regulatory type (mechanisms 1 and 2 from Fig 4A). Positions associated with the sign alternating SNPs from the population appear more often in the overlap regions of multiple TFBSs than expected by chance (C; p < 0.001), while the frequency of their appearance in TFBSs overlapping with other TFBSs of the same type is not distinguished from randomly sampled positions (F; p = 0.58). (PDF) [file pone.0184657.s009.pdf]

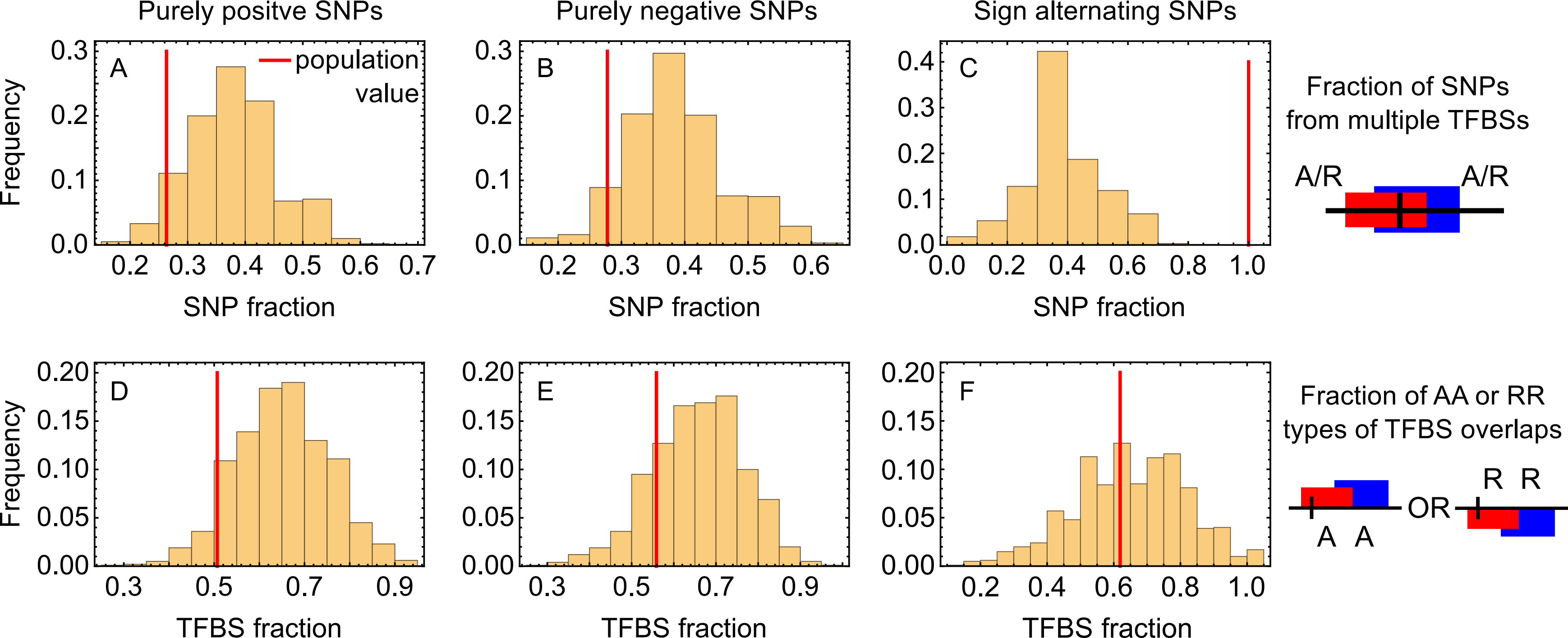
Purely positve SNPs
Purely negative SNPs
Sign alternating SNPs
A
B
C
D
E
F
population value
Frequency
SNP fraction
TFBS fraction
Fraction of SNPs from multiple TFBSs
A/R
A/R
Fraction of AA or RR types of TFBS overlaps
A A
OR
R R
0.0
0.1
0.2
0.3
0.4
0.00
0.05
0.10
0.15
0.20
0.2
0.3
0.4
0.5
0.6
0.7
0.0
0.2
0.4
0.6
0.8
1.0
0.3
0.5
0.7
0.9
